# Transannular Acylation Facilitates C_5_–C_9_ Bond Formation in Hyperforin Total Synthesis

**DOI:** 10.1021/acs.orglett.5c00243

**Published:** 2025-02-27

**Authors:** Julien A. König, Sebastian Frey, Bernd Morgenstern, Johann Jauch

**Affiliations:** † Organic Chemistry II, Saarland University, 66123 Saarbrücken, Germany; ‡ Service Center X-ray Diffraction, Saarland University, 66123 Saarbrücken, Germany

## Abstract

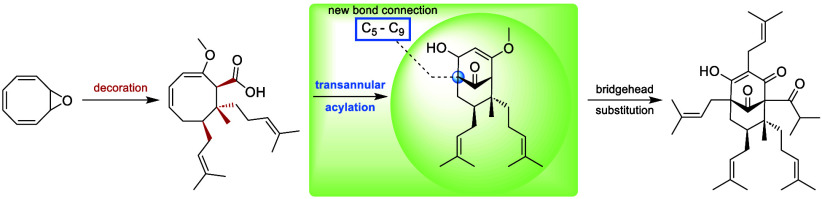

Hyperforin is considered the flagship congener among polycyclic polyprenylated acylphloroglucinols due to its compelling and complex molecular architecture, coupled with remarkable biological activity, thus rendering it an appealing synthetic target for chemists over the past two decades. Herein, an innovative linear total synthesis of hyperforin is reported. Our synthesis relies on the formation of the bicyclo[3.3.1]nonane-2,4,9-trione framework via transannular acylation of a decorated eight-membered ring, followed by late stage bridgehead substitution.

Polycyclic polyprenylated acylphloroglucinols (PPAPs) are meroterpenoid natural products found in plants of the *Hypericum* and *Garcinia* genera. Their distinctive and complex structure is composed of a densely substituted and highly oxygenated bicyclo[3.3.1]nonatrione core. This characteristic is accompanied by a broad diversity of biological activity. At present, over 1000 PPAPs are known.^1,2^

Hyperforin (**1**, Figure 1), found in St. John’s wort (*Hypericum perforatum*) and identified as one of its major bioactive constituents, was the first PPAP to be isolated and structurally elucidated.^3^ Since then, it has become the most prominent congener among the PPAPs. The attention paid to it stems from its unique structure, which highlights a quaternary stereogenic center in the vicinity of its one-carbon bridge, as well as its remarkable potency in various therapeutic areas, including its antidepressant,^4^ antibiotic,^5^ anti-inflammatory,^6^ and anticancer effects,^7^ and as a potential treatment for Alzheimer’s disease.^8,9^

**Figure 1:**
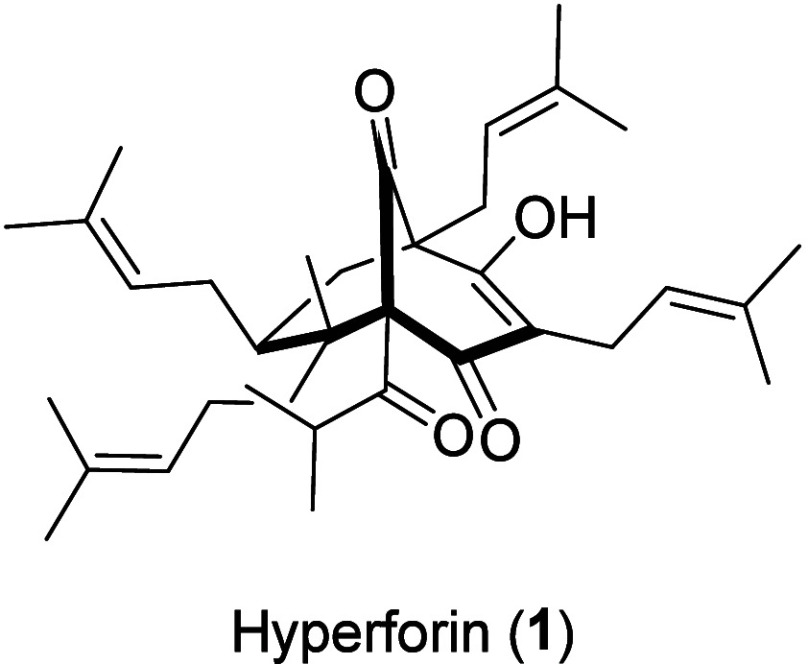
Structure of hyperforin (**1**).

Over the past few decades, several research groups have presented ingenious chemistry to successfully synthesize hyperforin (**1**).^10^ From our perspective, the bicyclo[3.3.1]nonane framework should be considered not just as two fused rings but also as a bridged eight-membered ring. Given that other PPAP total syntheses typically use six-membered precursors to construct the bicyclic core, this perspective suggests that an eight-membered ring strategy could be a crucial missing piece in the puzzle of PPAP syntheses. Encouraged by our recent success in synthesizing a simplified congener of **1** from cyclooctadiene,^11^ we decided to apply our approach to the more complex target hyperforin (**1**).

Our retrosynthetic idea can be divided into three major steps (Scheme 1). We envisioned that decorated carboxylic acid **3** could be derived from commercially available cyclooctatetraene (COT) (**4**) via multiple conjugate additions. The key intermediate, bicyclo[3.3.1]nonatrione **2**, should be generated through transannular cyclization, followed by consecutive oxidation. Finally, hyperforin (**1**) would be obtained after subsequent bridgehead substitution of C_5_, C_3_, and C_1_.

**Scheme 1:**
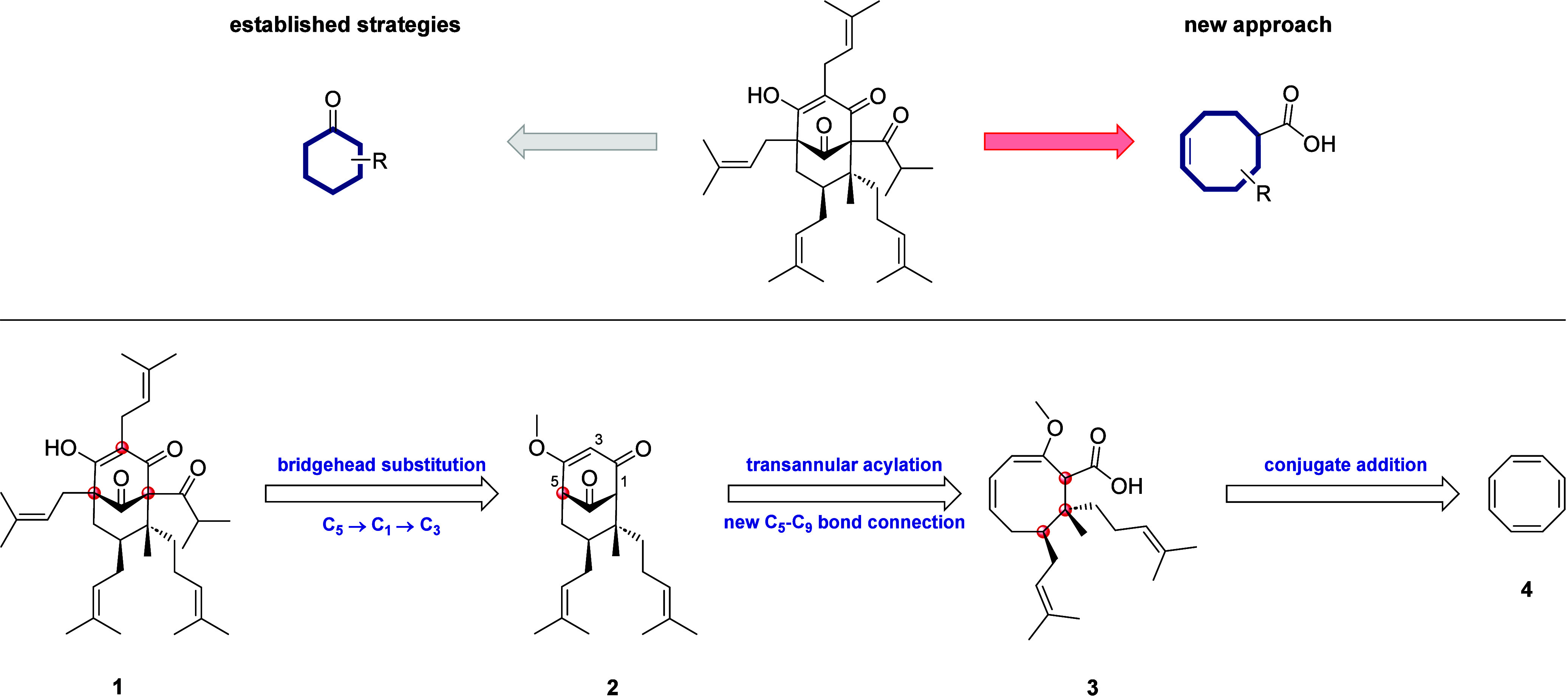
Retrosynthetic Idea

Pineschi reported the copper-catalyzed addition of common organometallic nucleophiles to COT-monoepoxide (**5**), followed by a thermally induced 1,5-sigmatropic hydrogen shift of the resulting allylic alcohols to their respective ketones.^12^ The group successfully introduced methyl, ethyl, and butyl moieties but did not report the introduction of any allylic substituents.^13^ We initially found that the overall yields of prenylated alcohol **6** were highly dependent on the quality of the Grignard reagent prepared prior to the reaction (Scheme 2). Extensive experimentation was necessary to identify the factors that ensure a potent solution of prenylmagnesium bromide.^14^

**Scheme 2:**
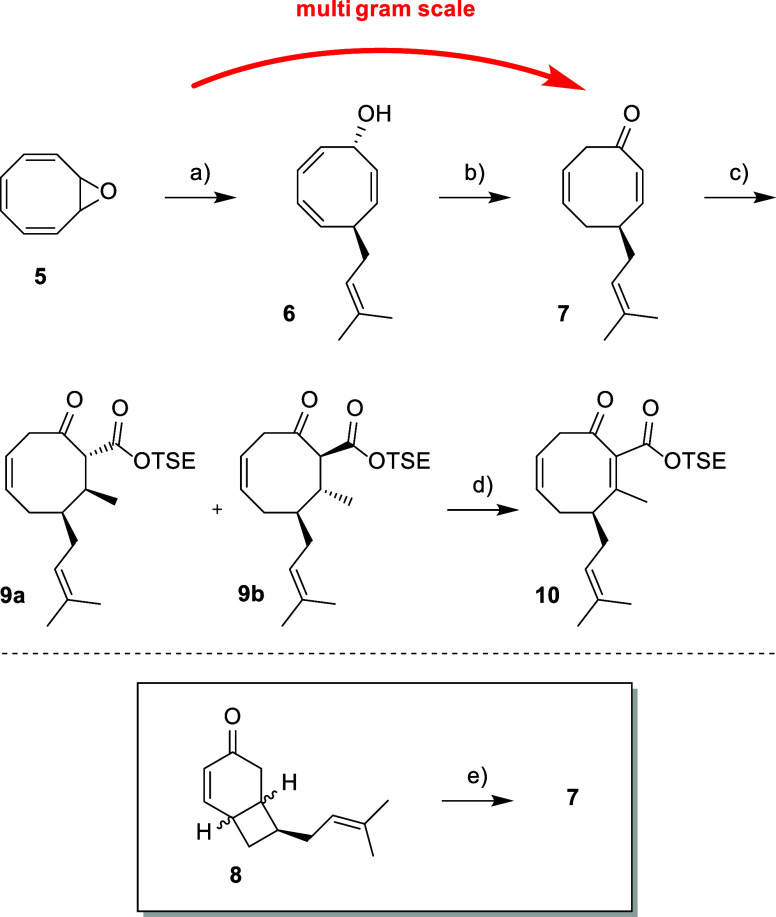
Decoration of the Core Structure Reagents and conditions: (a) CuCN, prenylmagnesium bromide, DCM, −78 °C, 6 h, 84%; (b) NEt_3_, benzene, reflux, overnight, 91%; (c) CuCN, MeLi, THF, −78 to −40 °C, 1 h, then 2-(trimethylsilyl)ethyl cyanoformate, −78 to −40 °C, 1.5 h, 95% dr 3.8:1 (**9a**/**9b**); (d) (1) NaH, THF, rt, 1 h, then PhSeCl, −100 to −78 °C, 3 h; (2) mCPBA, NEt_3_, 2-methyl-2-butene, DCM, −78 °C, 1.5 h, 68%; (e) LDA, THF, −78 °C to reflux, overnight, 57% (71% BRSM).

Among the copper sources tested (CuTC, Cu(OTf)_2_·C_6_H_6_, and CuCN), only CuCN resulted in any reaction. Allylic alcohol **6** was converted into ketone **7** directly after column chromatography due to its instability, even under an argon atmosphere at −18 °C.

When compound **6** was refluxed in toluene or heated neat to 80 °C, ketones **7** and **8** were obtained. The yields and ratio of the ketones were not reproducible, but ketone **8** was generally the main product. Bicyclic ketone **8** results from a 6π-electrocyclic ring closure of the enol form of **7**.

We envisaged that unreacted alcohol **6** facilitates enolization, as ketone **7** remained unreactive in refluxing toluene. However, in the presence of octanol, the formation of ketone **8** was observed by TLC. The tautomeric equilibrium could be shifted in favor of ketone **7** with 1.1 equiv of NEt_3_, which acted as a hydrogen bonding acceptor during the reaction. This adjustment resulted in an excellent yield of 91% for ketone **7**. From a practical perspective, any byproduct **8** could be collected and subjected to the reverse 6π-electrocyclic ring opening. Under the optimized conditions, we were able to easily synthesize **7** from cyclooctatetraene (**4**) on a multigram scale. Simple copper-mediated conjugate addition and subsequent trapping of the respective enolate with 2-(trimethylsilyl)ethyl (TSE) cyanoformate furnished β-keto ester **9** in an excellent 95% yield. To ensure the *syn* conformation of the selenide and the proton at the tertiary center in our double bond regeneration sequence, **9** was reacted slowly with PhSeCl at −100 °C. The crude product was then oxidized with mCPBA,^15^ with excess 2-methyl-2-butene and NEt_3_ added at the end to mimic the prenyl moiety and prevent over-oxidation. Considering the two adjacent tertiary centers of **9**, we were pleased with the 68% overall yield of doubly activated Michael acceptor **10**.

Initial attempts to implement the homoprenylic side chain were unsuccessful. Reactions conducted in the presence of Cu(OTf)_2_·C_6_H_6_ or CuCN yielded no product. Merely, degradation of starting material **10** could be observed above −30 °C. With CuBr·SMe_2_ at −40 °C, the desired ester **11** was obtained in 47% yield, accompanied by 20% of enol **12** (Table 1, entry 1). The latter results from vinylogous deprotonation, followed by double bond isomerization.

**Table 1:**
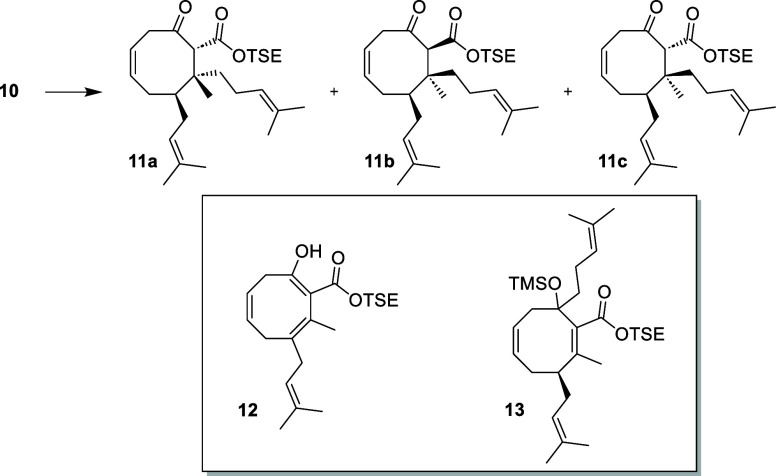

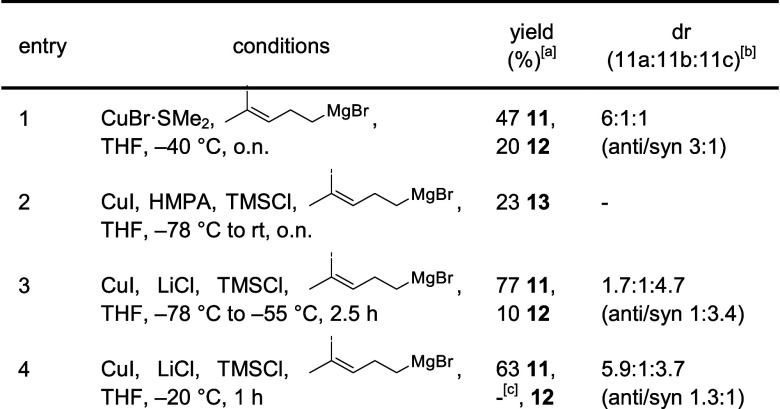
Excerpt of the Optimization Work for the Second Conjugate Addition

a Isolated yield.

b Determined by NMR.

c Not determined.

Intense NMR analysis and multiple chromatography steps revealed that **11** was obtained as a mixture of three diastereomers; **11c** can be separated using petroleum ether and DCM in a 1:2 ratio. NOESY experiments led to the structural assignments, as shown in Table 1. Although the desired diastereomer **11d** was not formed in this reaction, the good *anti*:*syn* ratio of 3:1, referring to the relative configuration of the prenyl and homoprenyl substituents, would ultimately allow us to generate **11d** from **11a** through epimerization. We initiated optimization work using TMSCl as an activating agent and HMPA to enhance the nucleophilicity. Under these conditions, the sole product obtained was silyl ether **13** (Table 1, entry 2).

Inspired by the work of Stoffman and Clive,^16^ which involved adding allylmagnesium bromide to an equally activated β-ketoester with great yields, we explored the use of soluble CuI with LiCl and TMSCl.^17^ Under these conditions at −78 °C, yields increased to 77%, although the *anti*:*syn* ratio decreased to 1:3.4 (Table 1, entry 3). To determine whether the change in the *anti*:*syn* ratio was due to Lewis acid activation or temperature dependency, we repeated the experiment at −20 °C. At this temperature, **11** was obtained in 63% yield with an *anti*:*syn* ratio of 1.3:1 (Table 1, entry 4). Additionally, attempts using catalytic quantities of copper did not lead to any reaction as did the change from homoprenylmagnesium bromide to bishomoprenyl zinc.^18^

The diastereomeric mixture of **11a**–**c** was next subjected to the thermodynamic equilibration of epimers (Scheme 3). The desired diastereomer **11d** was obtained in 70% yield for each cycle of epimerization, based on the amount of **11a** present in the recovered diastereomeric mixture of **11a**–**c**. Fortunately, diastereomer **11d** was found to be completely separable from the mixture. To further substantiate the aforementioned structural assumptions, we proceeded to investigate the diastereomers of **11** separately. Most likely due to double bond conjugation, we found that enol ether formation is favored over acetalization under acidic conditions in methanol.

**Scheme 3:**
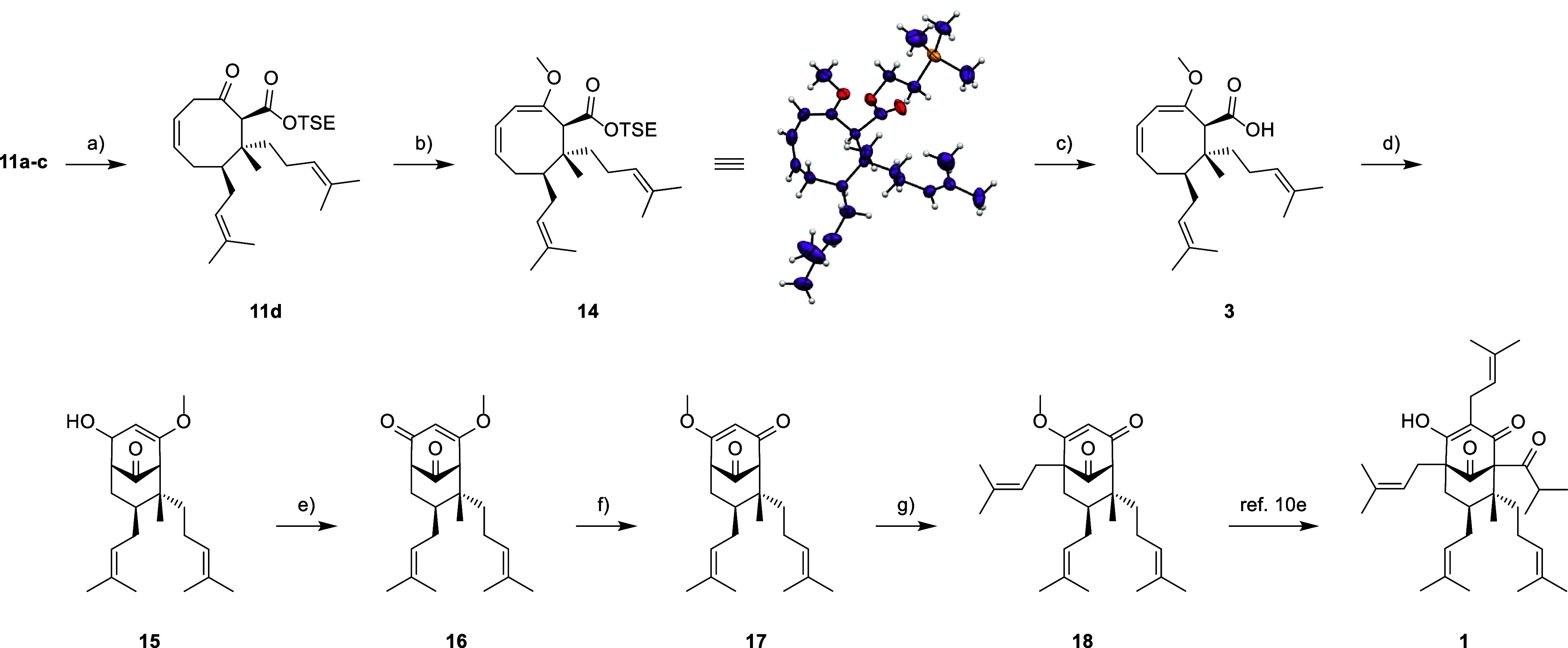
Finalization of Hyperforin (**1**) Total Synthesis Reagents and conditions: (a) DBU, THF, reflux, 20 h, 70% BRSM; (b) (±)-CSA, HC(OMe)_3_, MeOH, 60 °C, 3.5 h, 63% BRSM; (c) TBAF, THF, 40 °C, 2.5 h, 98%; (d) 2,6-di-*tert*-butyl-4-methylpyridine, TFAA, CHCl_3_, −40 °C, 30 min, then saturated K_2_CO_3_, rt, 1 h, 69% dr 1.4:1.0 (*exo*/*endo*); (e) PCC, NaOAc, DCM, 0 °C to rt, 1 h, 73%; (f) PTSA, HC(OMe)_3_, MeOH, 50 °C, 42–50 h, then HCl, THF, 50 °C, 25 min, 70%; (g) Cy_2_NLi, prenyl bromide, THF, −78 °C, 15 min, 73%.

Diastereomer **11a** with minor impurities of **11b**, separated isomer **11c**, and separated isomer **11d** were reacted with substoichiometric amounts of (±)-CSA and 2 equiv of HC(OMe)_3_ in methanol. After 2 h, we observed full conversion of **11a** as well as **11c**, both resulting in a 67% yield of their respective enol ethers. Surprisingly, **11d** reacted much more slowly, yielding only 11% of enol ether **14** and 57% of the recovered starting material. Satisfyingly, at this point we were able to confirm the assigned relative configuration of **11d** by X-ray crystallography of **14**. Ultimately, optimization of the enol ether formation of **11d** was necessary. The best results were achieved using 1 equiv of (±)-CSA and terminating the reaction after 3.5 h to prevent decomposition of both the product and the starting material. Changing the acid to PTSA or 5-sulfosalicylic acid gave the same results with slightly diminished yields. Catalytic amounts of H_2_SO_4_, AcOH, TFA, Amberlyst 15, or HCl in 1,4-dioxane proved to be futile, as most of the product and starting material degraded. Due to the more acidic proton at C_1_, any efforts to generate **14** via anionic pathways were unsuccesful. Although Lewis acid-catalyzed enol ether formation with TiCl_4_ in methanol^19^ worked well for diastereomer **11c**, delivering 60% of the respective enol ether and 33% of the re-isolated starting material, no major product formation for diastereomer **11d** was observed by TLC or NMR before decomposition.

With **11d** in hand, we released carboxylic acid **3** in nearly quantitative yield. Unlike our initial cyclization protocol,^20^ the addition of 2,6-di-*tert*-butyl-4-methylpyridine^21^ to the natural product scaffold and the use of lower reaction temperatures were crucial to avoid side reactions with intermediate cationic species. The resulting 69% yield of allylic alcohol **15** is comparable to the result from our model studies. Following this, the next step involved the oxidation of **15** to ketone **16**. Standard procedures such as Swern, TPAP, hypervalent iodine, and manganese oxidants yielded only trace amounts of ketone **16**. This finding aligns with literature reports on the difficulties of oxidizing a bicyclic PPAP scaffold to its 1,3,5-trione system.^22^ However, we were pleased to find that **15** could be readily oxidized by using PCC with NaOAc as an additive.

Bicyclo[3.3.1]nontraiones without any bridgehead substituents are rare in PPAP chemistry, and to the best of our knowledge, only one total synthesis based on a respective intermediate has been reported.^23^ While direct prenylation of C_5_ would yield an intermediate known from Barriault’s synthesis of hyperforin (**1**),^10d^ we chose to isomerize vinylogous ester **16** to its regioisomer. On the basis of previous reports, regioisomer **17** should be the thermodynamically favored product and expected to react more readily.^24^ Isomerization was carried out under standard conditions with the addition of HCl at the end to cleave the dimethyl acetal formed simultaneously at C_9_ during the reaction.^25^

Next, we turned to bridgehead substitution. While LDA and LTMP are commonly used bases for this transformation, both proved to be ineffective in our case. LTMP led to complete decomposition, and LDA resulted in the reduction of the C_9_ ketone and a poor 37% yield of the desired product **18**.^26^

Fortunately, we found that using just 2 equiv of the rather unusual lithium dicyclohexylamide in the reaction afforded **18** in a good 73% yield. In light of Maimone’s previous work, we successfully completed our synthetic venture from **18**.^10e^ The sequence involving C_3_ chlorination, C_1_ acylation, metal–halogen exchange enabling substitution at C_3_, and Krapcho-type demethylation proceeded as described.

In conclusion, we successfully synthesized the complex flagship PPAP hyperforin (**1**) in 17 steps from commercially available cyclooctatetraene (**4**). The presented strategy provides significant insights into cyclooctane chemistry, demonstrates a salient approach to selective C_5_–C_9_ bond formation in PPAP chemistry via transannular acylation, and employs an adaptive intermediate that offers full control over derivative-specifying positions around the bicyclo[3.3.1]nonane framework. We look forward to applying this strategy to synthesize other prominent yet untouched PPAPs.

## Data Availability

The data underlying this study are available in the published article and its Supporting Information.

## Supporting Information Available

Experimental details, characterization data for all new compounds, ^1^H and ^13^C NMR spectra, and X-ray data (PDF)

## Dedication

This article is dedicated to Prof. Dr. Volker Schurig on the occasion of his 85th birthday.
