# Supplementary material for: Transannular Acylation Facilitates C5–C9 Bond Formation in Hyperforin Total Synthesis
Source: Org Lett. 2025 Feb 27;27(9):2157–62. doi: 10.1021/acs.orglett.5c00243 (PMC11894654; doi:10.1021/acs.orglett.5c00243)
Supplement: Supplementary file 1 — ol5c00243_si_001.pdf [file ol5c00243_si_001.pdf]

Supporting Information for

Transannular Acylation Facilitates Novel C_5–C_9 Bond Formation in Hyperforin Total Synthesis

Julien A. König[a], Sebastian Frey[a], Bernd Morgenstern[b] and Johann Jauch[a]*

[a] Organic Chemistry II, Saarland University, D-66123 Saarbrücken, Germany;
[b] Service Center X-Ray Diffraction, Saarland University, D-66123 Saarbrücken, Germany

*E-Mail: j.jauch@mx.uni-saarland.de

Table of Content

GENERAL 2

EXPERIMENTAL PROCEDURES 3

COMPARISON OF NATURAL AND SYNTHETIC HYPERFORIN 15

^{1}H AND ^{13}C NMR SPECTRA 20

X-RAY CRYSTALLOGRAPHY 34

General

All reactions were performed under inert gas atmosphere (Ar or N_2) using standard *Schlenk* techniques in flame-dried glassware equipped with a magnetic stir bar unless otherwise stated. All solvents were freshly distilled prior to use; Et_2O and THF over sodium and benzophenone; DCM and amines over calcium hydride; MeOH over magnesium. Prenyl bromide was distilled in vacuo prior to use. LiCl was dried overnight in a 120 °C oil bath under high vacuum. All other commercial reagents were used without purification unless otherwise noted. Reactions were monitored by thin layer chromatography (TLC) analysis using glass plates pre-coated with silica gel 60 F_{254} (Merck). Thin layer chromatography plates were viewed under UV light with a wavelength of 254 nm and stained with iodine on silica or *p*-anisaldehyde staining solution. Column chromatography was performed with silica gel 60 (40-63 µm particle size, Merck). ^1H and ^{13}C nuclear magnetic resonance (NMR) spectra were recorded in deuterated solvents on Bruker Avance II 400 MHz and Bruker Avance I 500 MHz spectrometers. Natural product spectra were recorded in deuterated solvents on Bruker Neo 500 MHz. Chemical shifts were reported in ppm on the δ scale relative to residual $CHCl_3$ (δ = 7.26 for ^1H NMR and δ = 77.0 for ^{13}C NMR) or MeOH (δ = 3.31 for ^1H NMR and δ = 49.0 for ^{13}C NMR) as an internal reference. High resolution mass spectrometry (HRMS) was performed by the service center for mass spectrometry of Saarland University on a Solarix FT-ICR-MS 7T by Bruker Daltonik GmbH. Single crystal X-ray diffraction analysis was carried out by the service center for X-ray diffraction of Saarland University on a Bruker D8 Advance and Bruker X8 ApexII diffractometer.

Experimental procedures

Cyclooctatetraenmonoepoxide (5)[1]

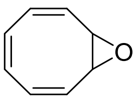

Amylen is removed from $CHCl_3$ prior to use as followed: In a separatory funnel $CHCl_3$ is throughroughly mixed with H_2SO_4, separated, and filtered through a short pad of basic Al_2O_3 (activity I).

To a solution of 12.5 g (120 mmol) cyclooctatetraene (**4**) in $CHCl_3$ (375 mL) in a one-neck round-bottom flask, was added 26.6 g (108 mmol) mCPBA dissolved in $CHCl_3$ (300 mL) at 0 °C over a period of two hours. The solution was stirred overnight and allowed to warm to room temperature. The resulting suspension is poured into a mixture of 2 M NaOH (100 mL) and saturated aq. $Na_2S_2O_3$ (30 mL). The layers were separated, and the aqueous layer was extracted thrice with $CHCl_3$. The combined organic extracts were dried over anhydrous $MgSO_4$ and concentrated gently in a 65 °C water bath. Purification of the residue by flash column chromatography (^{n}Pen/Et_2O = 10:1) and gentle removal of the solvent in a 40 °C water bath afforded 9.63 g (80.2 mmol, 74%) of **4** as a pale-yellow liquid.

R_f 0.35 (^{n}Pen/Et_2O = 10:1).

^{1}H NMR (400 MHz, $CDCl_3$) δ 6.12-6.09 (d, *J* = 11.6, 2H), 6.02-5.99 (d, *J* = 11.7, 2H), 5.92 (s, 2H), 3.48 (s, 2H).

^{13}C NMR (100 MHz, $CDCl_3$) δ 128.0, 126.4, 125.7, 55.3.

6-(3-Methylbut-2-en-1-yl)cycloocta-2,4,7-trien-1-ol (6)

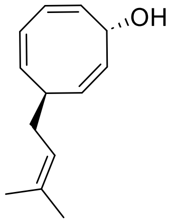

Prenylmagnesium bromide was freshly prepared as follows: In a 250 mL one-neck round-bottom flask with a triangular stirring bar 29.2 g (1.20 mol) magnesia powder (–20+100 mesh) was suspended in Et_2O (50 mL) and 22.3 g (150 mmol) of freshly distilled prenyl bromide was added via a dropping funnel over a period of six hours. Over the entire course of the reaction, it was necessary to stir the solution continuously, vigorously, and evenly. The whole magnesium should be in motion all the time without the powder splashing out of the solution. For us 400 rpm worked perfectly with the described triangular stirring bar.[2] After complete addition the mixture was stirred another 30 minutes. Stirring was stopped and an aliquot (1 mL) of the above magnesium level layer solution was added to 0.1 M HCl (20 mL) and titrated against

---

[1] A slightly modified procedure was used. Otherwise, see: a) F. Del Moro, P. Crotti, V. Di Bussolo, F. Macchia, M. Pineschi, *Org. Lett.* **2003**, *5*, 1971–1974; b) M. Pineschi, F. Del Moro, P. Crotti, F. Macchia, *Eur. J. Org. Chem*. **2004**, 4614–4620

[2] Even small irregularities in dropping speed or stirring can degrade the quality of the prepared reagent. Noteworthy is that if a biphasic solution is obtained the reagent is surely useless. We assume this to be a result of excess $MgBr_2$ and Wurtz-type by-product. High concentrations of the latter led to phase separations and neither the Wurtz-type phase nor the etheral phase gave sufficient yields of the desired product.

0.1 M NaOH with phenolphthalein as an indicator. Only concentrations between 0.5 M and 0.65 M of the Grignard solution were suitable for the reaction.

In a three-neck round-bottom flask, 898 mg (10.0 mmol) CuCN was suspended in DCM (50 mL) and cooled to –78 °C. 5.30 g (44.1 mmol) of the epoxide **5** in DCM (100 mL) was added over 15 minutes. Then 125 mL (72.5 mmol, 0.58 M) of the Grignard solution was diluted with DCM (120 mL) and added over a period of six hours. The reaction was allowed to warm up to –60 °C overnight. Following this the mixture was warmed to –40 °C and treated with saturated aq. NH_4Cl. The layers were separated, and the aqueous layer was extracted thrice with DCM. The combined organic extracts were dried over anhydrous $MgSO_4$ and concentrated in vacuo. Purification of the residue by flash column chromatography (^{n}Pen/Et_2O = 3:1) afforded 7.06 g (37.1 mmol, 84%) **6** as a yellow oil.

R_f 0.21 (^{n}Pen/Et_2O = 3:1).

^{1}H NMR (400 MHz, $CDCl_3$) δ 6.13-6.08 (m, 2H), 5.58 (dtr, *J* = 11.2 Hz, 2.3 Hz, 1H), 5.40 (dtr, *J* = 11.2 Hz, 2.8 Hz, 1H) 5.36 (dd, *J* = 10.2 Hz, 5.5 Hz, 1H), 5.19 (dd, *J* = 9.7 Hz, 6.8 Hz, 1H), 5.10 (trsept, *J* = 7.0 Hz, 1.3 Hz, 1H), 4.88 (bs, 1H), 2.89-2.82 (m, 1H), 2.24 (tr, *J* = 7.1 Hz, 2H), 2.08 (s, 1H, OH), 1.70 (s, 3H), 1.63 (s, 3H).

^{13}C NMR (100 MHz, $CDCl_3$) δ 133.4, 133.0, 132.0, 131.5), 130.8, 127.5, 126.2, 122.1, 69.8, 37.7, 34.1, 25.8, 18.01.

HRMS (ESI) *m/z*: $[M - H]^-$ calcd for $C_{13}H_{17}O$ 189.1285; found 189.1279.

4-(3-Methylbut-2-en-1-yl)cycloocta-2,6-dien-1-one (7)

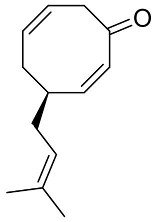

In a one-neck round-bottom flask, 15.5 g (81.2 mmol) of the allylic alcohol **6** was dissolved in benzene (80 mL). 12.5 mL (89.7 mmol) NEt_3 was added, and the solution is stirred overnight at 80 °C. The resulting deep brown solution is concentrated in vacuo. Purification of the residue by flash column chromatography (^{n}Pen/Et_2O = 10:1) afforded 14.2 g (74.6 mmol, 91%) **7** as a yellow to red oil.

R_f 0.29 (^{n}Pen/Et_2O = 10:1).

^{1}H NMR (400 MHz, $CDCl_3$) δ 6.09 (dd, *J* = 12.3 Hz, 7.6 Hz, 1H, 5.96-5.93 (m, 1H), 5.64-5.59 (m, 1H), 5.57-5.50 (m, 1H), 5.11 (trsept, *J* = 7.2 Hz, 1.2 Hz, 1H), 3.78-3.72 (m, 1H), 3.31-3.21 (m, 1H), 2.98 (dd, *J* = 14.6 Hz, 8.0 Hz, 1H), 2.50 (dq, *J* = 12.8 Hz, 2.9 Hz, 1H), 2.14 (tr, *J* = 6.9 Hz, 2H), 1.97 (ddd, *J* = 18.5 Hz, 13.0 Hz, 5.7 Hz, 1H), 1.71 (s, 3H), 1.63 (s, 3H).

^{13}C NMR (100 MHz, $CDCl_3$) δ 201.6, 147.9, 134.1, 131.3, 130.2, 121.2, 120.9, 44.3, 38.3, 35.5, 34.0, 25.8, 18.0.

HRMS (ESI) *m/z*: [M + NH_4]$^+$ calcd for $C_{13}H_{22}NO$ 208.1696; found 208.1695.

2-(Trimethylsilyl)ethyl-8-methyl-7-(3-methylbut-2-en-1-yl)-2-oxocyclooct-4-ene-1-carboxylate (9)

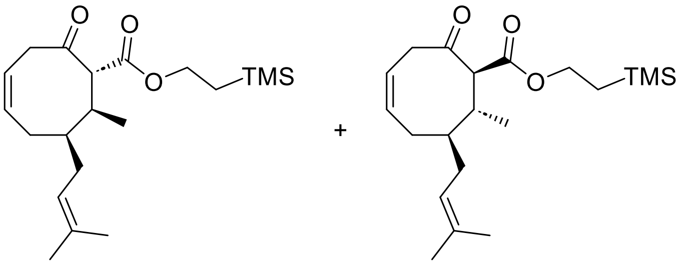

9a major **9b minor**

In a three-neck round-bottom flask, 1.75 g (19.4 mmol) CuCN was suspended in Et_2O (65 mL) and cooled to –78 °C. 20.5 mL (32.8 mmol, 1.6 M in Et_2O) MeLi was added dropwise over a period of ten minutes. The dry ice bath was replaced with an ice bath and the suspension is kept at 0 °C until a clear solution was obtained. Then the reaction is cooled back to –78 °C. 2.85 g (15.0 mmol) of ketone **7** in Et_2O (15 mL) was added over 15 minutes. The resulting orange to yellow solution was kept at –78 °C for 30 minutes and then warmed to –40 °C. After one hour at –40 °C the reaction is re-cooled to –78 °C. 3.33 g (19.4 mmol) 2-(trimethylsilyl)ethyl cyanoformate[3] was quickly added neat in one portion to the vigorously stirred solution. After one hour the reaction mixture was warmed to –40 °C and kept at this temperature for an additional 30 minutes. Following this the reaction was treated with saturated aq. NH_4Cl. Any solids were filtered off. The layers were separated, and the aqueous layer was extracted thrice with Et_2O. The combined organic extracts were washed with saturated aq. NH_4Cl, H_2O and brine and then dried over anhydrous $MgSO_4$ and concentrated in vacuo. Purification of the residue by flash column chromatography (^{n}Pen/Et_2O = 20:1 → 10:1) afforded 4.97 g (14.2 mmol, 95%, 3.8:1 dr) of **9** as a colorless oil and mixture of two diastereomers.[4]

9a (major)

$\mathbf{R_f}$ 0.35 (^{n}Pen/Et_2O = 10:1).

^{1}**H** NMR (400 MHz, $CDCl_3$) δ 5.67-5.56 (m, 2H), 5.03-5.00 (m, 1H), 4.20-4.09 (m, 2H), 3.61 (d, *J* = 11.1 Hz, 1H), 3.55 (dd, *J* = 17.9 Hz, 4.6 Hz, 1H), 3.02 (dd, *J* = 18.0 Hz, 4.5 Hz, 1H), 2.78 (dtrq, *J* = 10.6 Hz, 6.9 Hz, 3.5 Hz, 1H), 2.12 (dtr, *J* = 15.5 Hz, 4.4 Hz, 1H), 2.06-2.00 (m, 1H), 1.88-1.72 (m, 2H), 1.69-1.63 (m, 1H), 1.66 (s, 3H), 1.55 (s, 3H), 0.98-0.95 (m, 2H), 0.93 (d *J* = 6.8 Hz, 3H), 0.01 (s, 9H).

^{13}**C** NMR (100 MHz, $CDCl_3$) δ 208.1, 170.1, 132.9, 129.9, 124.7, 122.7, 63.7, 63.5, 43.7, 41.5, 35.3, 30.3, 29.3, 25.8, 17.8, 17.2, 15.6, -1.6.

HRMS (ESI) *m/z*: [M + NH_4]$^+$ calcd for $C_{20}H_{38}NO_3Si$ 368.2616; found 368.2613.

---

[3] For the preparation of 2-(trimethylsilyl)ethyl cyanoformate, see: a) M. Sekine, M. Tobe, T. Nagayama, T. Wada, *Lett. Org. Chem.* **2004**, *1*, 179–182; b) N. J. Foy, S. V. Pronin, *J. Am. Chem. Soc.* **2022**, *144*, 10174–10179.

[4] Diastereomers were separable but only separated once for NMR analysis.

9b (minor)

R_f 0.29 (^{n}Pen/Et_2O = 10:1).

^{1}H NMR (400 MHz, $CDCl_3$) δ 5.66-5.53 (m, 2H), 5.04 (trsept, J = 5.8 Hz, 1.4 Hz, 1H), 4.17-4.11 (m, 2H), 3.64 (d, J = 11.5 Hz, 1H), 3.53-3.47 (m, 1H), 3.02 (dd, J = 17.4 Hz, 5.8 Hz, 1H), 2.31-2.20 (m, 1H), 2.08-1.74 (m, 3H, H6), 1.68 (s, 3H), 1.57 (s, 3H,), 1.27-1.19 (m, 1H), 0.99 (d, J = 6.5 Hz, 3H), 0.98-0.91 (m, 2H), 0.01 (s, 9H).

^{13}C NMR (100 MHz, $CDCl_3$) δ 208.1, 169.6, 133.1, 129.9, 124.0, 122.5, 65.6, 63.4, 45.3, 44.6, 38.1, 31.6, 28.5, 25.8, 17.9, 17.8, 17.2, -1.6.

HRMS (ESI) *m/z*: $[M + NH_4]^+$ calcd for $C_{20}H_{38}NO_3Si$ 368.2616; found 368.2618.

2-(Trimethylsilyl)ethyl-2-methyl-3-(3-methylbut-2-en-1-yl)-8-oxocycloocta-1,5-diene-1-carboxylate (10)

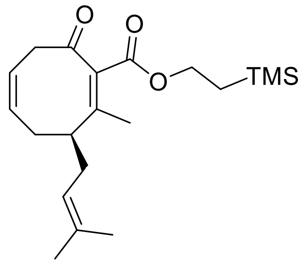

A three-neck round-bottom flask was charged with 223 mg (5.58 mmol, 60% on mineral oil) NaH. The reagent was washed two times with THF and then suspended in THF (11 mL). 1.75 g (5.00 mmol) of the β-ketoester **9** in THF (6 mL) was added. The mixture was stirred at room temperatur until no more gas evolution was observed and a clear orange solution was obtained. The solution was cooled to –100 °C. 1.27 g (6.65 mmol) PhSeCl in THF (34 mL) was added over a period of three hours. The internal temperature was checked with an electric internal temperature sensor over the whole course of addition and should not exceed –100 °C. Afterwards the red solution was warmed to –78 °C and kept at this temperature for an additional hour. The reaction was treated with saturated aq. $NaHCO_3$. The layers were separated and extracted thrice with Et_2O. The combined organic extracts were dried over anhydrous $MgSO_4$ and concentrated in vacuo.

The residue was dissolved in DCM (33 mL) in a one-neck round-bottom flask and cooled to –78 °C. 1.84 g (7.46 mmol, 70% - 75%) mCPBA in DCM (18 mL) was added over 45 minutes. The reaction was kept at –78 °C for one and a half hour. Then 5.5 mL (51.7 mmol) 2-methyl-2-butene and 7 mL (50.5 mmol) NEt_3 was added before warming to room temperature. The reaction was treated with saturated aq. $NaHCO_3$. The layers were separated, and the aqueous layer was extracted thrice with DCM. The combined organic extracts were dried over anhydrous $MgSO_4$ and concentrated in vacuo. Purification of the residue by flash column chromatography (^{n}Pen/Et_2O = 20:1 → 10:1) afforded 1.21 g (3.47 mmol, 68%) of the unsaturated β-ketoester **10** as a pale-yellow oil.

R_f 0.19 (^{n}Pen/Et_2O = 20:1).

^{1}H NMR (400 MHz, $CDCl_3$) δ 5.70-5.65 (m, 2H), 5.07-5.02(m, 1H), 4.25-4.12 (m, 2H), 3.37-3.30 (m, 1H), 3.25-3.16 (m, 1H), 2.40-2.33 (m, 1H), 2.21-2.14 (m, 1H), 2.12 (s,

3H), 2.10-1.93 (m, 2H), 1.69 (s, 3H), 1.59 (s, 3H), 0.99 (ddd, J = 9.5 Hz, 7.8 Hz, 1.5 Hz, 2H), 0.02 (s, 9H).

^{13}C NMR (100 MHz, $CDCl_3$) δ 207.1, 164.8, 155.6, 134.1, 131.2, 129.0, 124,5, 121.4, 62.9, 49.1, 45.0, 32.8, 30.1, 25.8, 20.9, 17.9, 17.3, -1.6.

HRMS (ESI) *m/z*: $[M + Na]^+$ calcd for $C_{20}H_{32}NaO_3Si$ 371.2013; found 371.2005.

2-(Trimethylsilyl)ethyl-8-methyl-7-(3-methylbut-2-en-1-yl)-8-(4-methylpent-3-en-1-yl)-2-oxocyclooct-4-ene-1-carboxylate (11)

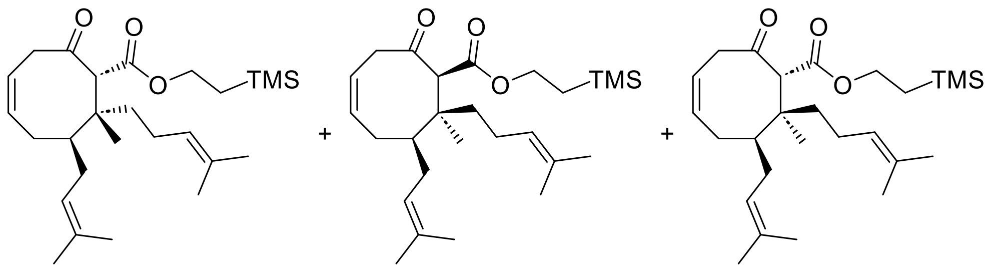

11a major **11b minor** **11c minor**

Homoprenylmagneiusm bromide was freshly prepared prior to use as follows: A one-neck round-bottom flask equipped with a reflux condenser was charged with 2.70 g (111 mmol) magnesia powder (–20+100 mesh) and suspended in Et_2O (68 mL). For activation, ten drops 1,2-dibromoethane and ten drops of homoprenyl bromide were added at room temperature. The mixture is stirred for five minutes, after which 12.3 g (75.5 mmol) homoprenyl bromide was added over a period of 90 minutes using a syringe pump. An aliquot (1 mL) of the solution was added to 0.1 M HCl (20 mL) and titrated against 0.1 M NaOH with phenolphthalein as an indicator.

A three-neck round-bottom flask with an electric internal temperature sensor was charged with 154 mg (3.63 mmol) dry LiCl and 610 mg (3.20 mmol) CuI. The flask was evacuated and refilled with N_2 two times. THF (20 mL) was added, and the suspension was stirred until a yellow solution was obtained. The solution was cooled to –20 °C. 5 mL (6.00 mmol, 1.2 M in Et_2O) of the Grignard solution was added over five minutes so that the internal temperature did not exceed –15 °C. The now deep blue solution was stirred for five minutes before adding 0.4 mL (3.15 mmol) freshly distilled TMSCl in one portion. After an additional five minutes 522 mg (1.50 mmol) **10** in THF (8 mL) was added over three minutes. Over a period of 30 minutes the mixture was allowed to warm to –10 °C. The reaction was treated with a mixture of saturated aq. NH_4Cl and 32% ammonia solution (9:1). The layers were separated, and the aqueous layer was extracted thrice with Et_2O. The combined organic extracts were dried over anhydrous $MgSO_4$ and concentrated in vacuo. Purification of the residue by flash column chromatography (^{n}Pen/Et_2O = 30:1 → 20:1) afforded 407 mg (0.94 mmol, 63%, 6:1:4 dr) of **11** as a colorless oil and mixture of three diastereomers.

11a (major)

$\mathbf{R_f}$ 0.29 (^{n}Pen/Et_2O = 20:1).

$\mathbf{R_f}$ 0.45 (^{n}Pen/DCM = 1:2).

^{1}**H** NMR (400 MHz, $CDCl_3$) δ 5.77-5.68 (m, 1H), 5.53-5.46 (m, 1H), 5.14-5.09 (m, 1H), 5.03-5.03 (m, 1H), 4.20-4.04 (m, 2H), 3.56 (s, 1H), 3.21 (dd, *J* = 19.1 Hz, 6.6 Hz, 1H), 3.09 (dd, *J* = 19.1 Hz, 5.1 Hz, 1H), 2.13-1.50 (m, 9H), 1.71 (s, 3H), 1.65 (s, 3H), 1.60 (s, 3H), 1.56 (s, 3H), 0.99-0.91 (m, 2H), 0.94 (s, 3H), 0.03 (s, 9H).

^{13}**C** NMR (100 MHz, $CDCl_3$) δ 206.2; 168.7; 134.7; 132.8); 131.5; 124.4; 123.9; 121.8; 62.9; 62.7; 43.3; 42.9; 42.3; 38.1; 28.5; 27.9; 25.9; 25.7; 22.8; 20.0; 18.0; 17.5; 17.3, -1.6.

HRMS (ESI) *m/z*: $[M + H]^+$ calcd for $C_{26}H_{45}O_3Si$ 433.3133; found 433.3129.

11b (minor)

$\mathbf{R_f}$ 0.29 (^{n}Pen/Et_2O = 20:1).

$\mathbf{R_f}$ 0.45 (^{n}Pen/DCM = 1:2).

^{1}**H** NMR (500 MHz, $CDCl_3$) δ 5.83-5.71 (m, 1H), 5.54-5.48 (m, 1H), 5.16 (tsept, *J* = 6.9 Hz, 1.3 Hz, 1H), 5.07-5.03 (m, 1H), 4.17-4.07 (m, 2H), 3.85 (s, 1H), 3.20-3.07 (m, 2H), 2.26-1.79 (m, 9H), 1.72 (s, 3H), 1.68 (s, 3H), 1.64 (s, 3H), 1.60 (s, 3H), 1.12 (s, 3H), 0.98-0.91 (m, 2H), 0.02 (s, 9H).

^{13}**C** NMR (125 MHz, $CDCl_3$) δ 205.4, 168.6, 134.5, 133.3, 130.8, 125.4, 123.7, 121.4, 63.6, 63.1, 45.5, 43.9, 43.7, 30.3, 28.3, 27.0, 25.9, 25.7, 24.4, 23.9, 18.1, 17.7, 17.2, -1.6.

HRMS (ESI) *m/z*: $[M + H]^+$ calcd for $C_{26}H_{45}O_3Si$ 433.3133; found 433.3129.

11c (minor)

$\mathbf{R_f}$ 0.29 (^{n}Pen/Et_2O = 20:1).

$\mathbf{R_f}$ 0.41 (^{n}Pen/DCM = 1:2).

^{1}**H** NMR (400 MHz, $CDCl_3$) δ 5.70 (q, *J* = 8.4 Hz, 1H), 5.48 (dt, *J* = 10.9 Hz, 5.6 Hz, 1H), 5.13-5.08 (m, 1H), 5.08-5.02 (m, 1H), 4.20-4.06 (m, 2H), 3.72 (s, 1H), 3.23-3.05 (m, 2H), 2.27-2.14 (m, 1H), 2.08-1.96 (m, 3H), 1.93-1.76 (m, 3H), 1.71 (s, 3H), 1.67 (s, 3H), 1.62 (s, 3H), 1.61 (s, 3H), 1.40-1.29 (m, 2H), 1.27 (s, 3H), 0.96 (dd, *J* = 10.7 Hz, 6.9 Hz, 2H), 0.02 (s, 9H).

^{13}**C** NMR (100 MHz, $CDCl_3$) δ 205.6, 168.9, 133.9, 132.7, 131.4, 124.5, 123.8, 121.5, 63.0, 62.9, 44.5, 43.8, 42.7, 34.6, 28.5, 27.2, 25.9, 25.7, 24.4, 22.4, 18.0, 17.7, 17.3, -1.6.

HRMS (ESI) *m/z*: $[M + H]^+$ calcd for $C_{26}H_{45}O_3Si$ 433.3133; found 433.3129.

Epimerisation

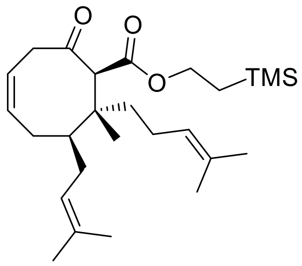

A one-neck round-bottom flask equipped with a reflux condenser was charged with 2.36 g (5.46 mmol) of the diastereomeric mixture of **11** (3.28 mmol **11a**) dissolved in THF (60 mL). To this solution was added 90 µL (0.60 mmol) DBU. The reaction mixture was put in a pre-heated oil bath at 60 °C and stirred for 20 hours. After cooling to room temperature, the reactions mixture was concentrated in vacuo. Purification of the residue by flash column chromatography (^{n}Pen/Et_2O = 30:1 → 20:1) afforded 383 mg (0.89 mmol 70% BRSM) of **11d** as a colorless oil. Alongside 1.61 g (3.73 mmol, 68%) of the diastereomeric mixture was recovered (2.01 mmol, 61% **11a**).

$\mathbf{R_f}$ 0.24 (^{n}Pen/Et_2O = 20:1).

^{1}**H** NMR (400 MHz, $CDCl_3$) δ 5.82-5.76 (m, 1H), 5.50 (ddd, *J* =10.2 Hz, 7.2 Hz, 2.9 Hz, 1H), 5.05-5.03 (m, 1H), 4.97 (tsept, *J* = 7.0 Hz, 1.3 Hz, 1H), 4.17-4.05 (m, 2H), 4.13 (s, 1H), 3.20-3.06 (m, 2H), 2.01-1.96 (m, 1H), 1.94-1.86 (m, 1H), 1.81-1.75 (m, 2H), 1.71 (s, 3H) 1.70-1.59 (m, 3H), 1.63 (s, 3H), 1.57 (s, 3H), 1.55 (s, 3H), 1.51-1.40 (m, 2H), 1.14 (s, 3H), 0.93 (t, *J* = 8.6 Hz, 2H), 0.01 (s, 9H).

^{13}**C** NMR (100 MHz, $CDCl_3$) δ 205.2, 168.3, 134.6, 133.4, 131.3, 124.1, 123.2, 120.9, 63.1, 59.5), 46.0, 44.3, 44.0, 37.5, 29.2, 27.9, 25.9, 25.6, 22.0, 17.9, 17.5, 17.3, 17.1, -1.6.

HRMS (ESI) *m/z*: $[M + H]^+$ calcd for $C_{26}H_{45}O_3Si$ 433.3133; found 433.319.

2-(Trimethylsilyl)ethyl-2-methoxy-8-methyl-7-(3-methylbut-2-en-1-yl)-8-(4-methylpent-3-en-1-yl)cycloocta-2,4-diene-1-carboxylate (14)

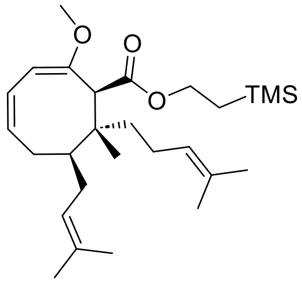

1.12 g (±)-CSA (4.82 mmol) and 1.03 g (9.71 mmol) $HC(OMe)_3$ were pre-mixed in MeOH (5 mL) until all solids dissolved. The resulting solution was added to a one-neck round-bottom flask equipped with a reflux condenser containing 2.11 g (4.88 mmol) of **11d** in MeOH (45 mL). The reaction flask was put in a pre-heated oil bath at 60 °C for three and a half hours. The reaction was allowed to cool to ambient temperature. Then 1 g of $NaHCO_3$ was added. The suspension was stirred until no more gas evolution was observed. The mixture was passed through a glass frit to remove any solids, and the remaining solution was concentrated in vacuo. The residue was dissolved in H_2O/Et_2O (1:1). The layers were separated, and the aqueous layer was extracted thrice with Et_2O. The combined organic extracts were dried over anhydrous Na_2SO_4 and concentrated in vacuo. Purification of the residue by flash column chromatography (^{n}Pen/Et_2O = 30:1 → 20:1) afforded 948 mg (2.12 mmol, 63% BRSM) of **14** as a colorless oil that crystallized in the cold during long time storage. Additionally, 656 mg (1.52 mmol, 31%) of the starting material was recovered.

R*f* 0.35 (^{n}Pen/Et_2O = 20:1).

^{1}H NMR (400 MHz, $CDCl_3$) δ 5.99-5.94 (m, 1H), 5.74-5.63 (m, 1H), 5.13-5.07 (m, 1H), 5.11 (d, *J* = 3.4 Hz, 1H), 5.00-4.97 (m, 1H), 4.11 (t, *J* = 8.4 Hz, 2H), 3.56 (s, 3H), 3.40 (s, 1H), 2.15-2.06 (m, 1H), 2.04-1.93 (m, 3H), 1.91-1.83 (m, 1H), 1.77-1.47 (m, 3H), 1.73 (s, 3H), 1.64 (s, 3H), 1.58 (s, 3H), 1.53 (s, 3H), 1.29-1.23 (m, 1H), 1.00 (s, 3H), 0.99-0.85 (m, 2H), 0.01 (s, 9H).

^{13}C NMR (100 MHz, $CDCl_3$) δ 170.8, 155.6, 132.7, 131.9, 130.8, 125.4, 124.7, 124.3, 98.3, 62.2, 55.0, 53.4, 41.2, 37.0, 35.8, 39.9, 28.2, 25.9, 25.7, 22.1, 18.0, 17.9, 17.5, 17.1, -1.5.

HRMS (ESI) *m/z*: $[M + H]^+$ calcd for $C_{27}H_{47}O_3Si$ 447.3289; found 447.3291.

2-Methoxy-8-methyl-7-(3-methylbut-2-en-1-yl)-8-(4-methylpent-3-en-1-yl)cyclo-octa-2,4-diene-1-carboxylic acid (3)

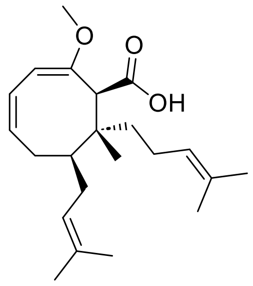

In a one-neck round-bottom flask equipped with a reflux condenser 273 mg (0.53 mmol) of enol ether **14** in THF (3 mL) was treated with 2 mL (2.00 mmol, 1 M in THF) TBAF and stirred for two and a half hours at 40 °C. The reaction was allowed to cool to ambient temperature before diluting with H_2O. The layers were separated, and the aqueous layer was extracted thrice with Et_2O. The combined organic extracts were dried over anhydrous Na_2SO_4 and concentrated in vacuo. Purification of the residue by flash column chromatography (^{n}Pen/Et_2O = 2:1) afforded 179 mg (0.52 mmol, 98%) **3** as colorless crystals.

R*f* 0.18 (^{n}Pen/Et_2O = 2:1).

^{1}H NMR (400 MHz, $CDCl_3$) δ 6.00 (d, *J* = 8.2 Hz, 1H), 5.69 (q, *J* = 8.2 Hz, 1H), 5.18 (d, *J* = 3.8 Hz, 1H), 5.09 (tsept, *J* = 5.5 Hz, 1.3 Hz, 1H), 5.03-4.98 (m, 1H), 3.61 (s, 3H), 3.52 (s, 1H), 2.12-2.06 (m, 1H), 2.04-1.95 (m, 2H), 1.93-1.85 (m, 2H), 1.77-1.51 (m, 3H), 1.73 (s, 3H), 1.63 (s, 3H), 1.59 (s, 3H), 1.54 (s, 3H), 1.31-1.23 (m, 1H), 0.98 (s, 3H).

^{13}C NMR (100 MHz, $CDCl_3$) δ 174.9, 154.6, 132.9, 132.2, 131.1, 125.3, 124.5, 124.1, 98.9, 55.1, 53.4, 41.5 ,37.4, 35.7, 29.8, 28.1, 25.9, 25.7, 22.2, 18.0, 17.9, 17.5.

HRMS (ESI) *m/z*: $[M - H]^-$ calcd for $C_{22}H_{33}O_3$ 345.2435; found 345.2428.

4-Hydroxy-2-methoxy-8-methyl-7-(3-methylbut-2-en-1-yl)-8-(4-methylpent-3-en-1-yl)bicyclo[3.3.1]non-2-en-9-one (15)

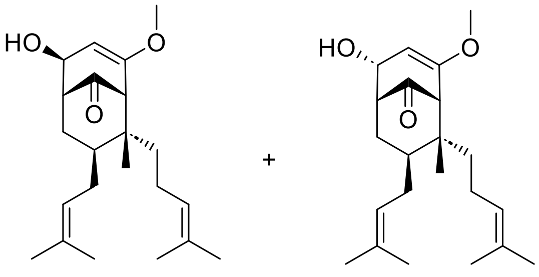

major (*exo*) **minor (*endo*)**

$CHCl_3$ stabilized with EtOH was passed through a pad of basic Al_2O_3 (activity I) prior to use. All steps involving vacuum were conducted with care as **15** is prone to sublimation.

In a one-neck round-bottom flask 101 mg (0.29 mmol) of carboxylic acid **3** and 610 mg (2.96 mmol) 2,6-di-tert-butyl-4-methylpyridine were dissolved in $CHCl_3$ (3 mL) and cooled to –40 °C. 56 µL (0.40 mmol) freshly distilled TFAA was added. After 30 minutes the reaction is warmed to room temperature. All volatiles were removed in vacuo. The residue was treated with 3 mL of saturated aq. K_2CO_3 and stirred vigorously. After one hour the mixture was diluted with saturated aq. NaCl. Et_2O was added and the layers were separated. The aqueous layer was extracted three times with Et_2O. The combined organic extracts were dried over anhydrous Na_2SO_4 and carefully concentrated in vacuo. Purification of the residue by flash column chromatography (^{n}Pen/Et_2O = 3:1) afforded 70.1 mg (0.20 mmol, 69%, 1.4:1 dr) of **15** as a colorless solid and mixture of two diastereomers.

***exo*-15 (major)**

R_f 0.22 (^{n}Pen/Et_2O = 3:1).

^{1}H NMR (500 MHz, $CDCl_3$) δ 5.07-4.99, (m, 2H), 4.95 (d, *J* = 4.1 Hz, 1H), 4.23 (d, *J* = 4.1 Hz, 1H), 3.55 (s, 3H), 2.70 (s, 1H), 2.55-2.53 (m, 1H), 2.22-2.08 (m, 3H), 1.93-1.88 (m, 1H), 1.82-1.73 (m, 1H), 1.69 (s, 3H), 1.66 (s, 3H), 1.64-1.61 (m, 1H), 1.60 (s, 3H), 1.56 (s, 3H), 1.53-1.49 (m, 1H), 1.43 (td, *J* = 12.8 Hz, 4.3 Hz, 1H), 1.21.1.14 (m, 1H), 0.78 (s, 3H).

^{13}C NMR (125 MHz, $CDCl_3$) δ 211.5, 156.2, 132.7, 131.1, 124.7, 123.1, 97.8, 72.2, 58.7, 54.7, 51.7, 44.5, 39.1, 38.4, 34.1, 28.2, 25.8, 25.7, 21.7, 17.9, 17.5.

HRMS (ESI) *m/z*: $[M + H]^+$ calcd for $C_{22}H_{35}O_3$ 347.2581; found 347.2580.

***endo*-15 (minor)**

R_f 0.22 (^{n}Pen/Et_2O = 3:1).

^{1}H NMR (500 MHz, $CDCl_3$) δ 5.07-4.99, (m, 2H), 4.93 (d, *J* = 4.1 Hz, 1H), 4.07 (d, *J* = 4.1 Hz, 1H), 3.54 (s, 3H), 2.73 (s, 1H), 2.55-2.53 (m, 1H), 2.22-2.08 (m, 3H), 1.93-1.88 (m, 1H), 1.82-1.73 (m, 1H), 1.71 (s, 3H), 1.66 (s, 3H), 1.64-1.61 (m, 1H), 1.59 (s, 3H), 1.57 (s, 3H), 1.53-1.49 (m, 1H), 1.43 (td, *J* = 12.8 Hz, 4.3 Hz, 1H), 1.21.1.14 (m, 1H), 0.79 (s, 3H).

^{13}C NMR (125 MHz, $CDCl_3$) δ 211.2, 156.3, 132.6, 131.2, 124.6, 123.2, 98.2, 78.8, 58.6, 54.9, 50.9, 44.1, 39.2, 38.5, 34.2, 28.3, 25.9, 25.7, 21.7, 17.9, 17.5.

HRMS (ESI) *m/z*: $[M + H]^+$ calcd for $C_{22}H_{35}O_3$ 347.2581; found 347.2580.

4-Methoxy-6-methyl-7-(3-methylbut-2-en-1-yl)-6-(4-methylpent-3-en-1-yl)bicyclo[3.3.1]non-3-ene-2,9-dione (16)

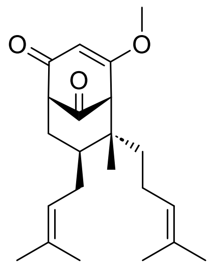

In a one-neck round-bottom flask 53.3 mg (0.15 mmol) of the diastereomeric mixture of alcohol **15** was dissolved in DCM (2.5 mL) and cooled to 0° C. 69.1 mg (0.32 mmol) fine powdered PCC and 27.6 mg (0.34 mmol) NaOAc were mixed and added to the reaction mixture. After 30 minutes the reaction was allowed to attain room temperature and stirred for an additional 40 minutes. The brown suspension was filtered through a small pad of silica gel. The pad was washed extensively with DCM and then Et_2O. The solvent was removed in vacuo. Purification of the residue by flash column chromatography (^{n}Pen/Et_2O = 3:1 → 1:1) afforded 38.9 mg (0.11 mmol, 73%) of **16** as colorless crystals.

R_f 0.11 (^{n}Pen/Et_2O = 3:1).

R_f 0.30 (^{n}Pen/Et_2O = 1:1).

^{1}H NMR (500 MHz, $CDCl_3$) δ 5.73 (s, 1H), 5.02 (tsept, *J* = 7.3 Hz, 1.3 HZ, 1H), 4.97-4.94 (m, 1H), 3.76 (s, 3H), 3.16-3.14 (m, 1H), 3.10 (s, 1H), 2.23-2.16 (m, 2H), 2.13-2.08 (m, 1H), 1.89-1.80 (m, 1H), 1.69 (s, 3H), 1.67.1.54 (m, 4H), 1.64 (s, 3H), 1.62 (s, 3H), 1.54 (s, 3H), 1.12 (td, *J* = 13.1 Hz, 4.1 Hz, 1H), 0.91 (s, 3H).

^{13}C NMR (125 MHz, $CDCl_3$) δ 206.5, 195.7, 175.6, 133.4, 131.7, 124.0, 122.1, 105.9, 61.4, 61.0, 56.7, 44.5, 38.9, 38.5, 33.5, 28.0, 25.8, 25.7, 21.6, 17.9, 17.6, 17.5.

HRMS (ESI) *m/z*: $[M + H]^+$ calcd for $C_{22}H_{33}O_3$ 345.2424; found 345.2407.

4-Methoxy-8-methyl-7-(3-methylbut-2-en-1-yl)-8-(4-methylpent-3-en-1-yl)bicyclo[3.3.1]non-3-ene-2,9-dione (17)

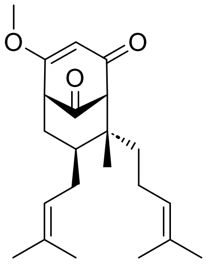

To a one-neck round-bottom flask containing 107 mg (0.31 mmol) of ketone **16** in MeOH (6 mL) were added 0.66 mL (15.5 mmol) $HC(OMe)_3$ and 12.5 mg (65.7 µmol) pTsOH·H_2O. The mixture was heated to 50 °C and stirred for 42 to 50 hours.[5] The pale violet solution was allowed to reach room temperature. All volatiles were removed in vacuo and the residue was dissolved in THF (3 mL). 6 mL (6 mmol) HCl was added, and the mixture was heated to 50 °C. After stirring vigorously for 25 minutes the reaction was neutralized with saturated aq. K_2CO_3 solution. The layers were separated, and the aqueous layer was extracted thrice with

[5] Progression of the reaction was monitored via NMR.

Et_2O. The combined organic extracts were dried over anhydrous $MgSO_4$ and concentrated in vacuo. Purification of the residue by flash column chromatography (^{n}Pen/Et_2O = 3:1 → 1:1) afforded 74.4 mg (0.22 mmol, 70%) of **17** as pale-yellow solid. Alongside 13.9 mg (40.2 µmol, 13%) of starting material **16** was recovered.

R*f* 0.23 (^{n}Pen/Et_2O = 3:1).

R*f* 0.41 (^{n}Pen/Et_2O = 1:1).

^{1}H NMR (400 MHz, $CDCl_3$) δ 5.67 (s, 1H), 5.08 (tsept, *J* = 7.2 Hz, 1.2 Hz, 1H), 4.95-4.93 (m, 1H), 3.76 (s, 3H), 3.13 (bs, 1H), 3.09 (s, 1H), 2.42 (tt, *J* = 12.5 Hz, 6.1 Hz, 1H), 2.18-2.14 (m, 1H), 2.07 (dd, *J* = 10.0 Hz, 2.9 Hz, 1H), 1.84 (tt, *J* = 12.4 Hz, 6.1 Hz, 1H), 1.75-1.61 (m, 3H), 1.67 (s, 9H), 1.55 (s, 3H), 1.50 (dd, *J* = 12.4 Hz, 4.0 Hz, 1H), 1.30 (td, *J* = 13.0 Hz, 4.8 Hz, 1H), 0.87 (s, 3H).

^{13}C NMR (100 MHz, $CDCl_3$) δ 206.6, 194.1, 175.5, 133.2, 131.8, 124.2, 122.3, 105.4, 69.4, 56.9, 53.0, 45.7, 39.6, 38.5, 32.0, 27.5, 25.8, 25.7, 21.6, 17.9, 17.6, 17.5.

HRMS (ESI) *m/z*: $[M + H]^+$ calcd for $C_{22}H_{33}O_3$ 345.2424; found 345.2450.

4-Methoxy-8-methyl-5,7-bis(3-methylbut-2-en-1-yl)-8-(4-methylpent-3-en-1-yl)bi-cyclo[3.3.1]non-3-ene-2,9-dione (18)

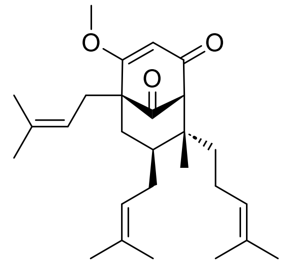

Cy_2NLi was prepared as follows: In a Schlenk flask 0.20 mL (1.00 mmol) Cy_2NH was dissolved in THF (2 mL) and cooled to –78 °C. 0.40 mL (1.00 mmol, 2.5 M in ^{n}Hex) ^{n}BuLi was added dropwise. The reaction was stirred at –78 °C for 30 minutes. The cloudy mixture was warmed to –40 °C until a clear solution was obtained.

In a one-neck round-bottom flask 86.2 mg (0.25 mmol) of ketone **17** was dissolved in THF (5 mL) and cooled to –78 °C. 1.1 mL (0.55 mmol, 0.5 M in THF) Cy_2NLi was added dropwise to the solution. The reaction mixture turned from yellow to orange during addition. After 30 minutes 0.29 mL (2.51 mmol) prenyl bromide was added rapidly in one portion. The reaction mixture brightened, turning pale-yellow or colorless shortly thereafter. After 15 minutes the cooling bath was removed, and the reaction was terminated with H_2O. The layers were separated, and the aqueous layer was extracted thrice with Et_2O. The combined organic extracts were dried over anhydrous $MgSO_4$ and concentrated in vacuo. Purification of the residue by flash column chromatography (^{n}Pen/Et_2O = 10:1 → 3:1) afforded 69.2 mg (0.18 mmol, 73%) **18** as a pale-yellow oil.

R*f* 0.18 (^{n}Pen/Et_2O = 10:1).

R*f* 0.39 (^{n}Pen/Et_2O = 3:1).

^{1}H NMR (400 MHz, $CDCl_3$) δ 5.71 (s, 1H), 5.07 (tsept, *J* = 7.2 Hz, 1.2 Hz, 1H), 4.97-4.94 (m, 2H), 3.74 (s, 3H), 3.13 (s, 1H), 2.47-2.31 (m, 3H), 2.17-2.11 (m, 1H), 1.93 (dd, *J* = 13.8 Hz, 3.8 Hz, 1H), 1.87-1.80 (m, 1H), 1.73-1.63 (m, 2H), 1.68 (s, 3H), 1.66 (s, 6H), 1.64 (s, 3H), 1.63 (s, 3H), 1.55 (s, 3H), 1.49 (dd, *J* = 12.6 Hz, 4.2 Hz, 1H), 1.43-1.37 (m, 1H), 1.25 (td, *J* = 13.4 Hz, 4.6 Hz, 1H), 0.84 (s, 3H).

^{13}C NMR (100 MHz, $CDCl_3$) δ 207.0, 193.8, 177.4, 133.6, 133.1, 131.6, 124.3, 122.5, 119.4, 106.2, 70.4, 56.8, 56.7, 45.9, 40.7, 39.2, 38.5, 29.6, 27.6, 25.9, 25.8, 25.7, 21.7, 17.9, 17.8, 17.7, 17.6.

HRMS (ESI) *m/z*: $[M + H]^+$ calcd for $C_{27}H_{41}O_3$ 413.3050; found 413.3059.

Hyperforin (1) was prepared from **18** according to literature with all NMR data in accordance with the described compounds.[11]

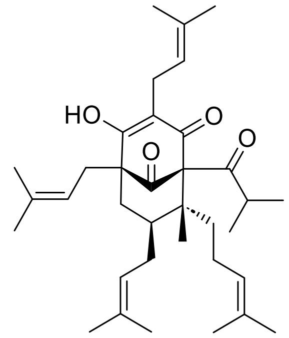

^{1}H NMR (500 MHz, MeOD) δ 5.10 (t, *J* = 7.1 Hz, 1H), 5.03-4.95 (m, 3H), 3.14 (dd, *J* = 14.7 Hz, 7.2 Hz, 1H), 3.08 (dd, *J* = 14.8 Hz, 6.9 Hz, 1H), 2.50 (dd, *J* = 14.6 Hz, 6.7 Hz, 1H), 2.41 (dd, *J* = 14.7 Hz, 6.9 Hz, 1H), 2.14-2.10 (m, 1H), 2.09-2.04 (m, 1H), 1.99-1.95 (m, 1H), 1.93-1.87 (m, 2H), 1.78-1.67 (m, 4H), 1.70 (s, 3H), 1.68 (s, 6H), 1.65 (s, 3H), 1.64 (s, 3H), 1.63 (s, 3H), 1.59 (s, 3H), 1.58 (s, 3H), 1.39 (t, *J* = 12.9 Hz, 1H), 1.09 (d, *J* = 6.6 Hz, 3H), 1.03 (d, *J* = 6.6 Hz, 3H), 0.97 (s, 3H).

^{13}C NMR (125 MHz, MeOD) δ 212.0, 209.1, 134.8, 134.4, 133.6, 132.0, 126.2, 124.0, 122.8, 122.2, 121.1, 82.8, 60.9, 49.8, 43.2, 41.0, 38.1, 30.9, 28.8, 26.3, 26.2, 26.1, 26.0, 25.6, 22.7, 22.1, 21.3, 18.4, 18.3, 18.2, 18.0, 15.4.

HRMS (ESI) *m/z*: $[M - H]^-$ calcd for $C_{35}H_{51}O_4$ 535.3793; found 535.3778.

Comparison of natural and synthetic Hyperforin (1)

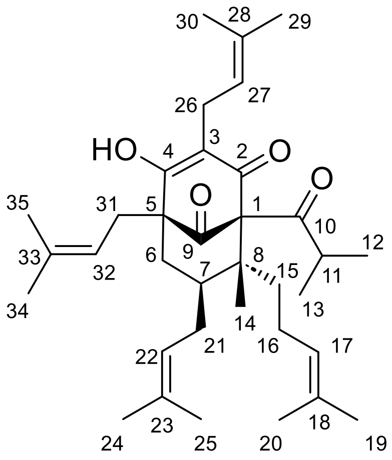

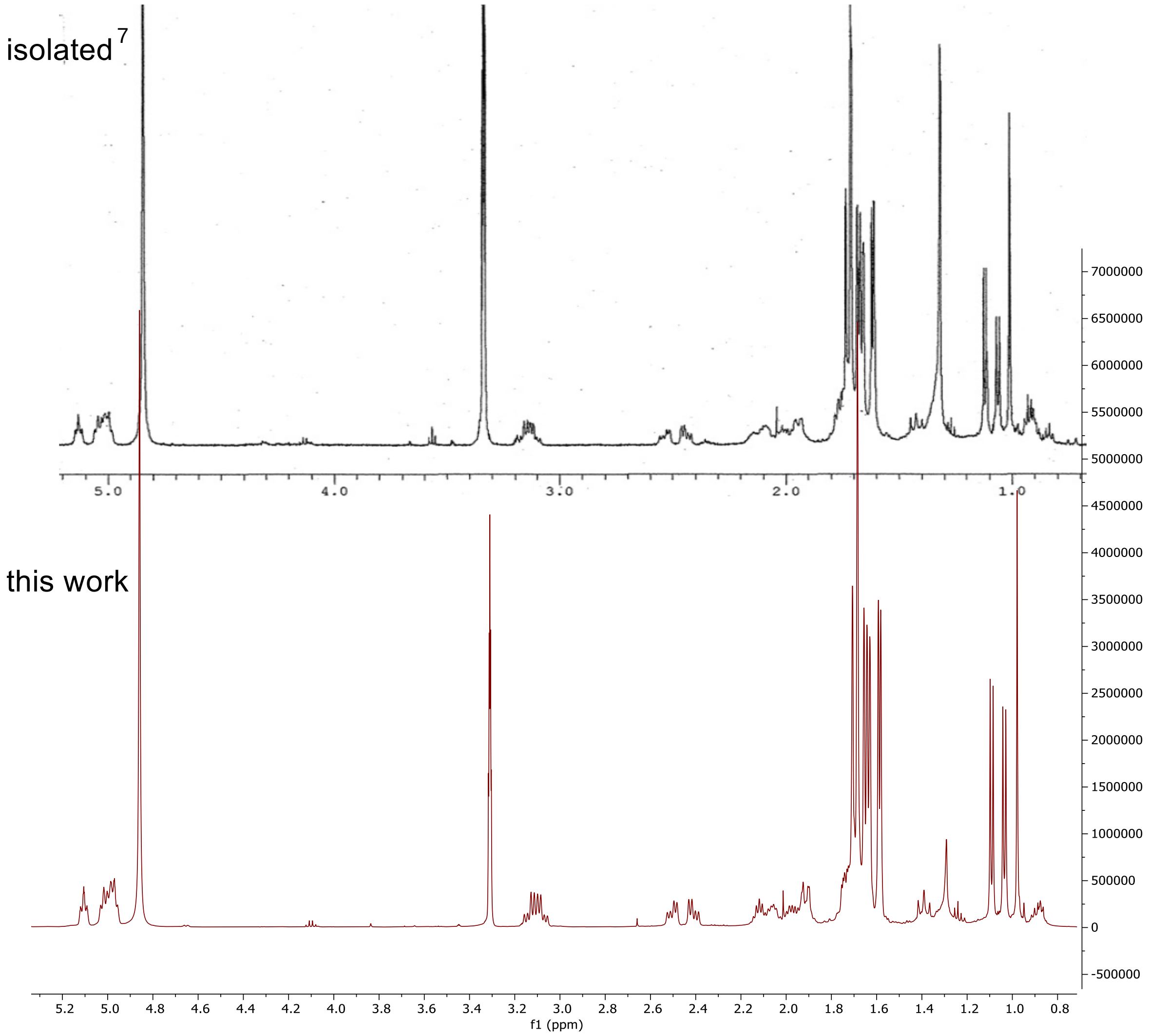

Table 1: ^{1}H-NMR comparison of natural and synthetic Hyperforin (**1**). MeOD was used as a solvent.

	500 MHz[6]	500 MHz[7]	500 MHz[8]	600 MHz[9]	500 MHz[10]	600 MHz[11]	600 MHz[12]	500 MHz this w ork
27	5.00 (m, 1H)	5.15-5.11 (m, 1H)	5.12 (t, *J* = 7.0, 1H)	5.10-5.00 (m, 1H)	5.09 (tt, *J* = 7.1, 1.3, 1H)	5.12 (t, *J* = 6.4, 1H)	5.10 (t, *J* = 7.0, 1H)	5.10 (t, *J* = 7.1, 1H)
22	4.90 (m, 1H)	5.07-4.97 (m, 3H)	5.04-4.95 (m, 3H)	5.00-4.88 (m, 3H)	5.03-4.94 (m, 3H)	5.04-4.96 (m, 3H)	5.04-4.94 (m, 3H)	5.03-4.95 [4.99] (m, 1H)
32	4.87 (m, 1H)							5.03-4.95 [4.97] (m, 1H)
17	4.92 (m, 1H)							5.03-4.95 [5.01] (m, 1H)
26a	3.05 (dd, 1H)	3.17 (dd, *J* = 14.7, 7.0, 1H)	3.12 (dd, *J* = 14.6, 7.2, 1H)	3.10 (dd, *J* = 14.4, 7.2, 1H)	3.13 (dd, *J* = 14.9, 6.9, 1H)	3.16 (dd, *J* = 14.8, 7.2, 1H)	3.13 (dd, *J* = 14.7, 7.2, 1H)	3.14 (dd, *J* = 14.7, 7.2, 1H)
26b	2.99 dd (na)	3.11 (dd, *J* = 14.7, 6.7, 1H)	3.07 (dd, *J* = 14.7, 7.1, 1H)	3.05 (dd, *J* = 14.4, 7.2, 1H)	3.07 (dd, *J* = 14.9, 6.9, 1H)	3.10 (dd, *J* = 14.8, 6.9, 1H)	3.08 (dd, *J* = 14.7, 6.9, 1H)	3.08 (dd, *J* = 14.8, 6.9, 1H)
31a	2.41 (dd, 1H)	2.54 (dd, *J* = 14.1, 6.7, 1H)	2.49 (dd, *J* = 14.4, 6.9, 1H)	2.47 (dd, *J* = 14.4, 6.6, 1H)	2.50 (dd, *J* = 14.6, 6.9, 1H)	2.52 (dd, *J* = 14.9, 6.8, 1H)	2.50 (dd, *J* = 14.5, 6.6, 1H)	2.50 (dd, *J* = 14.6, 6.7, 1H)
31b	2.32 (dd, 1H)	2.44 (dd, *J* = 14.1, 7.0, 1H)	2.40 (dd, *J* = 14.6, 6.8, 1H)	2.37 (dd, *J* = 14.4, 6.6, 1H)	2.41 (dd, *J* = 14.6, 7.1, 1H)	2.43 (dd, *J* = 14.6, 7.1, 1H)	2.41 (dd, *J* = 14.6, 7.0, 1H)	2.41 (dd, *J* = 14.7, 6.9, 1H)
11	2.02 (m, 1H)	2.19-1.90 (m, 5H)	2.14 (sept, *J* =6.5, 1H)	2.20-2.11 (m, 2H)	2.11-2.02 (m, 2H)	2.14-2.12 (m, 1H)	2.17-2.02 (m, 2H)	2.14-2.10 (m, 1H)
16a	1.85 (m, 1H)		2.10-2.02 (m, 1H)			2.11-2.04 (m, 1H)		1.99-1.95 (m, 1H)
6a	1.82 (m, 1H)		2.02-1.87 (m, 3H)	2.01-1.91 (m, 1H)	2.01-1.86 (m, 3H)	2.04-1.97 (m, 1H)	2.02-1.86 (m, 3H)	1.93-1.87 (m, 1H)
21a	1.95 (m, 1H)			1.97 (dd, *J* = 13.2, 4.8, 1H)		1.97-1.89 (m, 2H)		2.09-2.04 (m, 1H)
21b	1.65 (m, 1H)			1.88 (dd, *J* = 13.2, 4.2, 1H)				1.78-1.67 (m, 3H)
7	1.64 (m, 1H)	1.79-1.66 (m, 4H)	1.78-1.72 (m, 3H)	1.75-1.62 (m, 4H)	1.77-1.70 (m, 3H)	1.80-1.74 (m, 4H)	1.78-1.70 (m, 3H)	
15a	1.59 (m, 3H)							

16b								1.93-1.87 (m, 1H)
15b			1.66-1.63 (m, 1H)		1.68-1.63 (m, 1H)		1.70-1.67 (m, 1H)	1.78-1.67 (m, 1H)
6b	1.30 (m, 1H)	1.47-1.38 (m, 1H)	1.37 (dd, *J* =13.3, 12.2,1H)	1.42-1.34 (m, 1H)	1.39 (dd, *J* = 13.3, 12.2, 1H)	1.41 (t, *J* = 12.7, 1H)	1.42-1.36 (m, 1H)	1.39 (t, *J* = 12.9, 1H)
30	1.61 (s, 3H)	1.74 (s, 3H)	1.71 (s, 3H)	1.69 (s, 3H)	1.70 (s, 3H)	1.73 (s, 3H)	1.71 (s, 3H)	1.70 (s, 3H)
19	1.56 (na)	1.72 (s, 6H)	1.68 (s, 6H)	1.67 (s, 6H)	1.68 (s, 6H)	1.71 (s, 6H)	1.68 (s, 6H)	1.65 (s, 3H)
34	1.53 (na)							1.63 (s, 3H)
35	1.59 (s, 3H)	1.69 (s, 3H)	1.66 (s, 3H)	1.64 (s, 3H)	1.65 (s, 3H)	1.68 (s, 3H)	1.66 (s, 3H)	1.68 (s, 6H)
24	1.59 (na)	1.68 (s, 3H)	1.64 (s, 3H)	1.62 (s, 3H)	1.64 (d, *J* = 0.8, 3H)	1.67 (s, 3H)	1.64 (s, 3H)	
29	1.55 (na)	1.66 (s, 3H)	1.63 (s, 3H)	1.62 (s, 3H)	1.62 (d, *J* = 0.8, 3H)	1.65 (s, 3H)	1.63 (s, 3H)	1.64 (s, 3H)
25	1.48 (s, 3H)	1.62 (s, 3H)	1.59 (s, 3H)	1.58 (s, 3H)	1.59 (s, 3H)	1.62 (s, 3H)	1.59 (s, 3H)	1.58 (s, 3H)
20	1.49 (s, 3H)	1.61 (s, 3H)	1.58 (s, 3H)	1.57 (s, 3H)	1.58 (s, 3H)	1.60 (s, 3H)	1.58 (s, 3H)	1.59 (s, 3H)
12	0.99 (d, 3H)	1.12 (d, *J* = 6.4, 3H)	1.09 (d, *J* = 6.5, 3H)	1.08 (d, *J* = 6.6, 3H)	1.09 (d, *J* = 6.4, 3H)	1.11 (d, *J* = 6.5, 3H)	1.09 (d, *J* = 6.5, 3H)	1.09 (d, *J* = 6.6, 3H)
13	0.94 (d, 3H)	1.06 (d, *J* = 6.4, 3H)	1.04 (d, *J* = 6.5, 3H)	1.03 d (d, *J* = 6.6, 3H)	1.03 (d, *J* = 6.6, 3H)	1.06 (d, *J* = 6.5, 3H)	1.03 (d, *J* = 6.5, 3H)	1.03 (d, *J* = 6.6, 3H)
14	0.88 (s, 3H)	1.01 (s, 3H)	0.97 (s, 3H)	0.96 (s, 3H)	0.97 (s, 3H)	1.00 (s, 3H)	0.98 (s, 3H)	0.97 (s, 3H)

Table 2: ^{13}C-NMR comparison of natural and synthetic Hyperforin (**1**). MeOD was used as a solvent.

	125 MHz[6]	125 MHz[7]	125 MHz[8]	150 MHz[9]	125 MHz[10]	150 MHz[11]	150 MHz[12]	125 MHz this work
1	82	na	82.6	na	na	na	na	82.8 *via* HMBC
2	na	na	na	na	na	na	na	na
3	122.1	121.9	122.1	121.7	122.1	122.3	122.0	122.2
4	na	na	na	na	na	na	na	na
5	60	na	60.7	na	na	na	na	60.9 *via* HMBC
6	40.8	41.7	40.8	40.7	40.8	41.1	40.8	41.0
7	42.9	43.9	43.0	42.8	43.0	43.3	43.0	43.2
8	49.1	na	49.5	na	49.5	49.9	49.6	49.8 *via* HMBC
9	208.9	na	208.8	na	208.9	209.3	209.0	209.1
10	211.8	na	211.7	na	211.8	212.2	211.9	212.0
11	43.0	43.7	43.1	43.3	43.1	43.4	43.0	43.2
12	22.0	22.9	22.0	22.2	22.0	22.3	22.0	22.1
13	21.2	22.0	21.2	21.2	21.2	21.4	21.2	21.3
14	15.3	16.1	15.3	15.3	15.3	15.6	15.3	15.4
15	38.0	38.8	37.9	38.0	37.9	38.3	37.9	38.1
16	25.4	26.3	25.4	25.6	25.5	25.8	25.5	25.6
17	126.0	122.8	126.0	126.4	126.1	126.4	126.1	126.2
18	131.8	-[a]	131.8	131.6	131.9	132.1	131.8	132.0
19	25.9	26.7	25.9	25.9	25.9	26.2	25.9	26.0
20	18.1	18.9	18.1	18.1	18.1	18.4	18.1	18.0
21	28.6	29.5	28.6	28.7	28.7	29.0	28.6	28.8
22	123.8	127.0	123.8	124.1	123.9	124.1	123.9	124.0
23	134.3	135.0	134.2	134.0	134.3	134.5	134.2	134.4
24	26.0	26.8	26.0	26.0	26.0	26.3	26.0	26.1
25	18.2	19.0	18.2	18.2	18.2	18.4	18.2	18.2
26	22.5	23.4	22.5	22.9	22.6	22.9	22.6	22.7
27	122.5	124.7	122.6	123.6	122.6	123.0	122.7	122.8
28	133.6	132.6	133.5	132.44	133.6	133.8	133.4	133.6
29	26.1	26.9	26.1	26.1	26.1	26.3	26.0	26.2
30	17.8	18.7	17.9	17.9	17.9	18.1	17.8	18.3
31	30.7	31.6	30.7	30.9	30.7	31.0	30.7	30.9
32	120.9	123.7	120.9	121.6	120.9	121.3	121.0	121.1
33	134.7	135.4	134.6	134.1	134.7	134.9	134.6	134.8
34	26.2	27.0	26.2	26.2	26.2	26.4	26.1	26.3
35	18.3	19.1	18.3	18.3	18.3	18.5	18.2	18.4

[6] P. Adam, D. Arigoni, A. Bacher, W. Eisenreich, *J. Med. Chem.* **2002**, *45*, 4786-4793.
[7] Y. Shimizu, S.-L. Shi, H. Usuda, M. Kanai, M. Shibasaki, *Tetrahedron* **2010**, *66*, 6569-6584.
[8] B. A. Sparling, D. C. Moebius, M. D. Shair, *J. Am. Chem. Soc.* **2013**, *135*, 644−647.
[9] M. Uwamori, M. Nakada, *Tetrahedron Lett.* **2013**, *54*, 2022-2025.
[10] G. Bellavance, L. Barriault, *Angew. Chem. Int. Ed.* **2014**, *53*, 6701-6704; *Angew. Chem.* **2014**, *126*, 6819–6822.
[11] C. P. Ting, T. J. Maimone, *J. Am. Chem. Soc.* **2015**, *137*, 10516-10519.
[12] Y. Ji, B. Hong, I. Franzoni, M. Wang, W. Guan, H. Jia, H. Li, *Angew. Chem. Int. Ed.* **2022**, *61*, e202116136; *Angew. Chem.* **2022**, *134*, e202116136.

The methyl groups in the prenyl side chains have been reassigned based on HMBC and 1D-NOE-experiments.

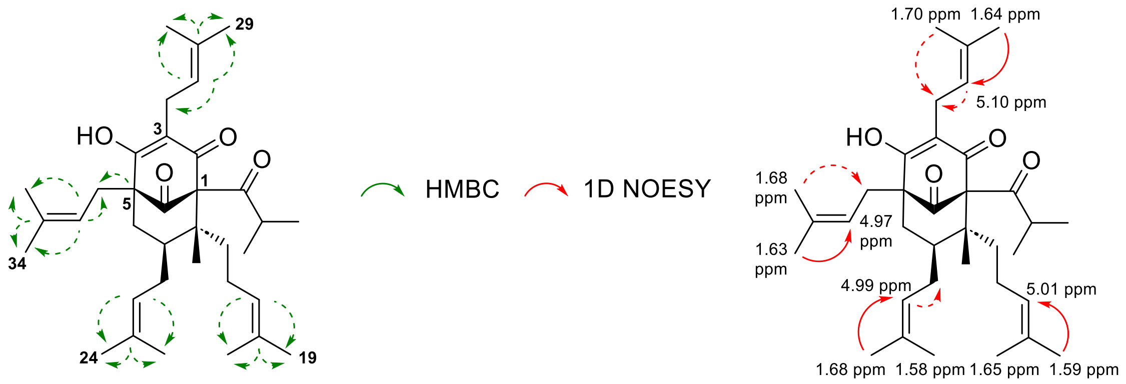

Excerpts from the spectra associated with plain arrows above are shown below.

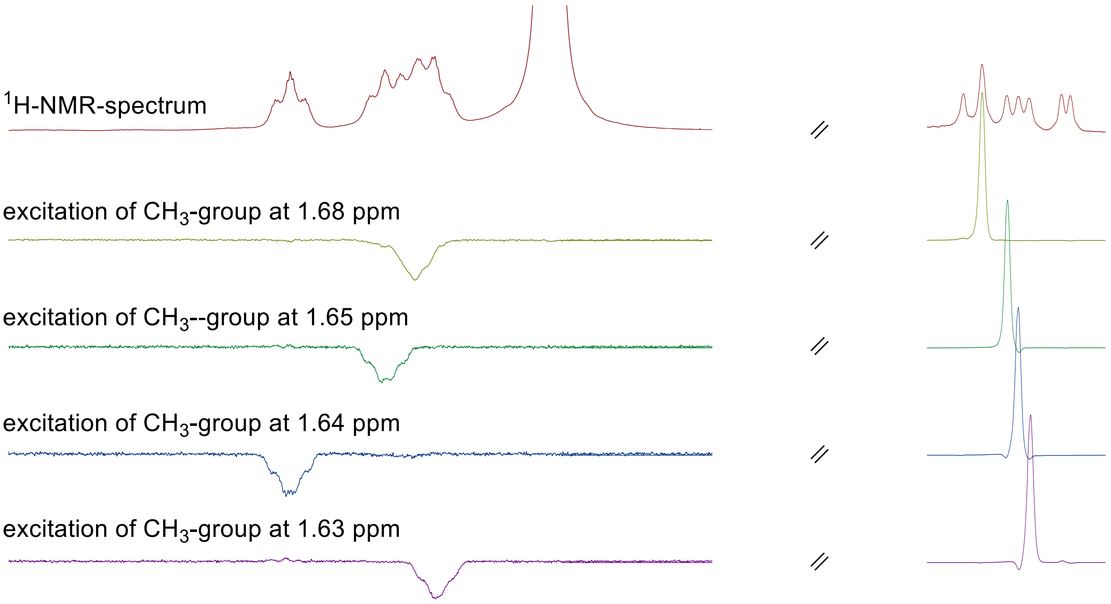

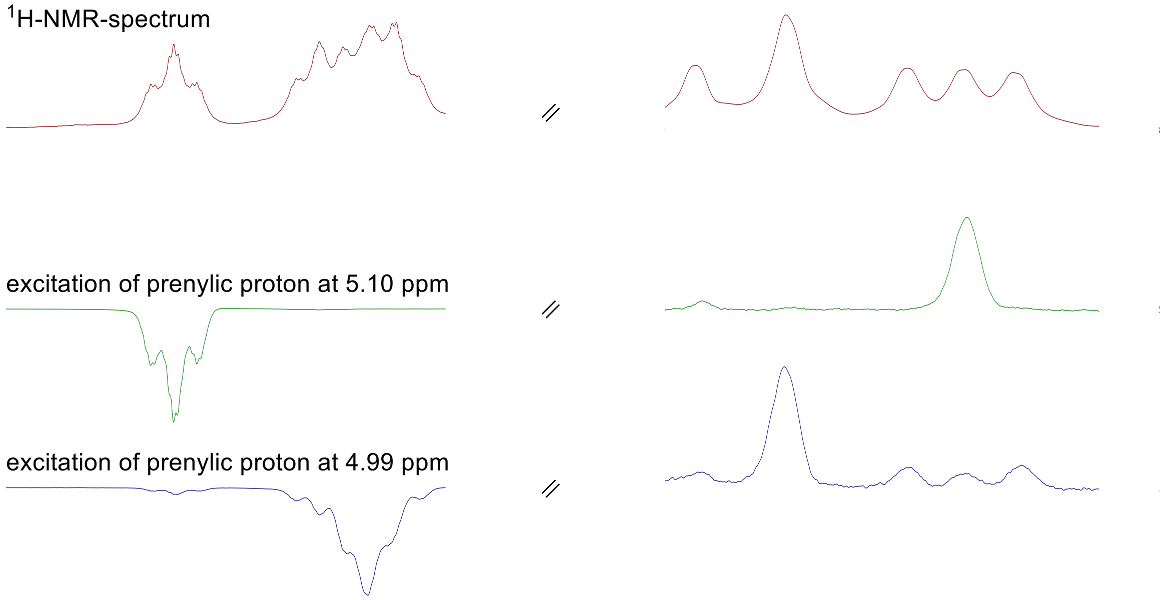

^{1}H and ^{13}C NMR spectra

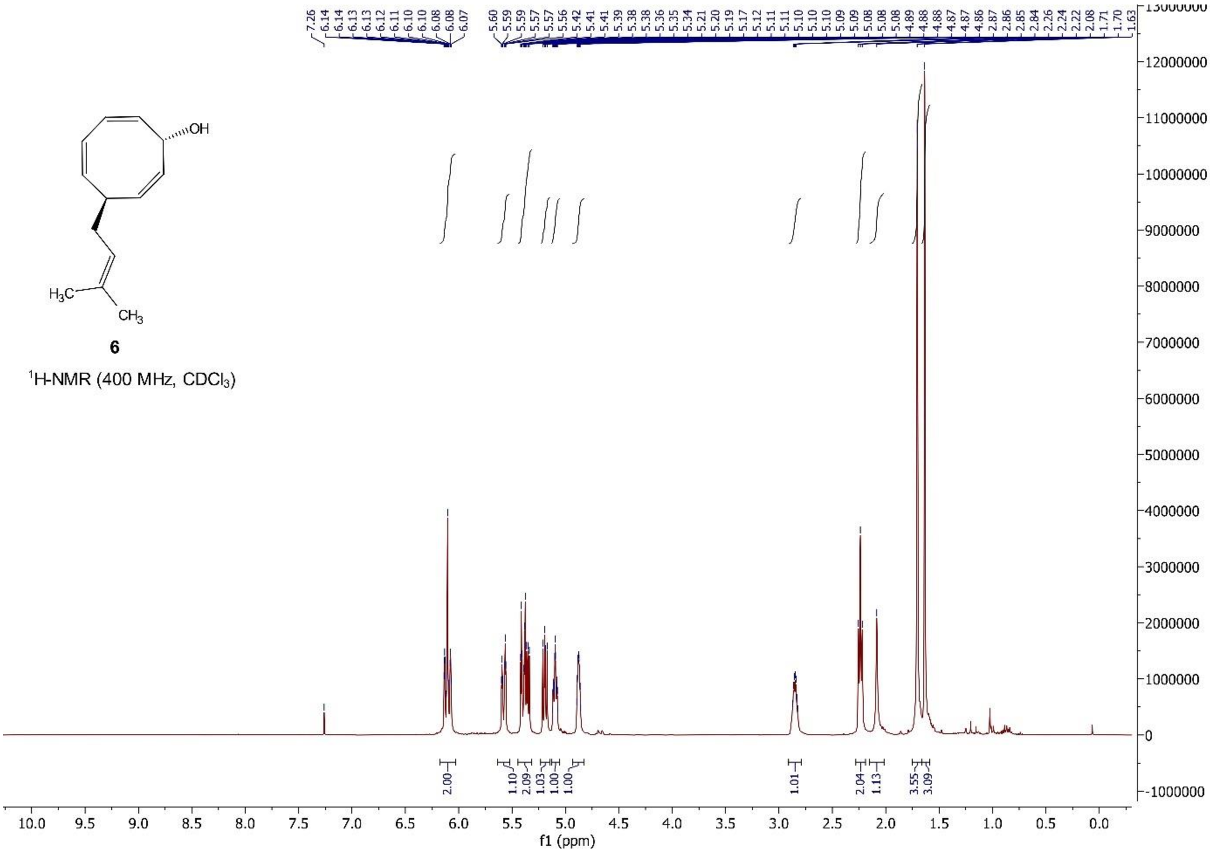

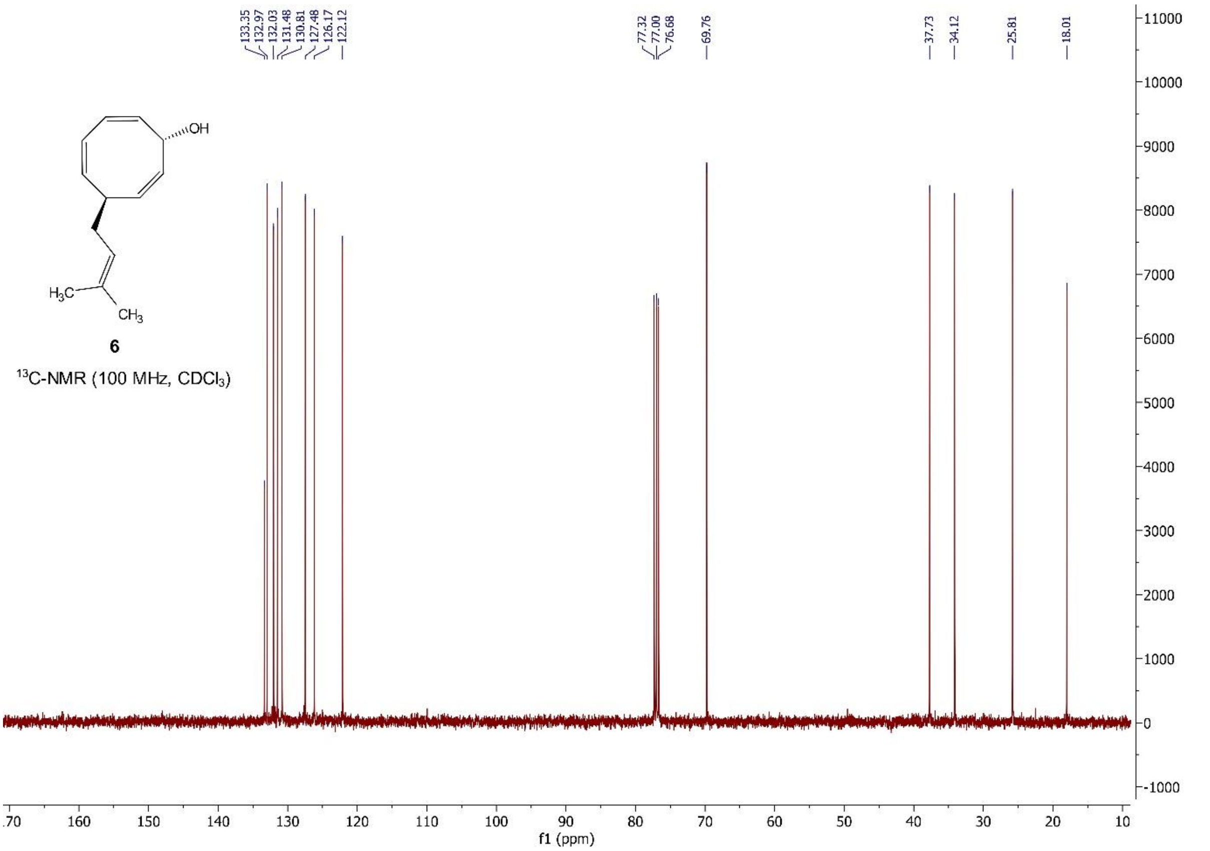

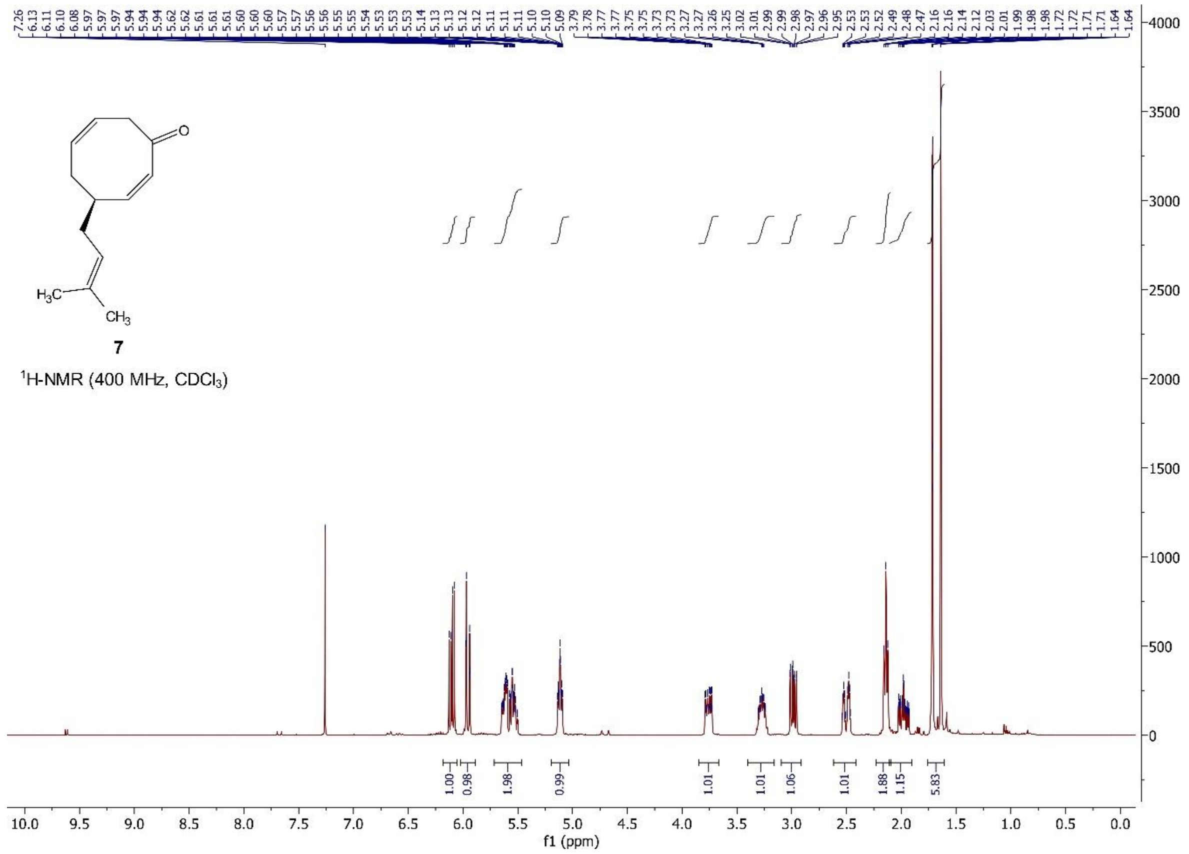

H3C
CH3
7
1H-NMR (400 MHz, CDCl3)
f1 (ppm)

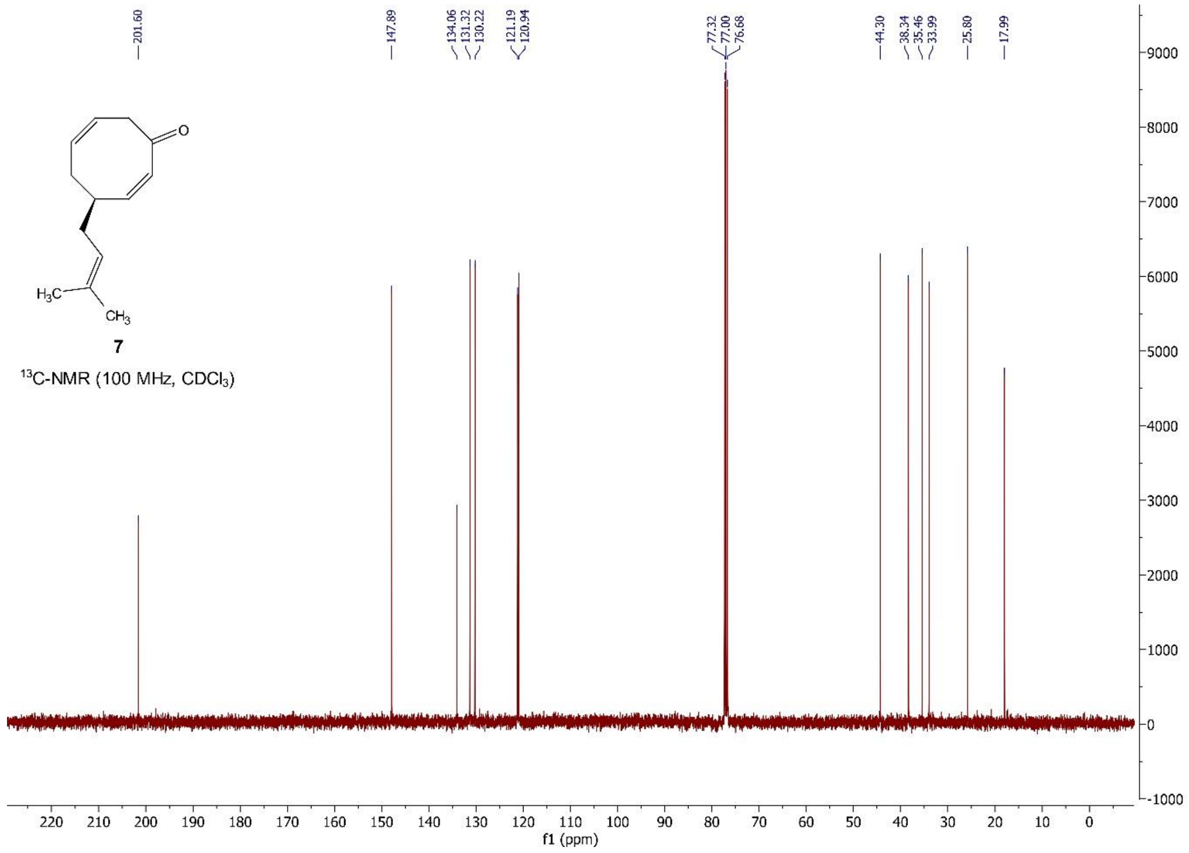

H3C
CH3
7
13C-NMR (100 MHz, CDCl3)
201.60
147.89
134.06
131.32
130.22
121.19
120.94
77.32
77.00
76.68
44.30
38.34
35.46
33.99
25.80
17.99
f1 (ppm)

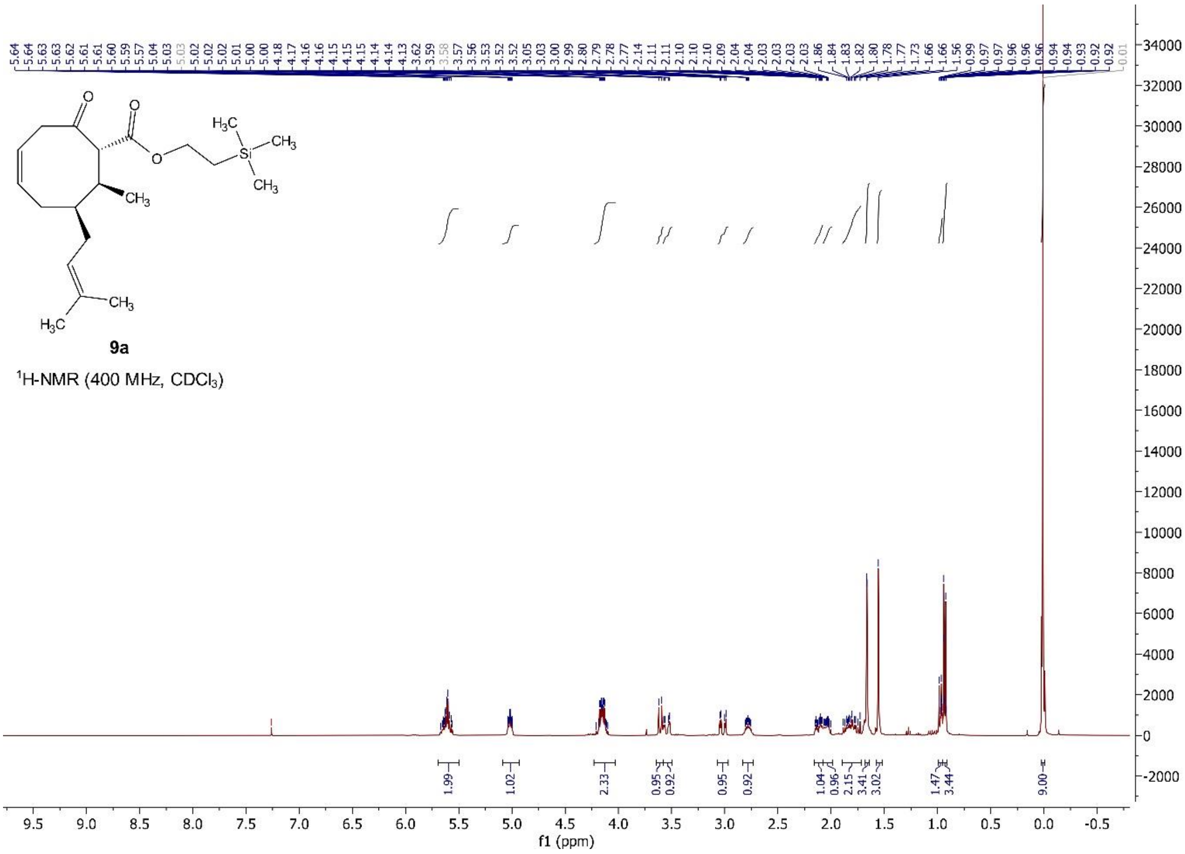
9a
1H-NMR (400 MHz, CDCl3)
f1 (ppm)

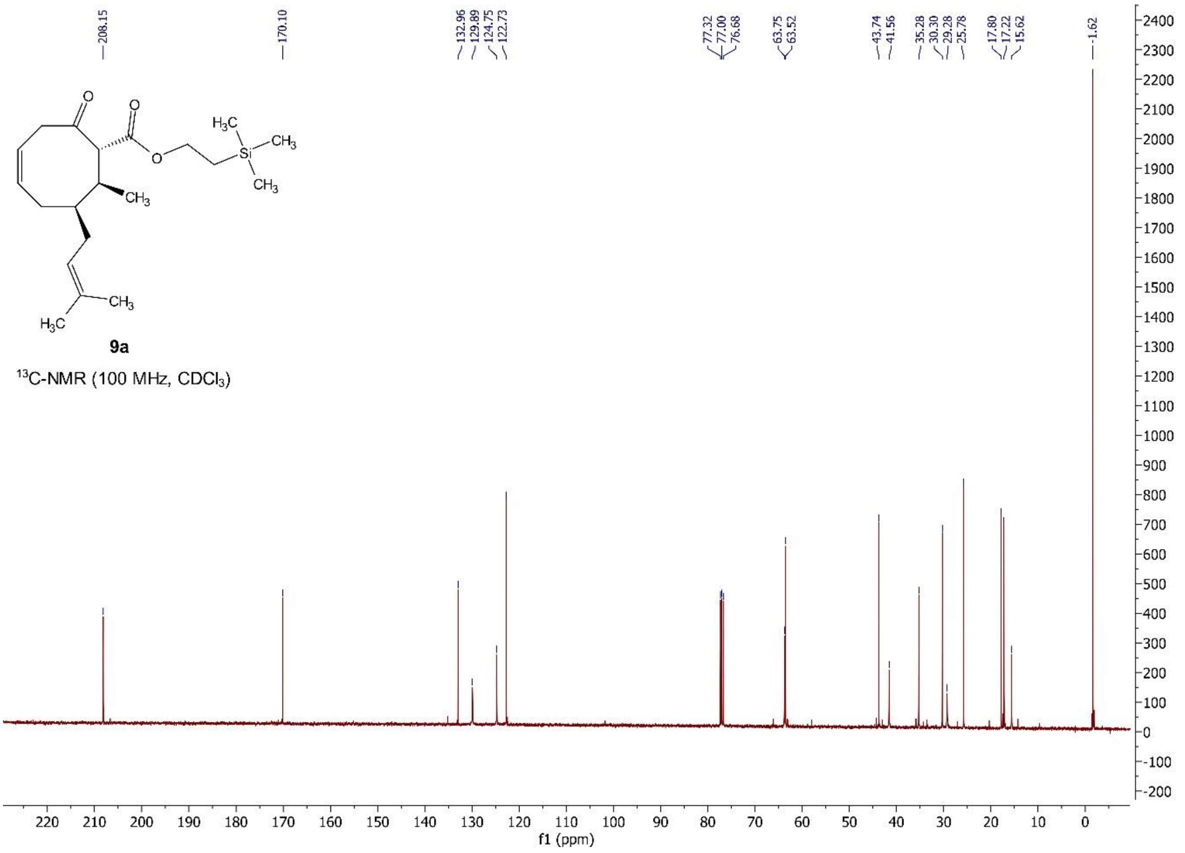
208.15
170.10
132.96
129.89
124.75
122.73
77.32
77.00
76.68
63.75
63.52
43.74
41.56
35.28
30.30
29.28
25.78
17.80
17.22
15.62
-1.62
9a
13C-NMR (100 MHz, CDCl3)
f1 (ppm)

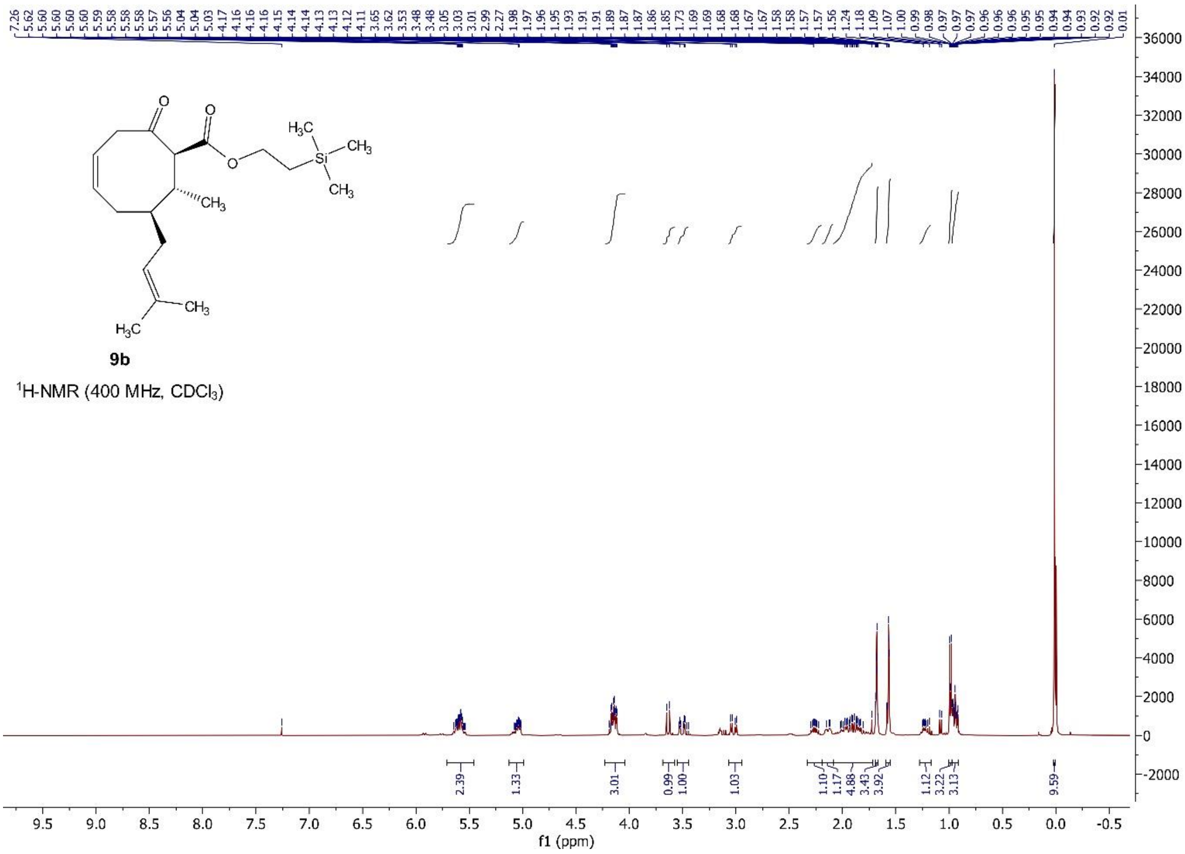
9b
^{1}H-NMR (400 MHz, $CDCl_3$)
f1 (ppm)

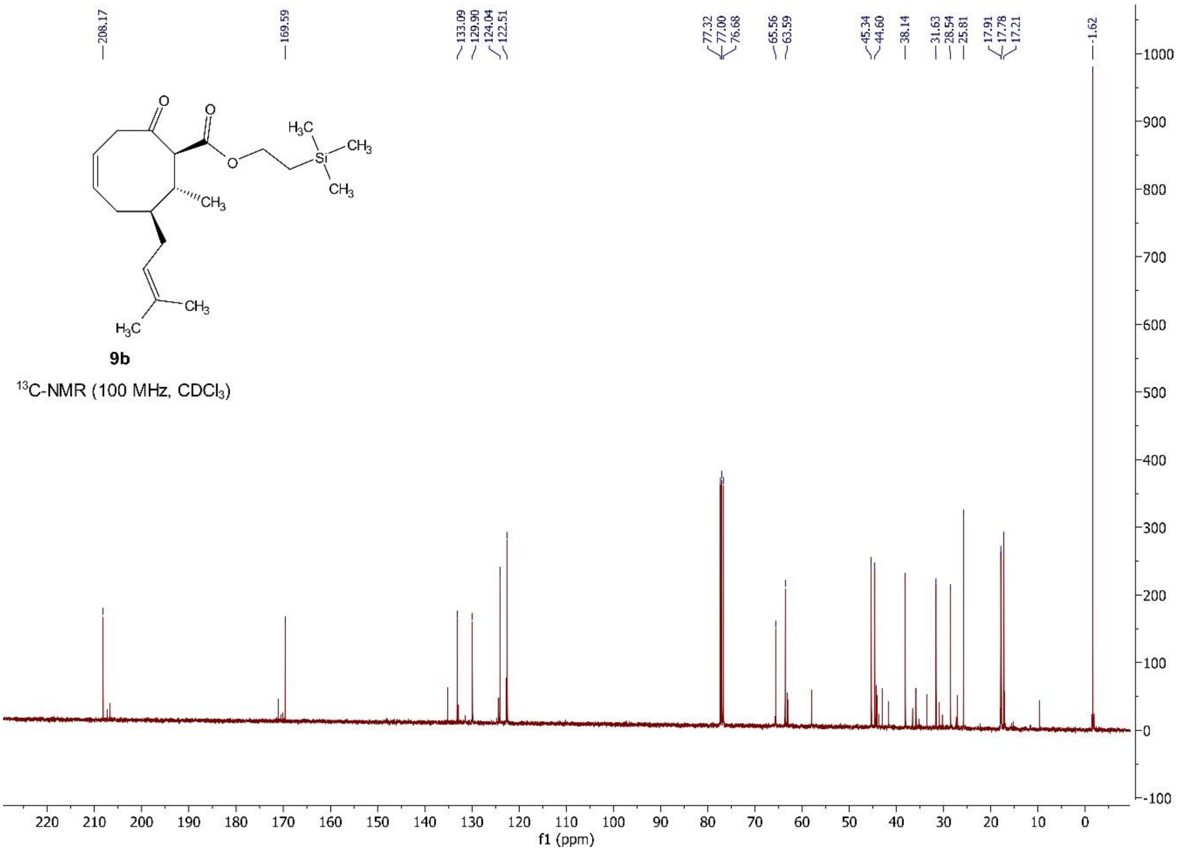
9b
^{13}C-NMR (100 MHz, $CDCl_3$)
f1 (ppm)

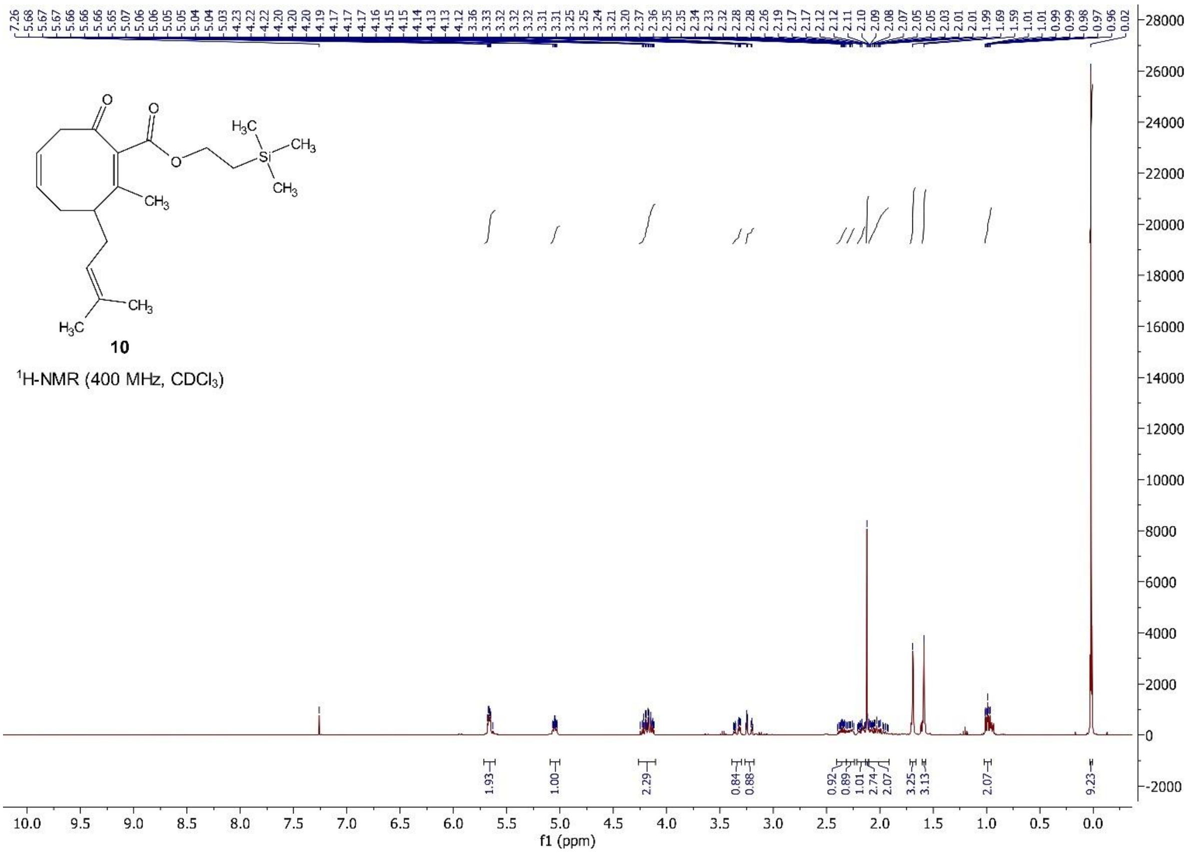
O
O
O
H_3C
Si
CH_3
CH_3
CH_3
H_3C
CH_3
10
^{1}H-NMR (400 MHz, CDCl$_3$)
f1 (ppm)

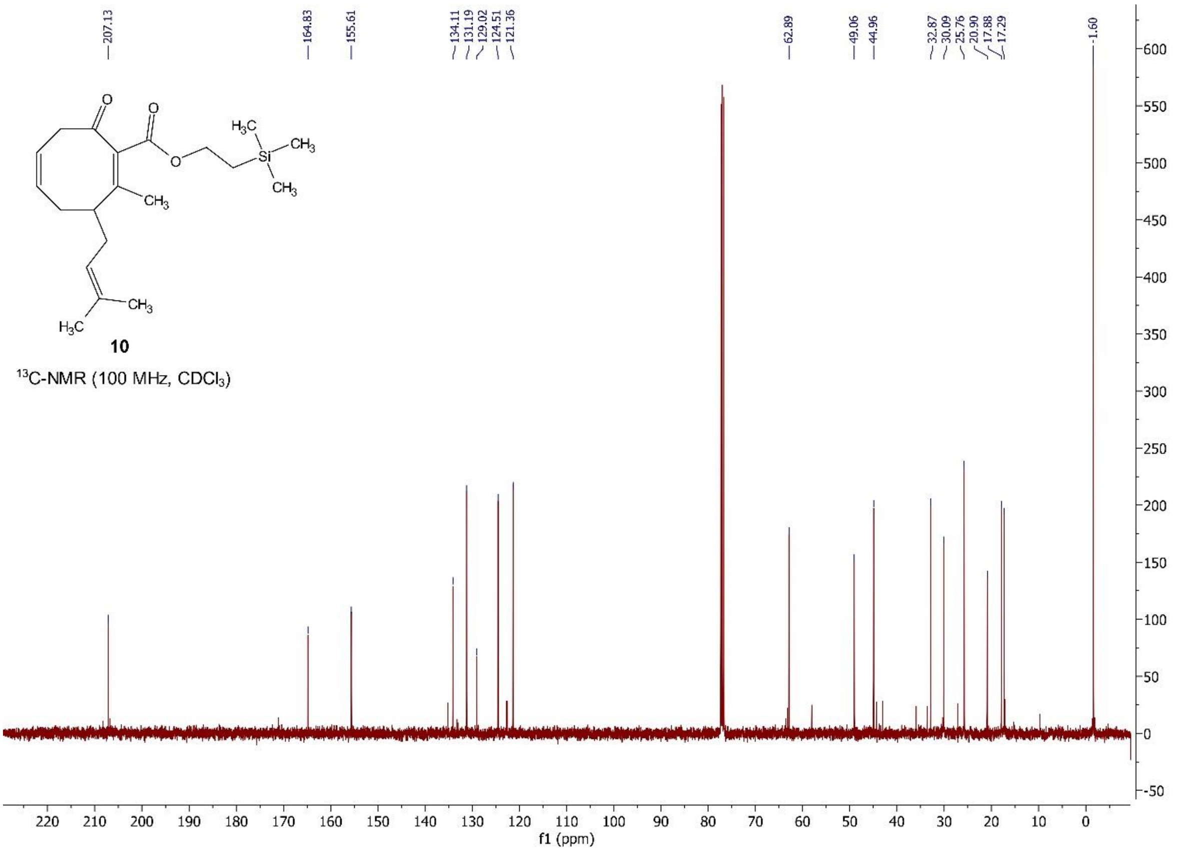
207.13
164.83
155.61
134.11
131.19
129.02
124.51
121.36
62.89
49.06
44.96
32.87
30.09
25.76
20.90
17.88
17.29
-1.60
O
O
O
H_3C
Si
CH_3
CH_3
CH_3
H_3C
CH_3
10
^{13}C-NMR (100 MHz, CDCl$_3$)
f1 (ppm)

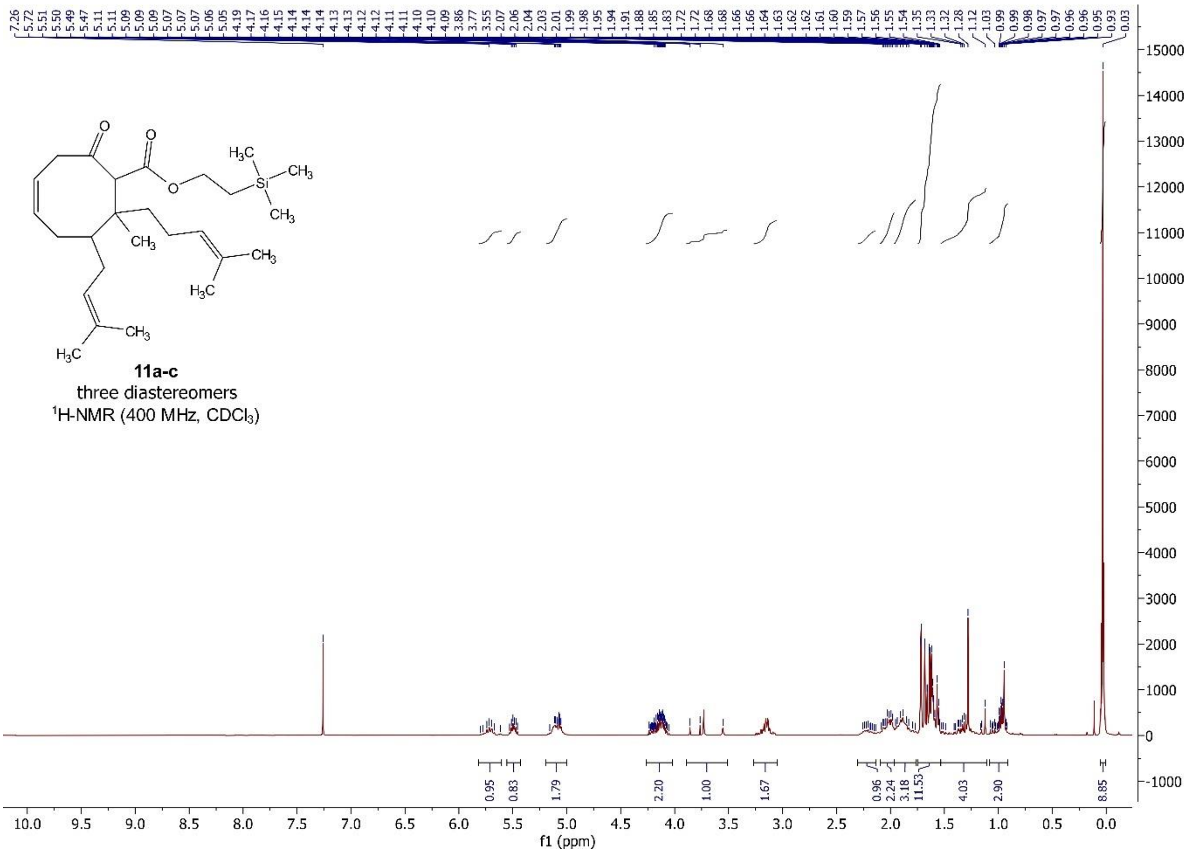

11a-c
three diastereomers
1H-NMR (400 MHz, CDCl3)
f1 (ppm)

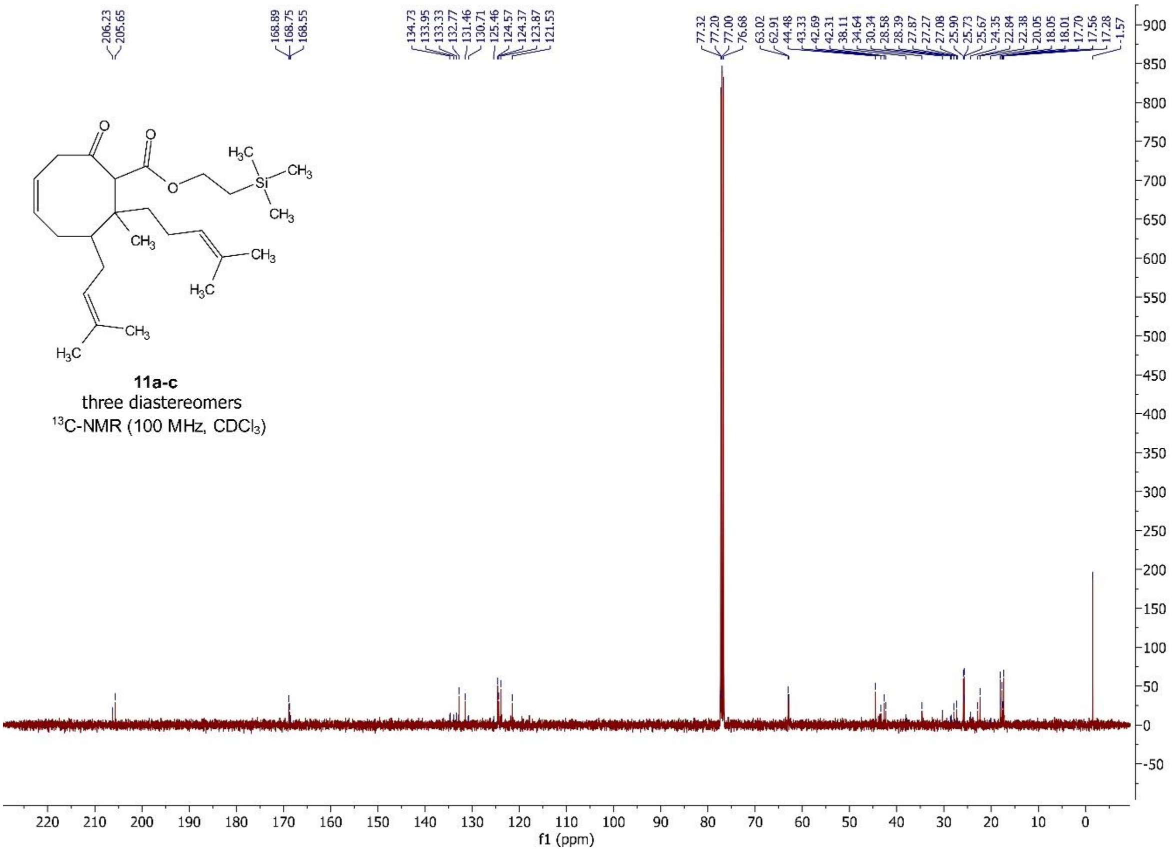

11a-c
three diastereomers
13C-NMR (100 MHz, CDCl3)
f1 (ppm)

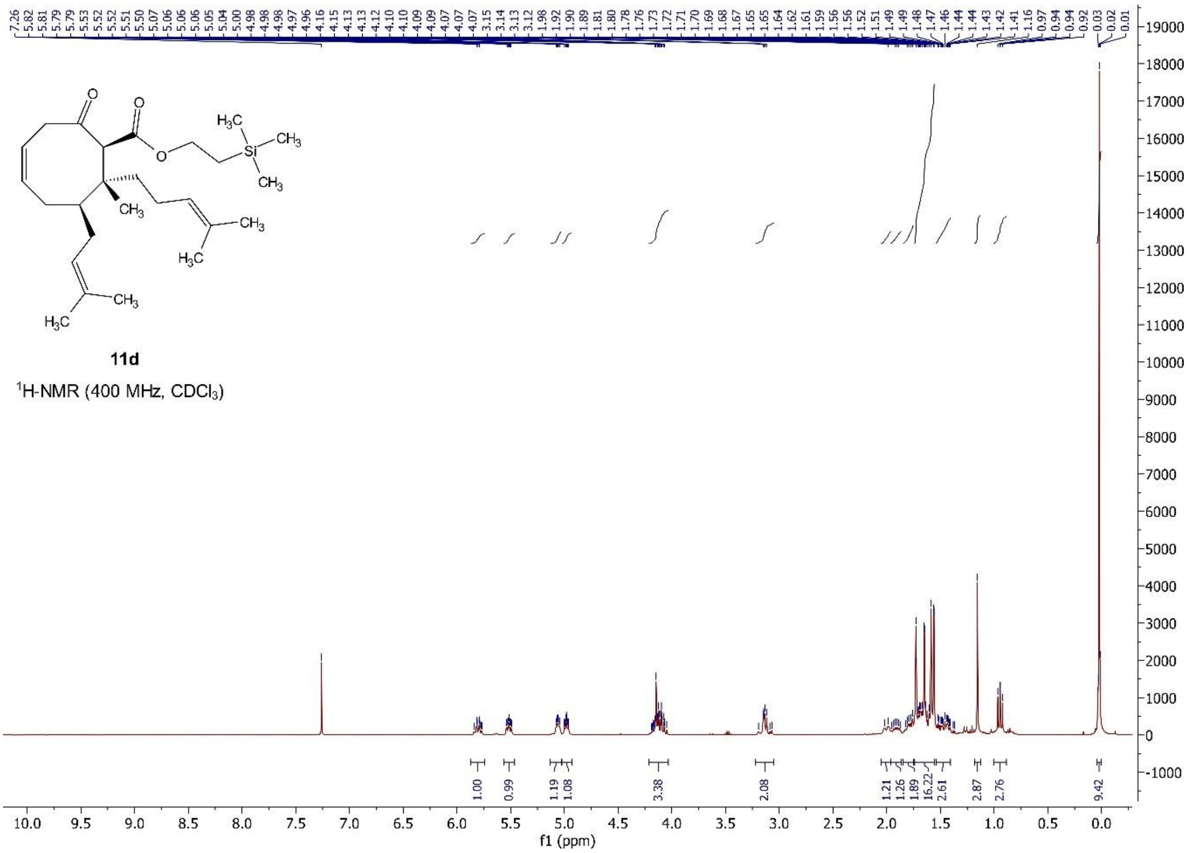

11d

^{1}H-NMR (400 MHz, $CDCl_3$)

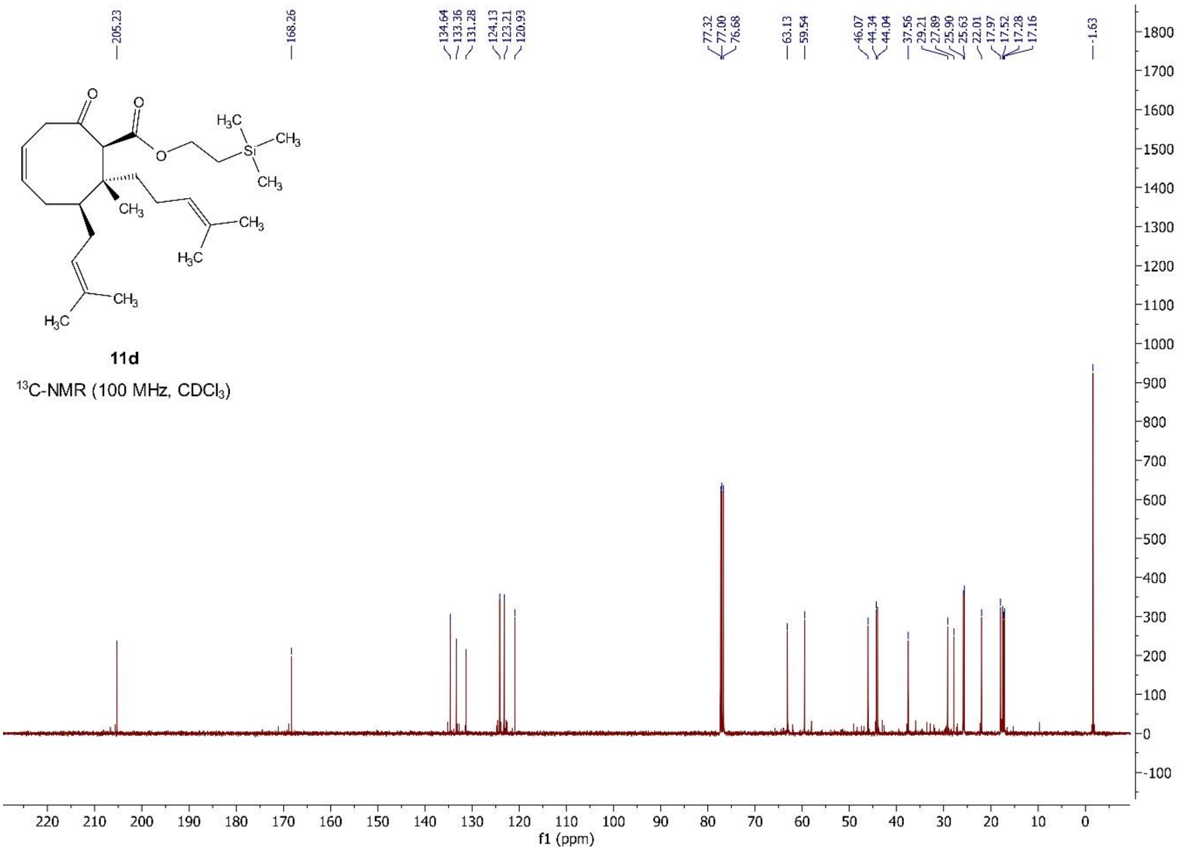

11d

^{13}C-NMR (100 MHz, $CDCl_3$)

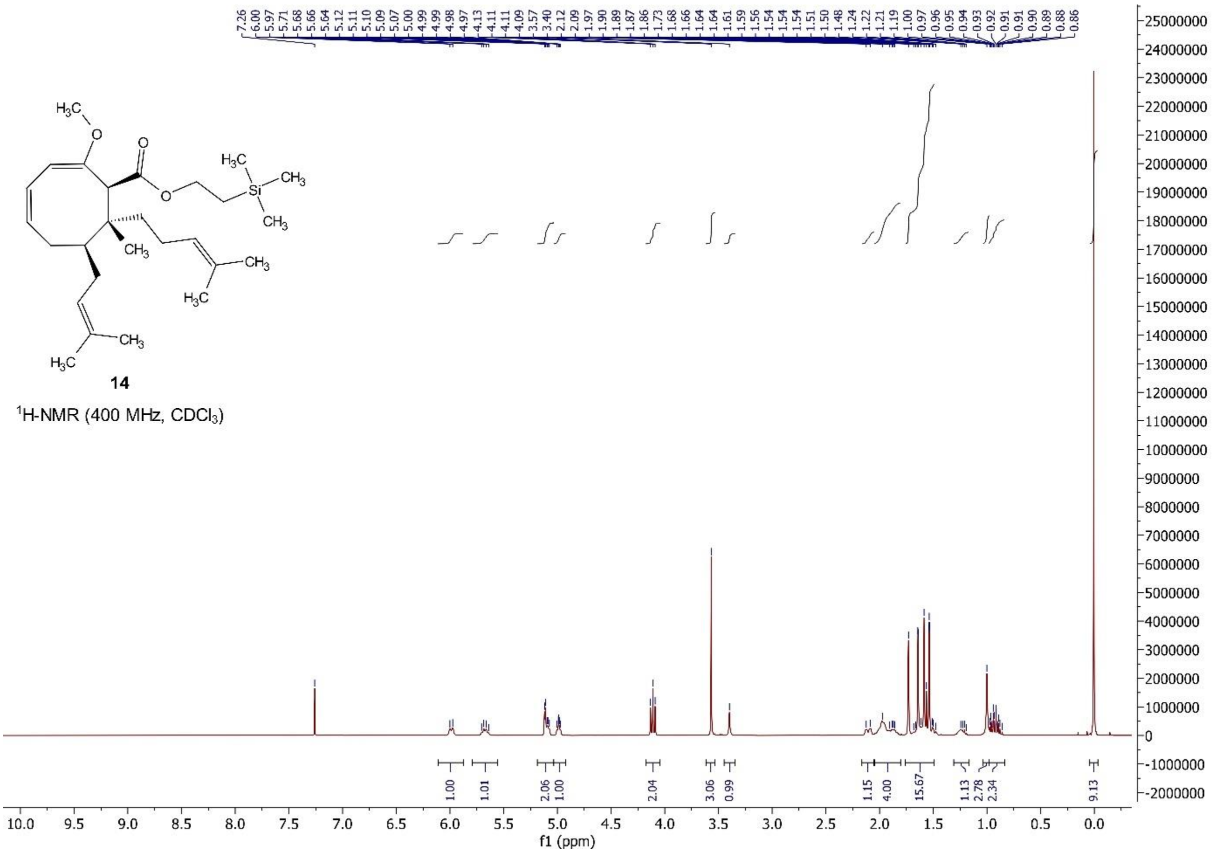

14

^{1}H-NMR (400 MHz, $CDCl_3$)

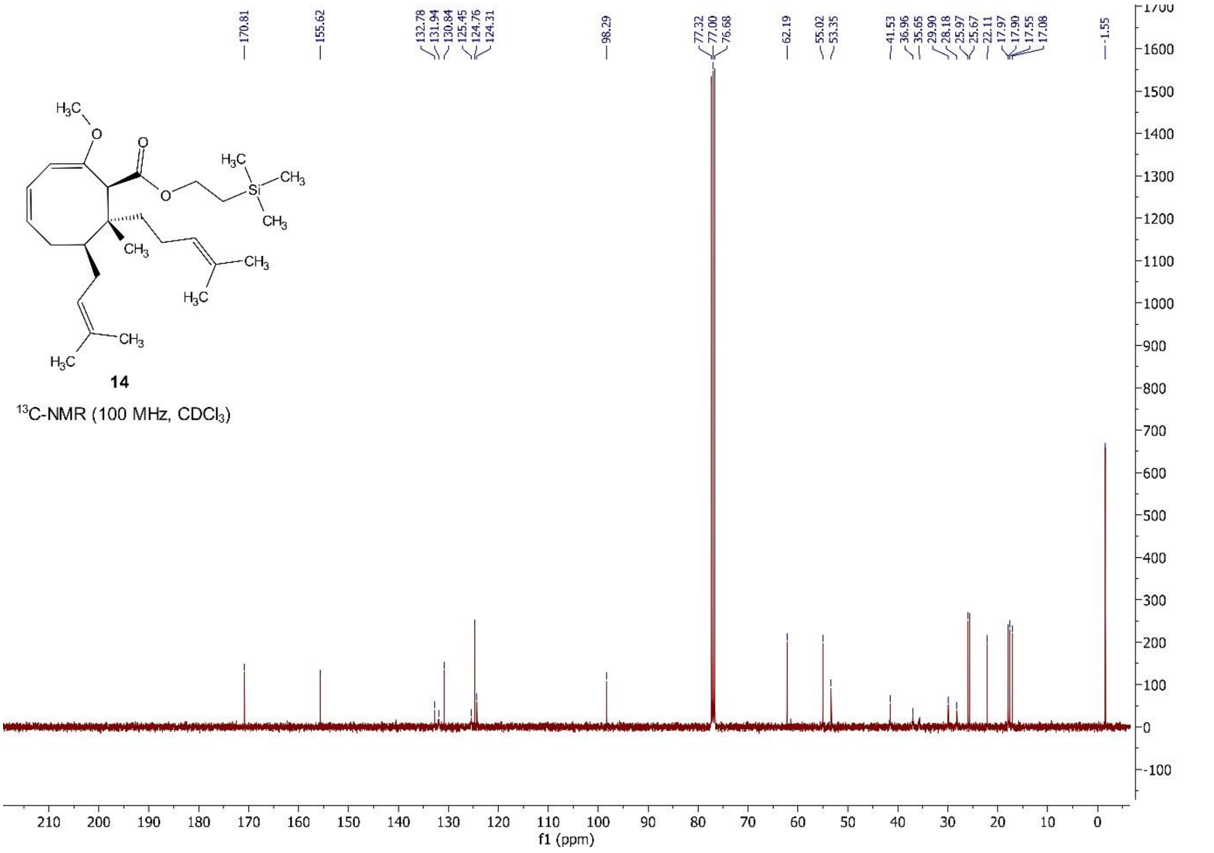

14

^{13}C-NMR (100 MHz, $CDCl_3$)

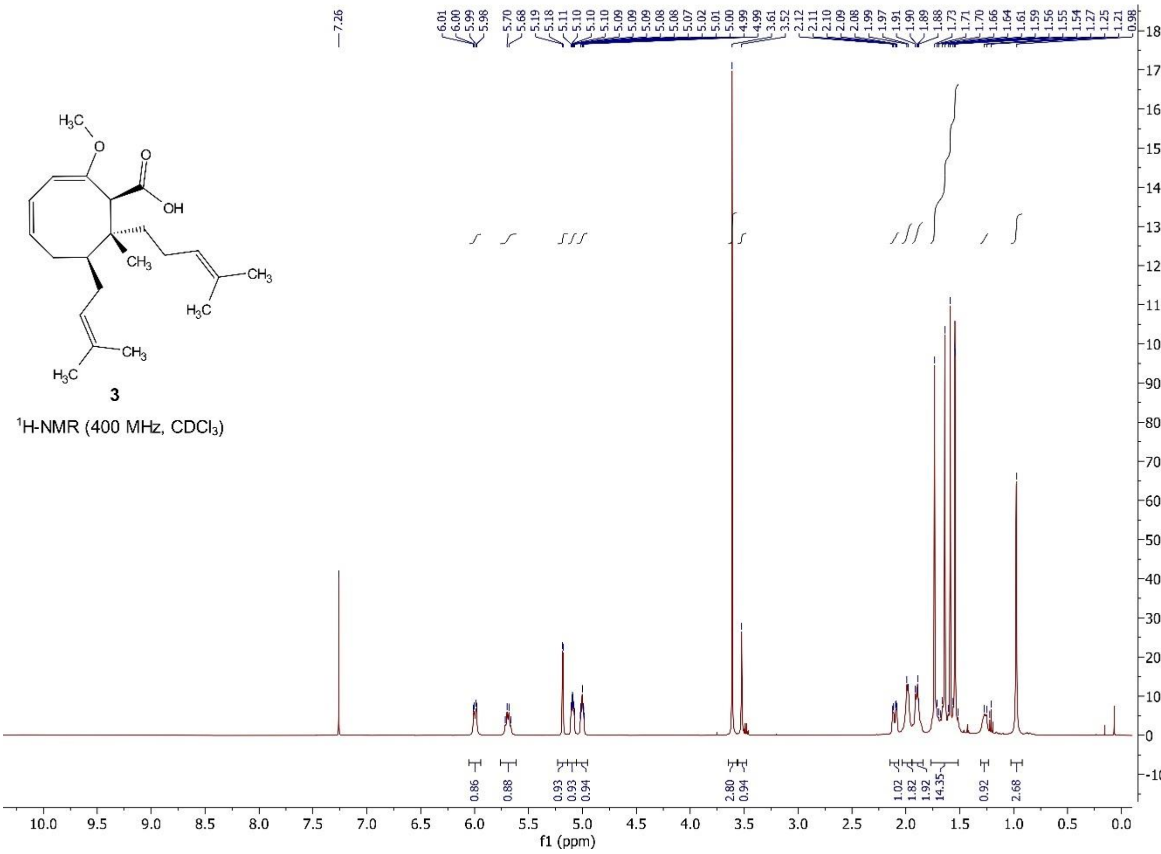

3
1H-NMR (400 MHz, CDCl3)
7.26
6.01 6.00 5.99 5.98 5.70 5.68 5.19 5.18 5.11 5.10 5.10 5.09 5.09 5.09 5.08 5.08 5.07 5.02 5.01 5.00 4.99 4.99 3.61 3.52 2.12 2.11 2.10 2.09 2.08 1.99 1.97 1.91 1.90 1.89 1.88 1.73 1.71 1.70 1.66 1.64 1.61 1.59 1.56 1.55 1.54 1.27 1.25 1.21 0.98
0.86 0.88 0.93 0.93 0.94 2.80 0.94 1.02 1.82 1.92 14.35 0.92 2.68
f1 (ppm)

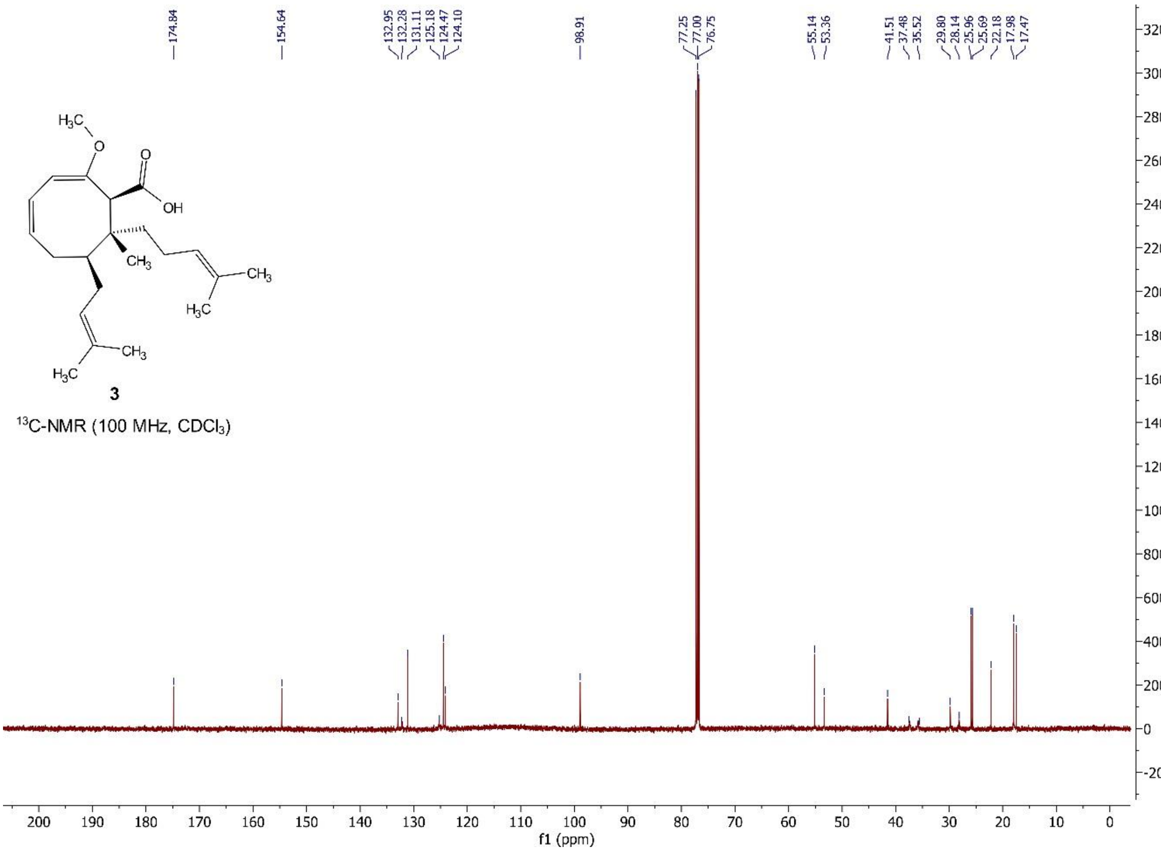

3
13C-NMR (100 MHz, CDCl3)
174.84
154.64
132.95 132.28 131.11 125.18 124.47 124.10
98.91
77.25 77.00 76.75
55.14 53.36
41.51 37.48 35.52 29.80 28.14 25.96 25.69 22.18 17.98 17.47
f1 (ppm)

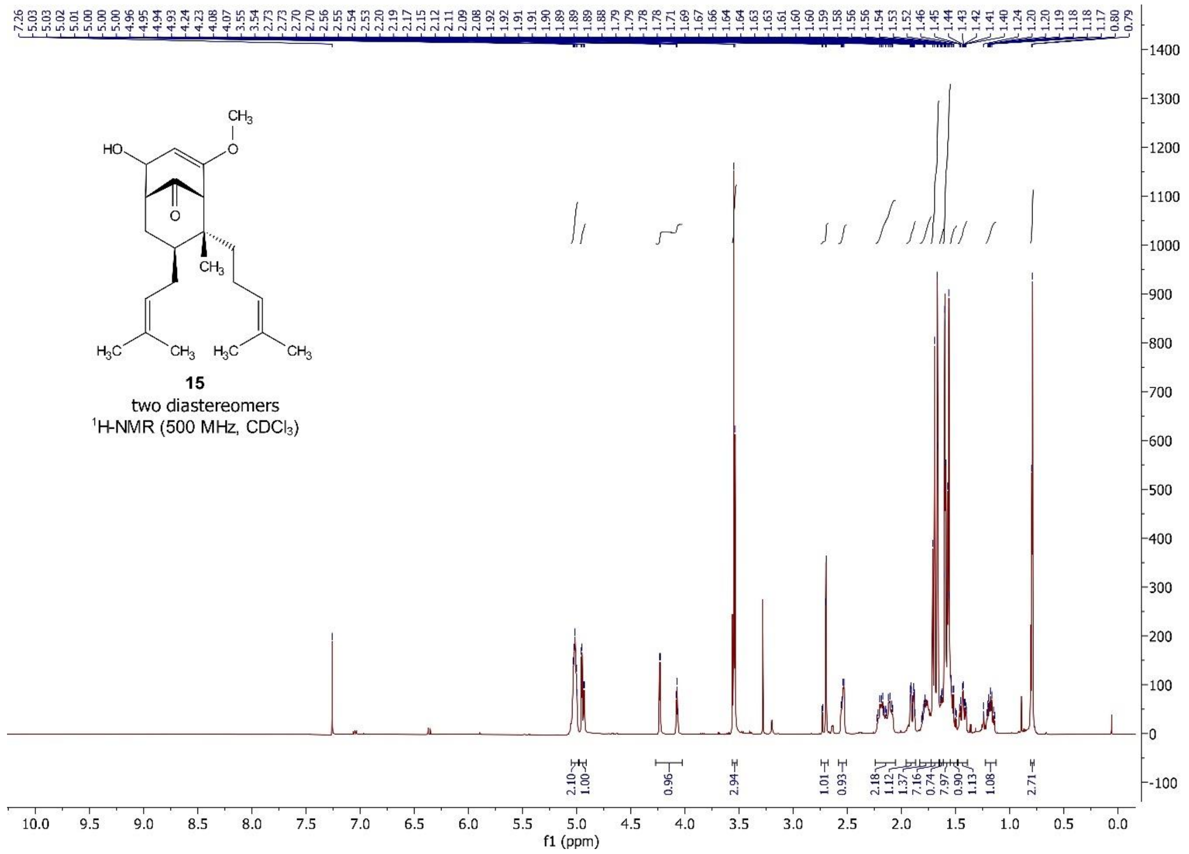
7.26 5.03 5.03 5.02 5.01 5.00 5.00 5.00 4.96 4.95 4.94 4.93 4.24 4.23 4.08 4.07 3.55 3.54 2.73 2.73 2.70 2.70 2.56 2.55 2.54 2.53 2.20 2.19 2.17 2.15 2.12 2.11 2.09 2.08 1.92 1.92 1.91 1.91 1.90 1.89 1.89 1.89 1.88 1.79 1.79 1.78 1.78 1.71 1.69 1.67 1.66 1.64 1.64 1.63 1.63 1.61 1.60 1.60 1.59 1.58 1.56 1.56 1.54 1.53 1.52 1.46 1.45 1.44 1.43 1.42 1.41 1.40 1.24 1.20 1.20 1.19 1.18 1.18 1.17 0.80 0.79
HO
CH_3
O
H_3C
15
two diastereomers
^{1}H-NMR (500 MHz, $CDCl_3$)
2.10 1.00 0.96 2.94 1.01 0.93 2.18 1.12 1.37 7.16 0.74 7.97 0.90 1.13 1.08 2.71
f1 (ppm)
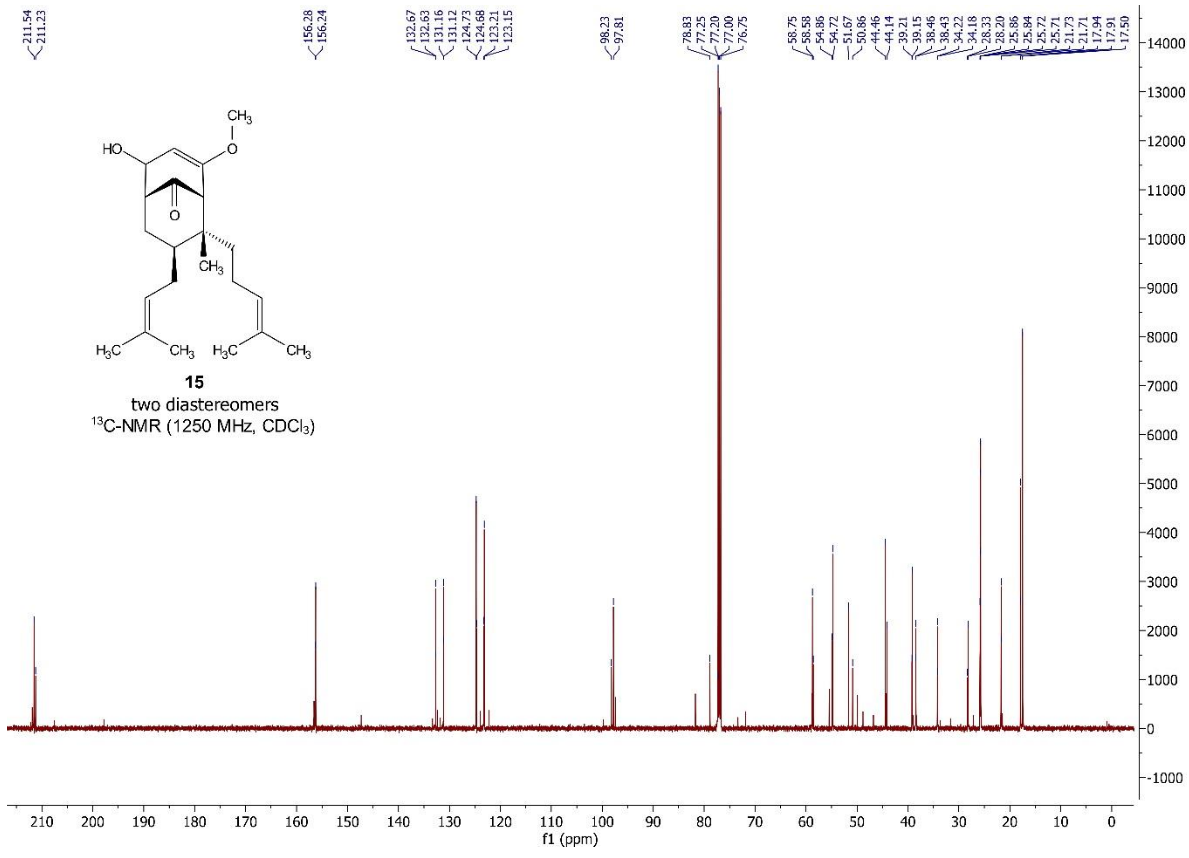
211.54 211.23 156.28 156.24 132.67 132.63 131.16 131.12 124.73 124.68 123.21 123.15 98.23 97.81 78.83 77.25 77.20 77.00 76.75 58.75 58.58 54.86 54.72 51.67 50.86 44.46 44.14 39.21 39.15 38.46 38.43 34.22 34.18 28.33 28.20 25.86 25.84 25.72 25.71 21.73 21.71 17.94 17.91 17.50
HO
CH_3
O
H_3C
15
two diastereomers
^{13}C-NMR (1250 MHz, $CDCl_3$)
f1 (ppm)

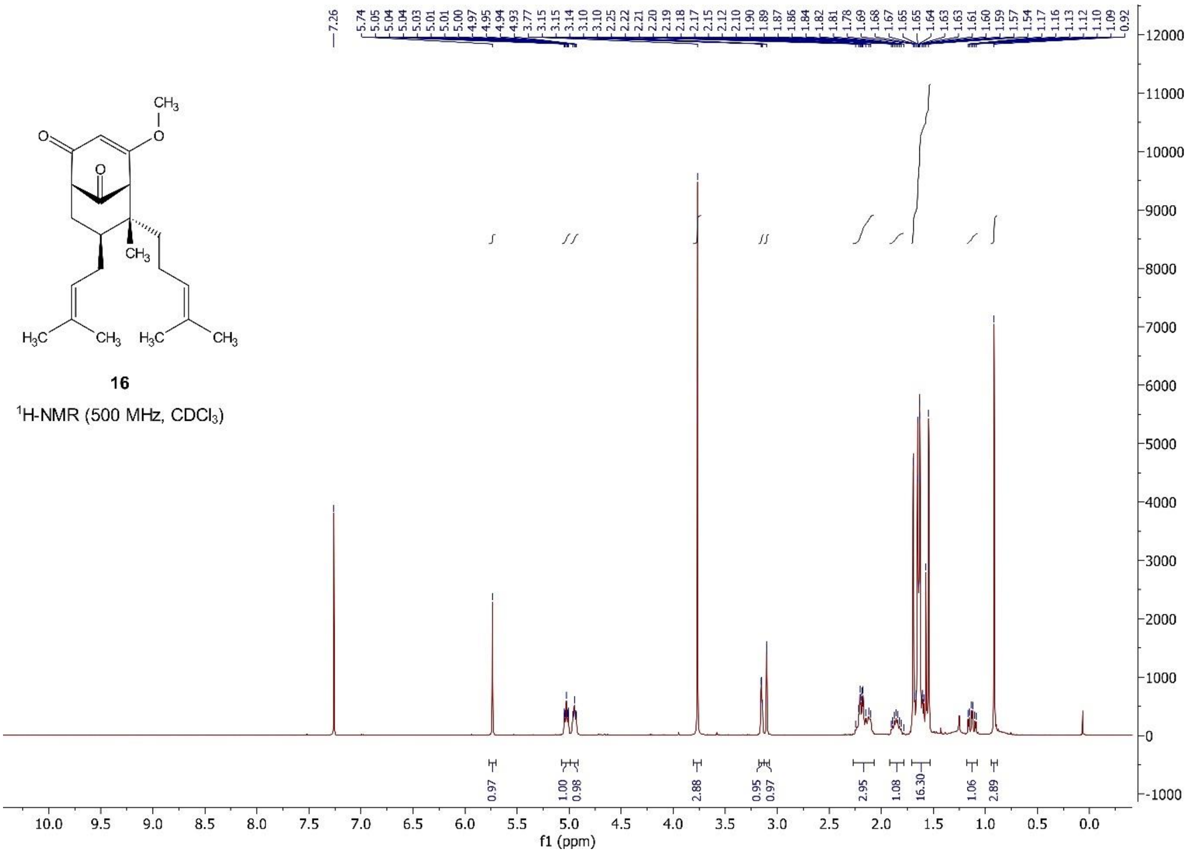

16

^{1}H-NMR (500 MHz, $CDCl_3$)

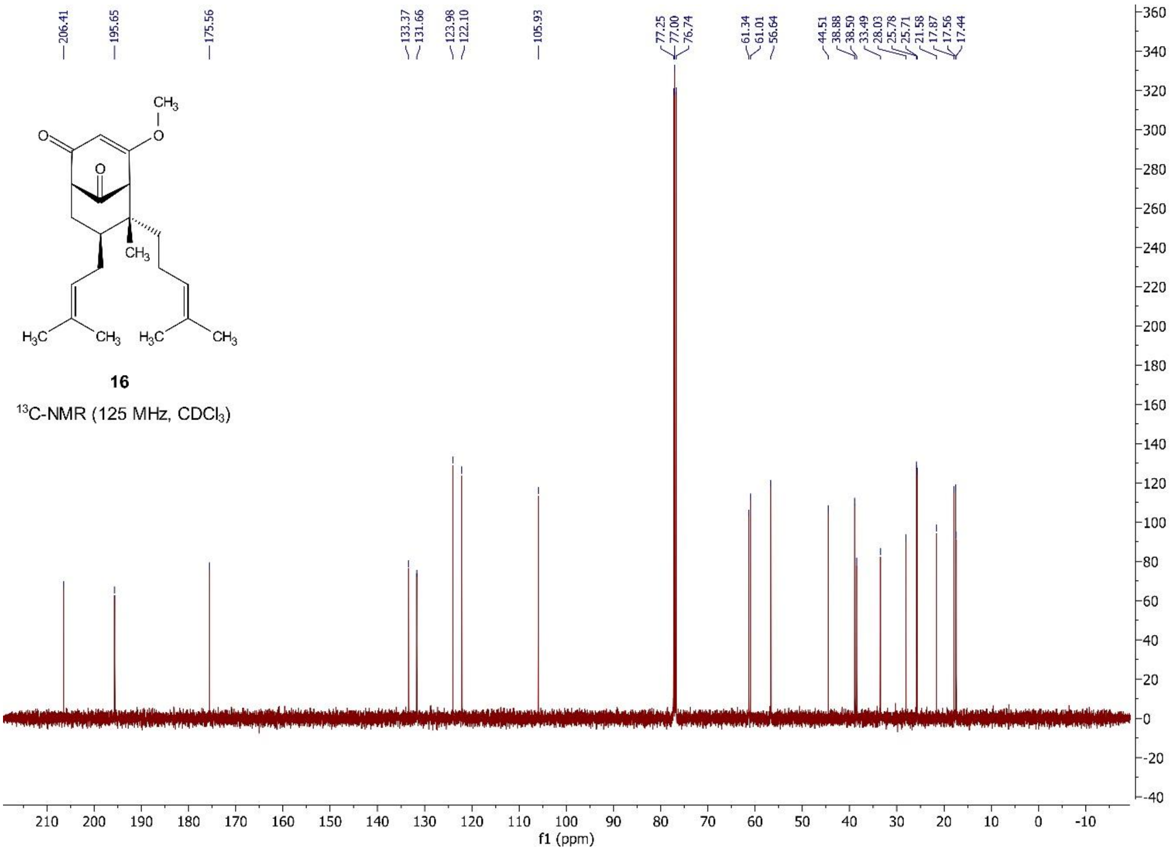

16

^{13}C-NMR (125 MHz, $CDCl_3$)

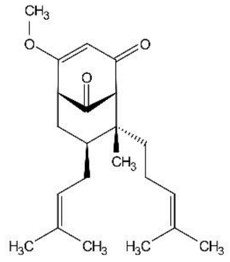

17

^{1}H-NMR (400 MHz, CDCl$_3$)

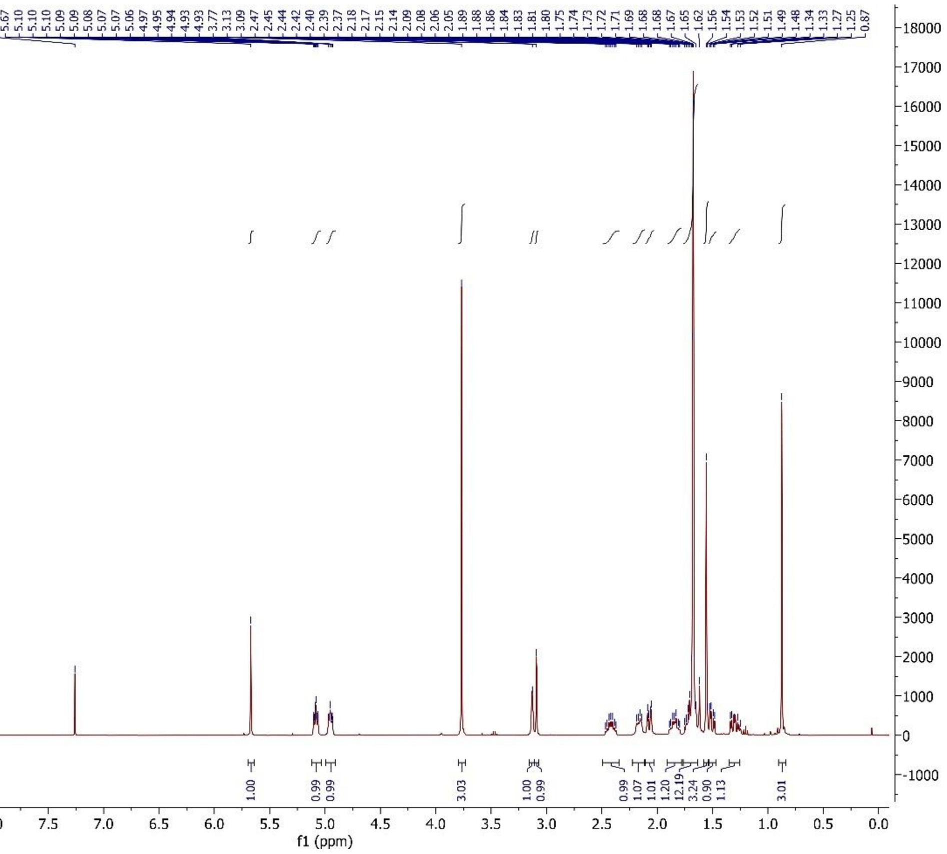

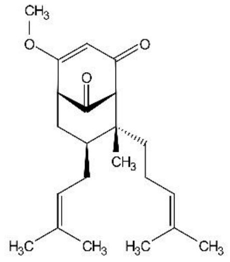

17

^{13}C-NMR (100 MHz, CDCl$_3$)

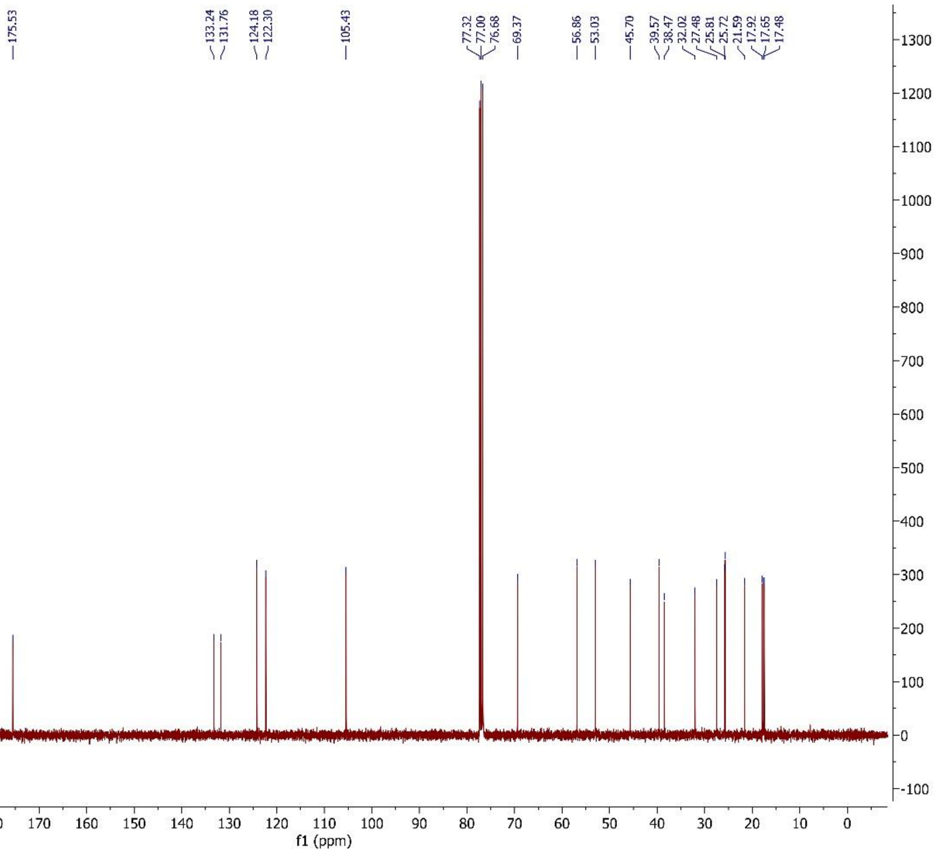

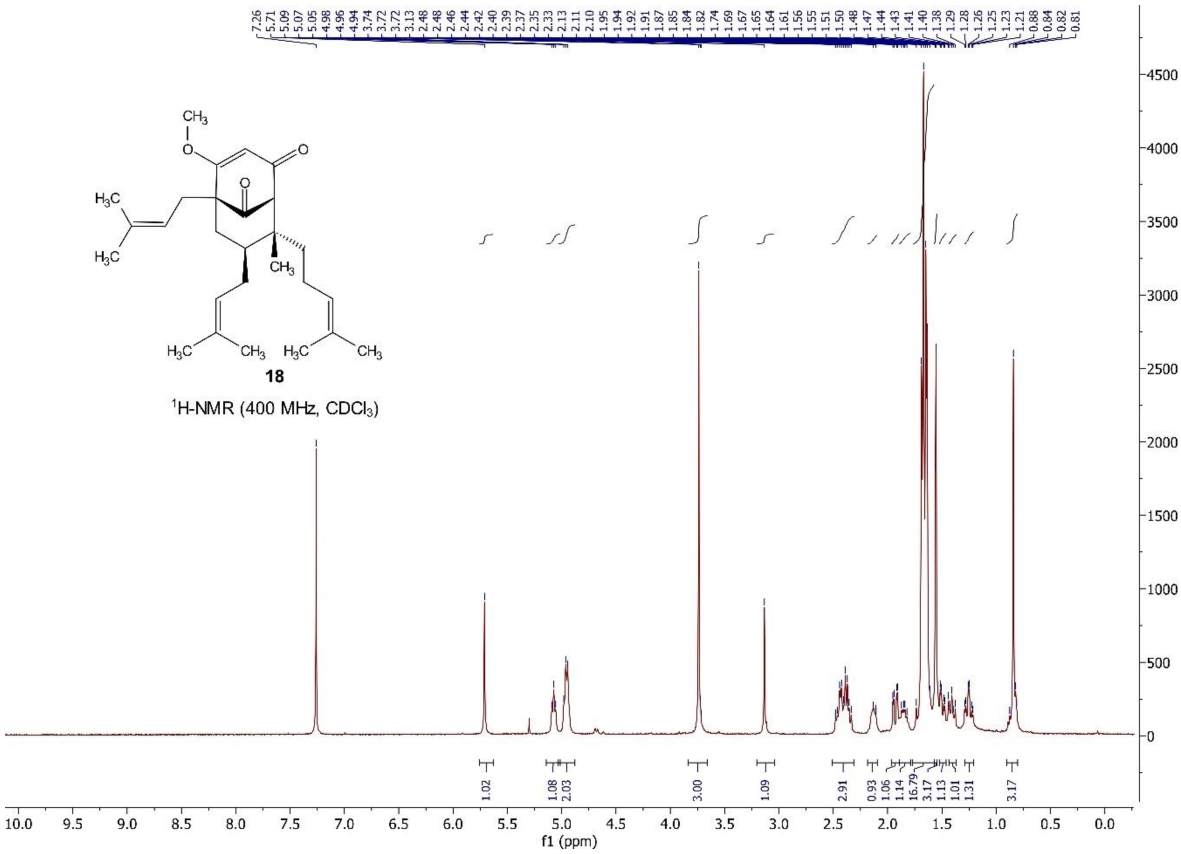
18
1H-NMR (400 MHz, CDCl3)
f1 (ppm)

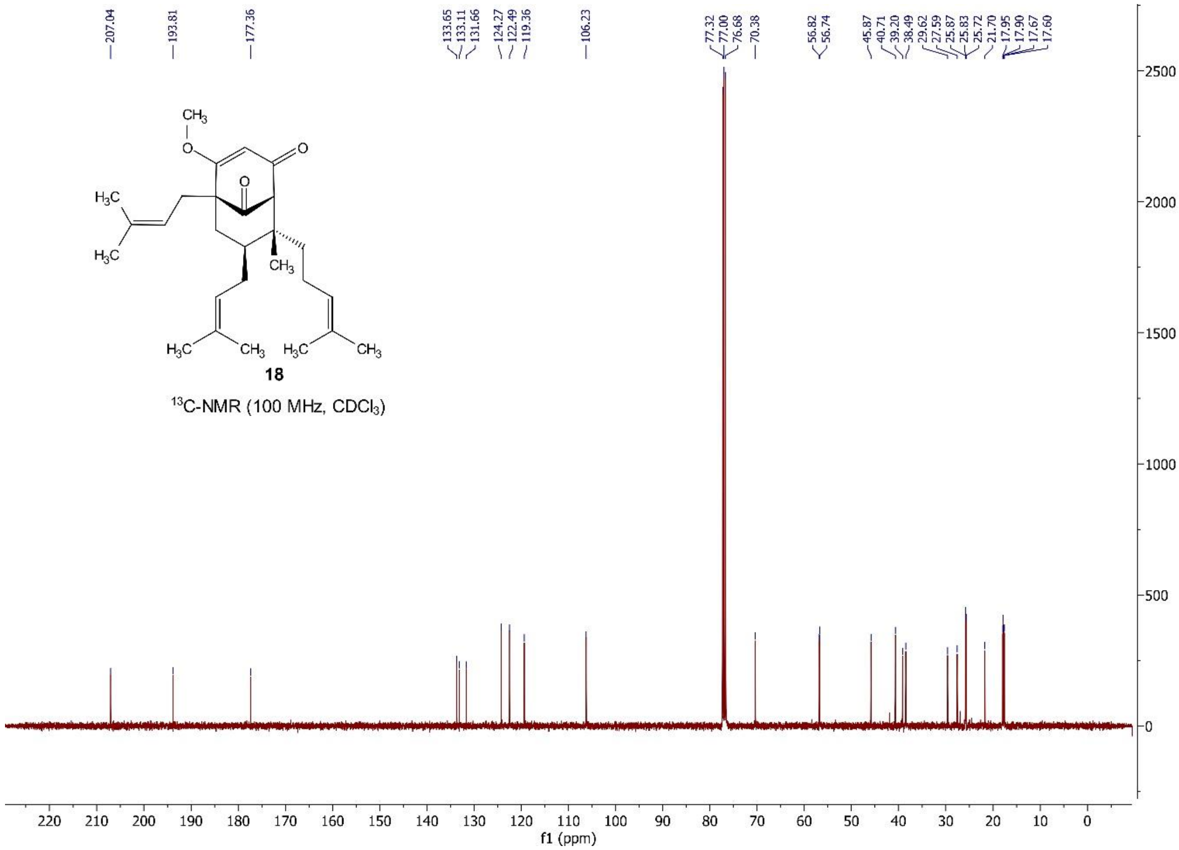
207.04
193.81
177.36
133.65
133.11
131.66
124.27
122.49
119.36
106.23
77.32
77.00
76.68
70.38
56.82
56.74
45.87
40.71
39.20
38.49
29.62
27.59
25.87
25.83
25.72
21.70
17.95
17.90
17.67
17.60
18
13C-NMR (100 MHz, CDCl3)
f1 (ppm)

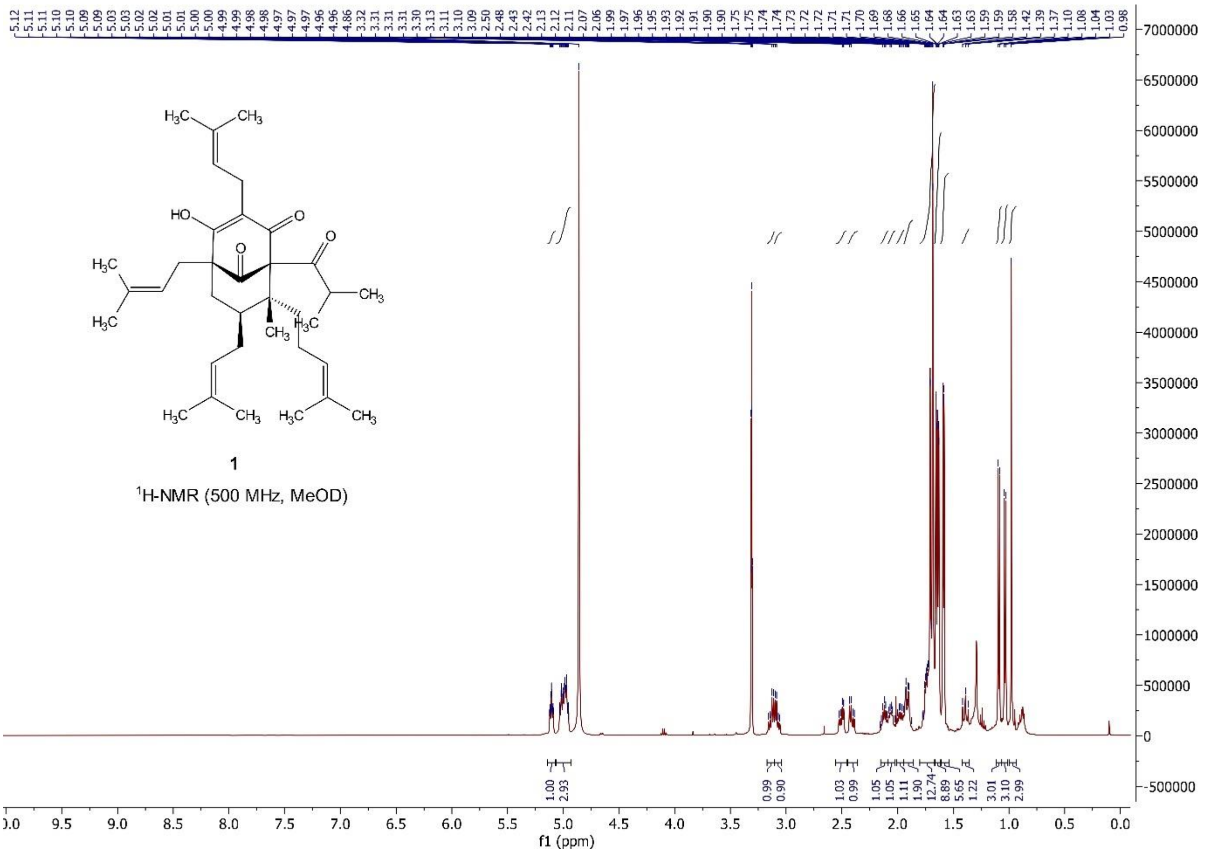
1
1H-NMR (500 MHz, MeOD)
f1 (ppm)

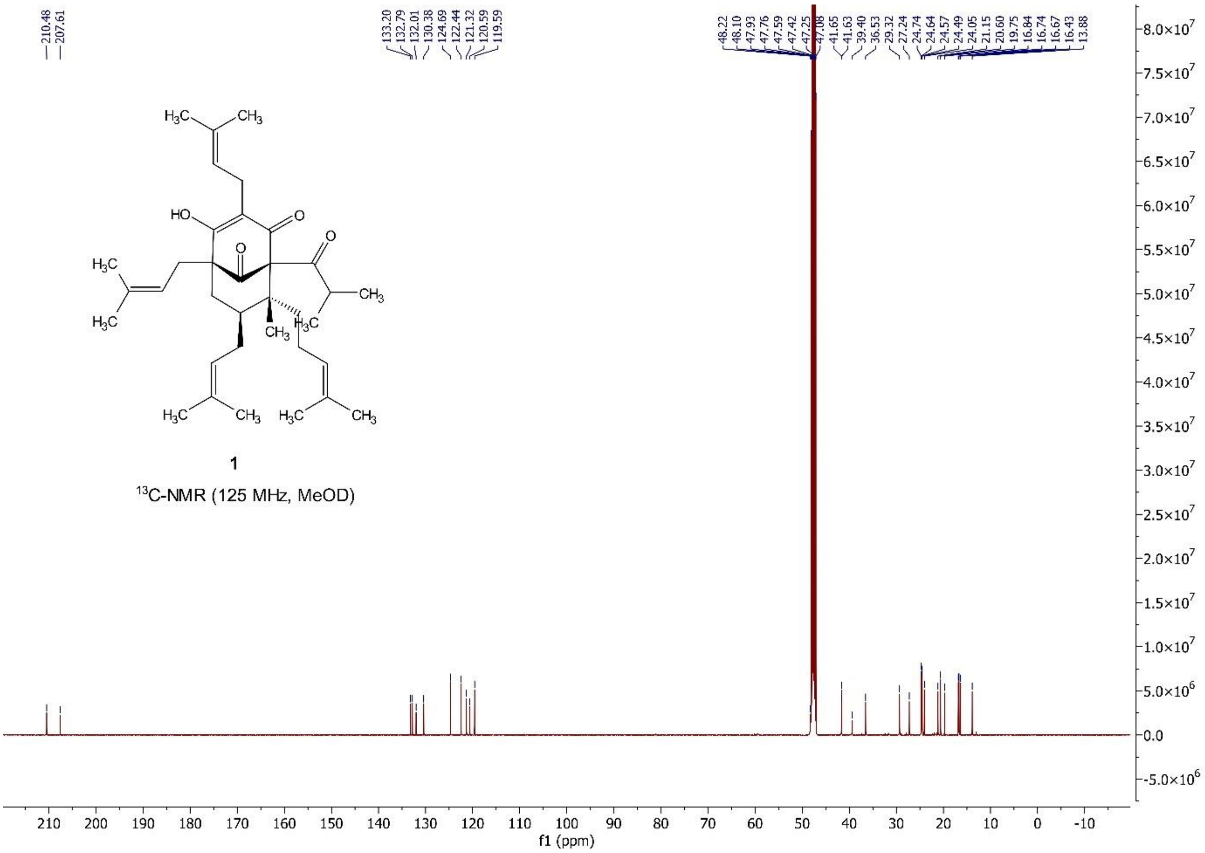
1
13C-NMR (125 MHz, MeOD)
f1 (ppm)

X-ray crystallography

All compounds were crystallized after flash chromatography by dissolving 10 mg to 30 mg of the respective purified compound in a 10 mL round bottom flask with 1 mL to 2 mL DCM. The flasks were topped with a septum and a cannula to allow slow solvent evaporation. All solutions were left undisturbed at room temperature for two to four weeks to yield colorless to pale yellow crystals.

The data set for **3**, and **14** was collected using a Bruker X8 ApexII diffractometer. Graphite-monochromated MoK_α radiation (λ = 0.71073 Å) was used. Data were collected at 152(2) K and corrected for absorption effects using the multi-scan method.

The data set for **16** was collected using a Bruker D8 Venture diffractometer with a microfocus sealed tube and a Photon II detector. Monochromated MoK_α radiation (λ = 0.71073 Å) was used. Data were collected at 143(2) K and corrected for absorption effects using the multi-scan method.

The structures were solved by direct methods using SHELXT[13] and were refined by full matrix least squares calculations on F^2 (SHELXL2018[14]) in the graphical user interface Shelxle[15].

All non H-atoms were located in the electron density maps and refined anisotropically. C-bound H atoms were placed in positions of optimized geometry and were treated as riding atoms. Their isotropic displacement parameters were coupled to the corresponding carrier atoms by a factor of 1.2 (CH, CH_2) or 1.5 (CH_3).

For the refinement of compound **3**, hydrogen atom H2 was located in the electron density maps. Its positional parameters were refined using isotropic displacement parameters, which were set at 1.2 times the Ueq value of its parent atom O2. Restraints of 0.84 (0.01) Å were used for the O-H bond lengths.

Disorder: The O3-C22-C23 group of compound **14** is split over two positions. Its occupancy factors refined to 0.42 for the minor component (O3A, C22A, C23A).

[13] G. M. Sheldrick, *Acta Crystallographica Section A Foundations and Advances* **2015**, *71*, 3–8.
[14] G. M. Sheldrick, *Acta Crystallographica Section C Structural Chemistry* **2015**, *71*, 3–8.
[15] C. B. Hübschle, G. M. Sheldrick, B. Dittrich, *Journal of Applied Crystallography* **2011**, *44*, 1281–1284.

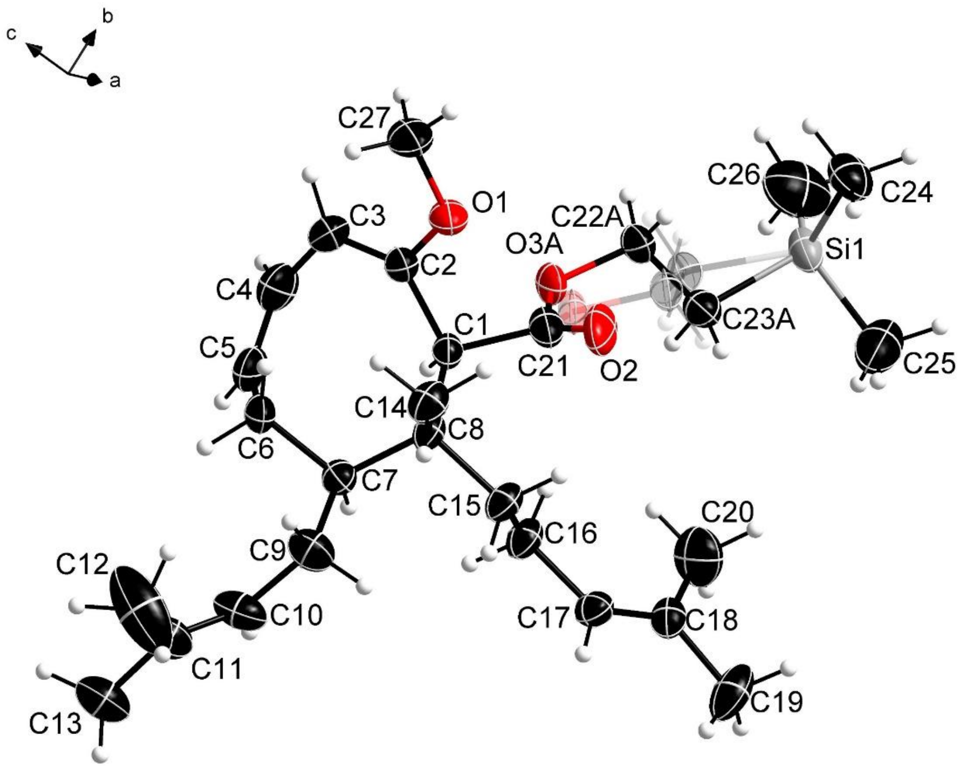

Molecular structure of the enolether **14**. Displacement ellipsoids at a 50% probability level; hydrogen atoms are shown as grey spheres of arbitrary size. One of the split positions is greyed out.

Table 3: Crystal data and structure refinement for enolether **14** (CCDC: 2362034).

Identification code	sh5200_a	
Empirical formula	C27 H46 O3 Si	
Formula weight	446.73	
Temperature	152(2) K	
Wavelength	0.71073 Å	
Crystal system	Triclinic	
Space group	P-1	
Unit cell dimensions	a = 10.9055(10) Å	α = 93.778(4)°.
	b = 11.4047(10) Å	β = 104.967(4)°.
	c = 11.6429(11) Å	γ = 95.563(4)°.
Volume	1386.1(2) $Å^3$	
Z	2	
Density (calculated)	1.070 Mg/m^3	
Absorption coefficient	0.108 mm^{-1}	

F(000)	492
Crystal size	0.400 x 0.200 x 0.030 mm^3
Theta range for data collection	1.802 to 27.194°.
Index ranges	-14<=h<=13, -14<=k<=13, -14<=l<=14
Reflections collected	21176
Independent reflections	6139 [R(int) = 0.0495]
Completeness to theta = 25.242°	100.0 %
Absorption correction	Semi-empirical from equivalents
Max. and min. transmission	0.7455 and 0.7016
Refinement method	Full-matrix least-squares on F^2
Data / restraints / parameters	6139 / 83 / 317
Goodness-of-fit on F^2	1.023
Final R indices [I>2sigma(I)]	R1 = 0.0534, wR2 = 0.1093
R indices (all data)	R1 = 0.1053, wR2 = 0.1313
Extinction coefficient	n/a
Largest diff. peak and hole	0.234 and -0.231 e.Å^{-3}

Table 4: Atomic coordinates (x 10^4) and equivalent isotropic displacement parameters ($Å^2$x 10^3) for enolether **14**. U(eq) is defined as one third of the trace of the orthogonalized U^{ij} tensor.

	x	y	z	U(eq)
Si(1)	7532(1)	11418(1)	4448(1)	43(1)
O(1)	8798(1)	8519(1)	9632(1)	44(1)
O(2)	9271(2)	8374(1)	7302(1)	59(1)
C(1)	7550(2)	7279(2)	7927(2)	29(1)
C(2)	7631(2)	7872(2)	9156(2)	36(1)
C(3)	6667(2)	7855(2)	9663(2)	48(1)
C(4)	5460(2)	7105(2)	9166(2)	56(1)
C(5)	5395(2)	5954(2)	8848(2)	50(1)
C(6)	6501(2)	5256(2)	9059(2)	42(1)
C(7)	7090(2)	5040(2)	8006(2)	32(1)
C(8)	8093(2)	6059(2)	7872(2)	30(1)
C(9)	7632(2)	3840(2)	8115(2)	49(1)
C(10)	6618(2)	2806(2)	7911(2)	51(1)
C(11)	6579(2)	1901(2)	8542(2)	48(1)
C(12)	7576(3)	1752(3)	9642(3)	114(2)
C(13)	5501(2)	915(2)	8223(2)	62(1)
C(14)	9359(2)	6096(2)	8840(2)	40(1)
C(15)	8391(2)	5808(2)	6652(2)	36(1)
C(16)	7312(2)	5822(2)	5509(2)	38(1)
C(17)	7769(2)	5481(2)	4440(2)	35(1)
C(18)	7698(2)	6014(2)	3459(2)	36(1)
C(19)	8268(2)	5543(3)	2509(2)	59(1)
C(20)	7092(3)	7113(2)	3200(2)	68(1)
C(21)	8171(2)	8220(2)	7307(2)	42(1)
O(3A)	7136(7)	9011(6)	6960(6)	39(2)
C(22A)	7481(7)	10108(5)	6436(5)	41(2)
C(23A)	7349(7)	9927(6)	5128(5)	39(2)
O(3B)	7400(5)	8805(5)	6655(4)	36(1)
C(22B)	7969(4)	9691(4)	6047(4)	36(1)
C(23B)	6909(4)	10320(4)	5345(4)	36(1)
C(24)	9063(2)	12254(2)	5363(2)	54(1)
C(25)	7762(3)	10862(3)	3012(2)	76(1)

C(26)	6210(2)	12358(3)	4222(3)	88(1)
C(27)	9077(3)	9046(2)	10833(2)	59(1)

Table 5: Bond lengths [Å] and angles [°] for enolether **14**.

Si(1)-C(25)	1.836(3)
Si(1)-C(26)	1.854(3)
Si(1)-C(24)	1.863(2)
Si(1)-C(23B)	1.869(4)
Si(1)-C(23A)	1.937(6)
O(1)-C(2)	1.367(2)
O(1)-C(27)	1.430(2)
O(2)-C(21)	1.198(2)
C(1)-C(2)	1.519(3)
C(1)-C(21)	1.531(3)
C(1)-C(8)	1.567(3)
C(2)-C(3)	1.332(3)
C(3)-C(4)	1.456(3)
C(4)-C(5)	1.331(3)
C(5)-C(6)	1.486(3)
C(6)-C(7)	1.540(3)
C(7)-C(9)	1.543(3)
C(7)-C(8)	1.561(3)
C(8)-C(14)	1.535(3)
C(8)-C(15)	1.552(3)
C(9)-C(10)	1.498(3)
C(10)-C(11)	1.310(3)
C(11)-C(12)	1.482(4)
C(11)-C(13)	1.499(3)
C(15)-C(16)	1.533(3)
C(16)-C(17)	1.498(3)
C(17)-C(18)	1.317(3)
C(18)-C(20)	1.485(3)
C(18)-C(19)	1.497(3)
C(21)-O(3B)	1.255(5)
C(21)-O(3A)	1.503(7)
O(3A)-C(22A)	1.485(7)
C(22A)-C(23A)	1.490(9)
O(3B)-C(22B)	1.452(5)
C(22B)-C(23B)	1.505(6)
C(25)-Si(1)-C(26)	110.89(15)
C(25)-Si(1)-C(24)	108.51(12)

C(26)-Si(1)-C(24)	111.32(12)
C(25)-Si(1)-C(23B)	117.6(2)
C(26)-Si(1)-C(23B)	98.2(2)
C(24)-Si(1)-C(23B)	110.02(16)
C(25)-Si(1)-C(23A)	99.0(2)
C(26)-Si(1)-C(23A)	119.4(3)
C(24)-Si(1)-C(23A)	106.7(2)
C(2)-O(1)-C(27)	118.01(17)
C(2)-C(1)-C(21)	105.01(15)
C(2)-C(1)-C(8)	117.24(15)
C(21)-C(1)-C(8)	113.48(16)
C(3)-C(2)-O(1)	123.98(19)
C(3)-C(2)-C(1)	124.94(19)
O(1)-C(2)-C(1)	110.84(16)
C(2)-C(3)-C(4)	122.6(2)
C(5)-C(4)-C(3)	122.8(2)
C(4)-C(5)-C(6)	125.2(2)
C(5)-C(6)-C(7)	116.76(17)
C(6)-C(7)-C(9)	107.60(16)
C(6)-C(7)-C(8)	115.24(16)
C(9)-C(7)-C(8)	112.50(16)
C(14)-C(8)-C(15)	106.83(15)
C(14)-C(8)-C(7)	111.37(15)
C(15)-C(8)-C(7)	108.26(16)
C(14)-C(8)-C(1)	110.06(16)
C(15)-C(8)-C(1)	110.02(15)
C(7)-C(8)-C(1)	110.22(15)
C(10)-C(9)-C(7)	113.22(18)
C(11)-C(10)-C(9)	128.5(2)
C(10)-C(11)-C(12)	123.9(2)
C(10)-C(11)-C(13)	122.8(2)
C(12)-C(11)-C(13)	113.3(2)
C(16)-C(15)-C(8)	118.23(16)
C(17)-C(16)-C(15)	109.82(16)
C(18)-C(17)-C(16)	129.6(2)
C(17)-C(18)-C(20)	124.6(2)
C(17)-C(18)-C(19)	120.9(2)
C(20)-C(18)-C(19)	114.43(19)
O(2)-C(21)-O(3B)	118.0(3)

O(2)-C(21)-O(3A)	129.7(3)
O(2)-C(21)-C(1)	127.0(2)
O(3B)-C(21)-C(1)	114.6(3)
O(3A)-C(21)-C(1)	102.2(3)
C(22A)-O(3A)-C(21)	116.2(5)
O(3A)-C(22A)-C(23A)	113.6(5)
C(22A)-C(23A)-Si(1)	111.6(4)
C(21)-O(3B)-C(22B)	115.5(4)
O(3B)-C(22B)-C(23B)	107.7(4)
C(22B)-C(23B)-Si(1)	111.0(3)

Table 6: Anisotropic displacement parameters ($Å^2$x 10^3) for enolether **14**. The anisotropic displacement factor exponent takes the form: $-2p^2[h^2 a^{*2}U^{11} + ... + 2 h k a^* b^* U^{12}]$.

	U^{11}	U^{22}	U^{33}	U^{23}	U^{13}	U^{12}
Si(1)	44(1)	43(1)	34(1)	9(1)	4(1)	-12(1)
O(1)	58(1)	40(1)	30(1)	-6(1)	11(1)	-13(1)
O(2)	54(1)	69(1)	51(1)	1(1)	24(1)	-28(1)
C(1)	31(1)	32(1)	25(1)	4(1)	9(1)	-4(1)
C(2)	48(1)	29(1)	31(1)	2(1)	14(1)	-2(1)
C(3)	64(2)	41(1)	43(1)	-4(1)	27(1)	4(1)
C(4)	50(2)	64(2)	62(2)	1(1)	35(1)	7(1)
C(5)	47(1)	58(2)	51(2)	-2(1)	32(1)	-7(1)
C(6)	57(1)	38(1)	32(1)	4(1)	20(1)	-9(1)
C(7)	36(1)	32(1)	26(1)	3(1)	6(1)	0(1)
C(8)	29(1)	40(1)	22(1)	3(1)	8(1)	3(1)
C(9)	52(1)	37(1)	55(2)	6(1)	11(1)	6(1)
C(10)	56(2)	34(1)	52(2)	-2(1)	-4(1)	3(1)
C(11)	44(1)	38(1)	59(2)	5(1)	7(1)	9(1)
C(12)	72(2)	103(3)	136(3)	73(2)	-30(2)	-20(2)
C(13)	64(2)	37(1)	78(2)	2(1)	11(1)	1(1)
C(14)	33(1)	53(1)	30(1)	1(1)	4(1)	4(1)
C(15)	32(1)	49(1)	25(1)	-1(1)	8(1)	3(1)
C(16)	38(1)	50(1)	24(1)	2(1)	8(1)	2(1)
C(17)	40(1)	37(1)	28(1)	0(1)	8(1)	5(1)
C(18)	32(1)	45(1)	26(1)	3(1)	4(1)	-6(1)
C(19)	44(1)	103(2)	27(1)	3(1)	9(1)	0(1)
C(20)	78(2)	68(2)	61(2)	30(1)	14(2)	17(2)
C(21)	50(1)	42(1)	26(1)	2(1)	6(1)	-15(1)
O(3A)	57(3)	34(3)	29(3)	12(2)	15(2)	-2(2)
C(22A)	60(3)	28(3)	37(3)	8(2)	18(2)	1(2)
C(23A)	45(4)	34(3)	35(3)	4(2)	8(2)	0(2)
O(3B)	38(2)	40(2)	31(2)	12(2)	12(2)	-4(2)
C(22B)	38(2)	36(2)	34(2)	10(2)	12(2)	-1(2)
C(23B)	34(2)	34(2)	39(2)	3(2)	12(2)	0(2)
C(24)	54(1)	47(1)	53(2)	4(1)	4(1)	-12(1)
C(25)	66(2)	100(2)	55(2)	-14(2)	22(1)	-27(2)
C(26)	49(2)	81(2)	123(3)	-12(2)	11(2)	-6(2)

C(27)	90(2)	50(2)	30(1)	-7(1)	12(1)	-13(1)

Table 7: Hydrogen coordinates (x 10^4) and isotropic displacement parameters ($Å^2$x 10^3) for enolether **14**.

	x	y	z	U(eq)
H(1)	6623	7137	7488	35
H(3)	6770	8352	10378	57
H(4)	4687	7453	9065	67
H(5)	4577	5546	8456	60
H(6A)	6228	4477	9298	50
H(6B)	7180	5666	9742	50
H(7)	6374	4952	7259	38
H(9A)	8137	3743	7527	58
H(9B)	8218	3844	8922	58
H(10)	5910	2808	7235	62
H(12A)	8197	1266	9439	171
H(12B)	7177	1361	10203	171
H(12C)	8012	2530	10012	171
H(13A)	4847	1091	7525	93
H(13B)	5127	836	8898	93
H(13C)	5825	173	8038	93
H(14A)	9741	5366	8739	60
H(14B)	9197	6168	9630	60
H(14C)	9946	6777	8768	60
H(15A)	9105	6399	6618	43
H(15B)	8703	5022	6639	43
H(16A)	6570	5259	5530	45
H(16B)	7037	6623	5457	45
H(17)	8170	4775	4475	42
H(19A)	7603	5378	1751	88
H(19B)	8629	4811	2736	88
H(19C)	8945	6131	2416	88
H(20A)	7734	7740	3114	102
H(20B)	6739	7360	3858	102
H(20C)	6405	6963	2457	102
H(22A)	8376	10424	6849	49
H(22B)	6927	10708	6582	49
H(23A)	6500	9491	4725	46
H(23B)	8009	9440	4987	46

H(22C)	8410	9310	5505	43
H(22D)	8602	10262	6636	43
H(23C)	6505	10731	5900	43
H(23D)	6249	9733	4804	43
H(24A)	9741	11734	5474	81
H(24B)	8963	12536	6144	81
H(24C)	9291	12932	4953	81
H(25A)	6951	10459	2502	114
H(25B)	8403	10304	3148	114
H(25C)	8056	11526	2618	114
H(26A)	6367	12986	3724	133
H(26B)	6164	12712	4997	133
H(26C)	5401	11872	3824	133
H(27A)	9945	9463	11066	88
H(27B)	9012	8428	11368	88
H(27C)	8465	9608	10888	88

Table 8: Torsion angles [°] for enolether **14**.

C(27)-O(1)-C(2)-C(3)	11.1(3)
C(27)-O(1)-C(2)-C(1)	-174.31(17)
C(21)-C(1)-C(2)-C(3)	132.3(2)
C(8)-C(1)-C(2)-C(3)	-100.6(2)
C(21)-C(1)-C(2)-O(1)	-42.2(2)
C(8)-C(1)-C(2)-O(1)	84.86(19)
O(1)-C(2)-C(3)-C(4)	-176.5(2)
C(1)-C(2)-C(3)-C(4)	9.7(3)
C(2)-C(3)-C(4)-C(5)	49.9(4)
C(3)-C(4)-C(5)-C(6)	6.4(4)
C(4)-C(5)-C(6)-C(7)	-98.0(3)
C(5)-C(6)-C(7)-C(9)	-149.37(18)
C(5)-C(6)-C(7)-C(8)	84.2(2)
C(6)-C(7)-C(8)-C(14)	73.4(2)
C(9)-C(7)-C(8)-C(14)	-50.5(2)
C(6)-C(7)-C(8)-C(15)	-169.45(16)
C(9)-C(7)-C(8)-C(15)	66.7(2)
C(6)-C(7)-C(8)-C(1)	-49.1(2)
C(9)-C(7)-C(8)-C(1)	-172.92(16)
C(2)-C(1)-C(8)-C(14)	-42.0(2)
C(21)-C(1)-C(8)-C(14)	80.81(19)
C(2)-C(1)-C(8)-C(15)	-159.42(16)
C(21)-C(1)-C(8)-C(15)	-36.7(2)
C(2)-C(1)-C(8)-C(7)	81.3(2)
C(21)-C(1)-C(8)-C(7)	-155.97(16)
C(6)-C(7)-C(9)-C(10)	67.7(2)
C(8)-C(7)-C(9)-C(10)	-164.32(18)
C(7)-C(9)-C(10)-C(11)	-133.7(3)
C(9)-C(10)-C(11)-C(12)	1.5(5)
C(9)-C(10)-C(11)-C(13)	-179.3(2)
C(14)-C(8)-C(15)-C(16)	-175.22(18)
C(7)-C(8)-C(15)-C(16)	64.7(2)
C(1)-C(8)-C(15)-C(16)	-55.8(2)
C(8)-C(15)-C(16)-C(17)	-176.20(17)
C(15)-C(16)-C(17)-C(18)	-129.6(2)
C(16)-C(17)-C(18)-C(20)	-1.2(4)
C(16)-C(17)-C(18)-C(19)	177.56(19)

C(2)-C(1)-C(21)-O(2)	91.7(3)
C(8)-C(1)-C(21)-O(2)	-37.6(3)
C(2)-C(1)-C(21)-O(3B)	-95.4(3)
C(8)-C(1)-C(21)-O(3B)	135.4(3)
C(2)-C(1)-C(21)-O(3A)	-77.1(3)
C(8)-C(1)-C(21)-O(3A)	153.6(3)
O(2)-C(21)-O(3A)-C(22A)	5.6(7)
C(1)-C(21)-O(3A)-C(22A)	173.9(4)
C(21)-O(3A)-C(22A)-C(23A)	84.2(8)
O(3A)-C(22A)-C(23A)-Si(1)	170.4(4)
O(2)-C(21)-O(3B)-C(22B)	-5.6(5)
C(1)-C(21)-O(3B)-C(22B)	-179.2(3)
C(21)-O(3B)-C(22B)-C(23B)	-178.3(5)
O(3B)-C(22B)-C(23B)-Si(1)	-176.4(3)
C(25)-Si(1)-C(23B)-C(22B)	81.8(4)
C(26)-Si(1)-C(23B)-C(22B)	-159.4(4)
C(24)-Si(1)-C(23B)-C(22B)	-43.0(5)

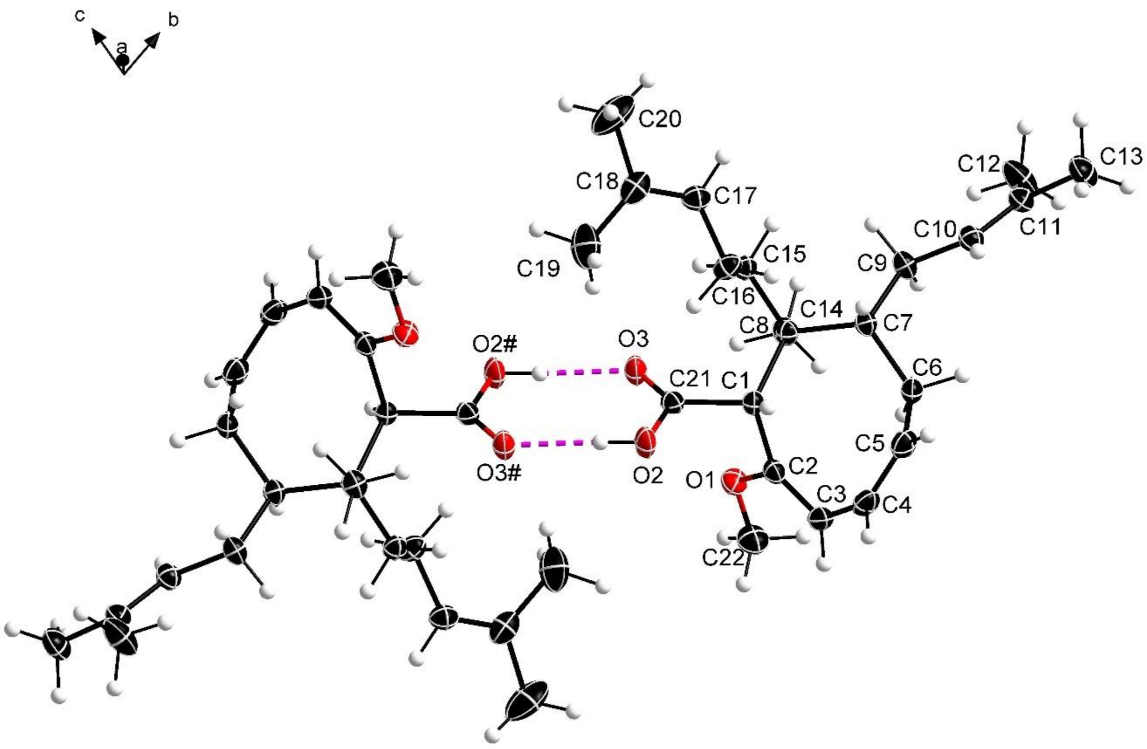

Molecular structure of the carboxylic acid **3**. Displacement ellipsoids at a 50% probability level; hydrogen atoms are shown as grey spheres of arbitrary size.

Table 9: Crystal data and structure refinement for carboxylic acid **3** (CCDC: 2362033).

Identification code	sh5198_a	
Empirical formula	C22 H34 O3	
Formula weight	346.49	
Temperature	152(2) K	
Wavelength	0.71073 Å	
Crystal system	Triclinic	
Space group	P-1	
Unit cell dimensions	a = 7.7374(5) Å	α = 75.647(2)°.
	b = 11.5027(7) Å	β = 72.810(2)°.
	c = 12.4318(8) Å	γ = 86.691(2)°.
Volume	1023.88(11) $Å^3$	
Z	2	
Density (calculated)	1.124 Mg/m^3	
Absorption coefficient	0.073 mm^{-1}	
F(000)	380	

Crystal size	0.300 x 0.240 x 0.200 mm^3
Theta range for data collection	1.767 to 27.144°.
Index ranges	-9<=h<=9, -13<=k<=14, -15<=l<=15
Reflections collected	15031
Independent reflections	4528 [R(int) = 0.0307]
Completeness to theta = 25.242°	100.0 %
Absorption correction	Semi-empirical from equivalents
Max. and min. transmission	0.7455 and 0.6820
Refinement method	Full-matrix least-squares on F^2
Data / restraints / parameters	4528 / 1 / 235
Goodness-of-fit on F^2	1.045
Final R indices [I>2sigma(I)]	R1 = 0.0423, wR2 = 0.0996
R indices (all data)	R1 = 0.0649, wR2 = 0.1110
Extinction coefficient	n/a
Largest diff. peak and hole	0.237 and -0.195 e.Å^{-3}

Table 10: Atomic coordinates (x 10^4) and equivalent isotropic displacement parameters ($Å^2$x 10^3) for carboxylic acid **3**. U(eq) is defined as one third of the trace of the orthogonalized U^{ij} tensor.

	x	y	z	U(eq)
O(2)	6521(1)	537(1)	3501(1)	29(1)
O(3)	4380(1)	1380(1)	4696(1)	27(1)
O(1)	3122(1)	1826(1)	2595(1)	27(1)
C(1)	5853(2)	2544(1)	2727(1)	20(1)
C(5)	7689(2)	4038(1)	212(1)	29(1)
C(6)	6440(2)	4841(1)	864(1)	25(1)
C(7)	6525(2)	4835(1)	2086(1)	20(1)
C(8)	5504(2)	3769(1)	3094(1)	19(1)
C(9)	5829(2)	6054(1)	2325(1)	25(1)
C(10)	7074(2)	7098(1)	1599(1)	25(1)
C(11)	6650(2)	8246(1)	1466(1)	26(1)
C(12)	4797(2)	8662(2)	2002(2)	46(1)
C(13)	8016(2)	9233(1)	795(2)	38(1)
C(14)	3466(2)	3993(1)	3463(1)	24(1)
C(15)	6243(2)	3711(1)	4135(1)	22(1)
C(16)	8161(2)	3236(1)	4038(1)	27(1)
C(17)	8825(2)	3388(1)	5005(1)	26(1)
C(18)	9441(2)	2554(2)	5737(1)	32(1)
C(19)	9565(3)	1258(2)	5714(2)	51(1)
C(20)	10143(3)	2872(2)	6630(2)	60(1)
C(21)	5485(2)	1446(1)	3757(1)	22(1)
C(22)	1900(2)	1671(1)	1982(1)	34(1)
C(2)	4806(2)	2287(1)	1947(1)	22(1)
C(3)	5487(2)	2392(1)	807(1)	26(1)
C(4)	7231(2)	2975(1)	140(1)	29(1)

Table 11: Bond lengths [Å] and angles [°] for carboxylic acid **3**.

O(2)-C(21)	1.3244(16)
O(3)-C(21)	1.2132(16)
O(1)-C(2)	1.3689(16)
O(1)-C(22)	1.4213(17)
C(1)-C(2)	1.5224(18)
C(1)-C(21)	1.5299(18)
C(1)-C(8)	1.5719(18)
C(5)-C(4)	1.323(2)
C(5)-C(6)	1.499(2)
C(6)-C(7)	1.5392(18)
C(7)-C(9)	1.5394(18)
C(7)-C(8)	1.5693(18)
C(8)-C(14)	1.5322(18)
C(8)-C(15)	1.5472(18)
C(9)-C(10)	1.5051(19)
C(10)-C(11)	1.324(2)
C(11)-C(12)	1.498(2)
C(11)-C(13)	1.500(2)
C(15)-C(16)	1.5333(19)
C(16)-C(17)	1.4899(19)
C(17)-C(18)	1.321(2)
C(18)-C(19)	1.495(2)
C(18)-C(20)	1.498(2)
C(2)-C(3)	1.3342(19)
C(3)-C(4)	1.458(2)
C(2)-O(1)-C(22)	117.13(11)
C(2)-C(1)-C(21)	104.67(10)
C(2)-C(1)-C(8)	117.55(11)
C(21)-C(1)-C(8)	113.46(10)
C(4)-C(5)-C(6)	124.91(14)
C(5)-C(6)-C(7)	118.30(12)
C(6)-C(7)-C(9)	107.27(11)
C(6)-C(7)-C(8)	116.09(11)
C(9)-C(7)-C(8)	111.17(10)
C(14)-C(8)-C(15)	108.74(11)
C(14)-C(8)-C(7)	110.79(11)
C(15)-C(8)-C(7)	106.98(10)

C(14)-C(8)-C(1)	109.53(11)
C(15)-C(8)-C(1)	109.44(10)
C(7)-C(8)-C(1)	111.29(10)
C(10)-C(9)-C(7)	113.95(11)
C(11)-C(10)-C(9)	125.76(13)
C(10)-C(11)-C(12)	122.95(14)
C(10)-C(11)-C(13)	122.22(14)
C(12)-C(11)-C(13)	114.81(13)
C(16)-C(15)-C(8)	117.08(11)
C(17)-C(16)-C(15)	112.16(11)
C(18)-C(17)-C(16)	127.81(14)
C(17)-C(18)-C(19)	123.68(15)
C(17)-C(18)-C(20)	120.99(17)
C(19)-C(18)-C(20)	115.29(16)
O(3)-C(21)-O(2)	122.79(12)
O(3)-C(21)-C(1)	125.77(12)
O(2)-C(21)-C(1)	111.44(11)
C(3)-C(2)-O(1)	124.56(13)
C(3)-C(2)-C(1)	124.79(13)
O(1)-C(2)-C(1)	110.39(11)
C(2)-C(3)-C(4)	122.42(13)
C(5)-C(4)-C(3)	122.77(13)

Table 12: Anisotropic displacement parameters ($Å^2$x 10^3) for carboxylic acid **3**. The anisotropic displacement factor exponent takes the form: $-2p^2[h^2 a^{*2}U^{11} + ... + 2 h k a^* b^* U^{12}]$.

	U^{11}	U^{22}	U^{33}	U^{23}	U^{13}	U^{12}
O(2)	38(1)	18(1)	24(1)	-1(1)	-4(1)	7(1)
O(3)	31(1)	21(1)	23(1)	-1(1)	-3(1)	2(1)
O(1)	26(1)	28(1)	28(1)	-4(1)	-9(1)	-6(1)
C(1)	21(1)	18(1)	19(1)	-2(1)	-5(1)	1(1)
C(5)	29(1)	35(1)	20(1)	-4(1)	-3(1)	-4(1)
C(6)	31(1)	21(1)	22(1)	1(1)	-9(1)	-3(1)
C(7)	19(1)	19(1)	22(1)	-3(1)	-6(1)	1(1)
C(8)	20(1)	18(1)	20(1)	-4(1)	-5(1)	2(1)
C(9)	26(1)	18(1)	30(1)	-3(1)	-7(1)	0(1)
C(10)	24(1)	23(1)	28(1)	-5(1)	-9(1)	-2(1)
C(11)	31(1)	23(1)	27(1)	-5(1)	-10(1)	-4(1)
C(12)	40(1)	25(1)	65(1)	-13(1)	-2(1)	0(1)
C(13)	42(1)	26(1)	44(1)	-4(1)	-8(1)	-9(1)
C(14)	20(1)	22(1)	28(1)	-4(1)	-4(1)	1(1)
C(15)	23(1)	22(1)	20(1)	-5(1)	-5(1)	2(1)
C(16)	26(1)	31(1)	24(1)	-9(1)	-10(1)	6(1)
C(17)	23(1)	30(1)	27(1)	-10(1)	-7(1)	-1(1)
C(18)	20(1)	48(1)	26(1)	-4(1)	-7(1)	-1(1)
C(19)	46(1)	44(1)	56(1)	7(1)	-20(1)	7(1)
C(20)	44(1)	104(2)	38(1)	-16(1)	-24(1)	0(1)
C(21)	24(1)	19(1)	24(1)	-5(1)	-9(1)	2(1)
C(22)	29(1)	36(1)	41(1)	-8(1)	-16(1)	-4(1)
C(2)	24(1)	16(1)	25(1)	-3(1)	-9(1)	0(1)
C(3)	33(1)	23(1)	26(1)	-7(1)	-14(1)	1(1)
C(4)	32(1)	33(1)	19(1)	-9(1)	-5(1)	2(1)

Table 13: Hydrogen coordinates (x 10^4) and isotropic displacement parameters ($Å^2$x 10^3) for carboxylic acid **3**.

	x	y	z	U(eq)
H(2)	6210(20)	-61(11)	4093(10)	35
H(1)	7167	2536	2296	24
H(5)	8899	4313	-178	35
H(6A)	5186	4626	930	30
H(6B)	6676	5674	384	30
H(7)	7829	4792	2064	24
H(9A)	4636	6197	2178	30
H(9B)	5652	6018	3155	30
H(10)	8270	6917	1201	30
H(12A)	4248	9050	1389	69
H(12B)	4885	9237	2448	69
H(12C)	4045	7972	2518	69
H(13A)	9165	8885	438	58
H(13B)	8200	9692	1321	58
H(13C)	7576	9766	188	58
H(14A)	3025	4185	2778	36
H(14B)	3233	4666	3836	36
H(14C)	2836	3271	4011	36
H(15A)	6210	4530	4261	26
H(15B)	5406	3200	4835	26
H(16A)	8171	2373	4043	32
H(16B)	8994	3667	3290	32
H(17)	8802	4181	5108	31
H(19A)	8673	1068	5360	76
H(19B)	9319	761	6508	76
H(19C)	10783	1097	5257	76
H(20A)	11439	2704	6468	90
H(20B)	9495	2395	7402	90
H(20C)	9956	3728	6605	90
H(22A)	1810	2426	1426	51
H(22B)	704	1435	2532	51
H(22C)	2344	1043	1564	51
H(3)	4810	2077	416	31

H(4)	8075	2575	-367	34

Table 14: Torsion angles [°] for carboxylic acid **3**.

C(4)-C(5)-C(6)-C(7)	98.77(18)
C(5)-C(6)-C(7)-C(9)	155.06(12)
C(5)-C(6)-C(7)-C(8)	-79.95(15)
C(6)-C(7)-C(8)-C(14)	-78.99(14)
C(9)-C(7)-C(8)-C(14)	43.98(14)
C(6)-C(7)-C(8)-C(15)	162.64(11)
C(9)-C(7)-C(8)-C(15)	-74.39(13)
C(6)-C(7)-C(8)-C(1)	43.14(15)
C(9)-C(7)-C(8)-C(1)	166.11(11)
C(2)-C(1)-C(8)-C(14)	44.97(15)
C(21)-C(1)-C(8)-C(14)	-77.58(14)
C(2)-C(1)-C(8)-C(15)	164.09(11)
C(21)-C(1)-C(8)-C(15)	41.55(15)
C(2)-C(1)-C(8)-C(7)	-77.88(14)
C(21)-C(1)-C(8)-C(7)	159.58(11)
C(6)-C(7)-C(9)-C(10)	-69.05(15)
C(8)-C(7)-C(9)-C(10)	163.05(11)
C(7)-C(9)-C(10)-C(11)	165.94(14)
C(9)-C(10)-C(11)-C(12)	-2.6(2)
C(9)-C(10)-C(11)-C(13)	175.41(14)
C(14)-C(8)-C(15)-C(16)	167.54(12)
C(7)-C(8)-C(15)-C(16)	-72.77(14)
C(1)-C(8)-C(15)-C(16)	47.92(15)
C(8)-C(15)-C(16)-C(17)	171.61(12)
C(15)-C(16)-C(17)-C(18)	124.77(16)
C(16)-C(17)-C(18)-C(19)	-0.4(2)
C(16)-C(17)-C(18)-C(20)	177.02(15)
C(2)-C(1)-C(21)-O(3)	-99.02(15)
C(8)-C(1)-C(21)-O(3)	30.39(19)
C(2)-C(1)-C(21)-O(2)	80.13(13)
C(8)-C(1)-C(21)-O(2)	-150.46(11)
C(22)-O(1)-C(2)-C(3)	-12.74(19)
C(22)-O(1)-C(2)-C(1)	172.82(11)
C(21)-C(1)-C(2)-C(3)	-130.96(14)
C(8)-C(1)-C(2)-C(3)	102.11(15)
C(21)-C(1)-C(2)-O(1)	43.47(13)
C(8)-C(1)-C(2)-O(1)	-83.46(14)

O(1)-C(2)-C(3)-C(4)	175.05(13)
C(1)-C(2)-C(3)-C(4)	-11.3(2)
C(6)-C(5)-C(4)-C(3)	-7.3(2)
C(2)-C(3)-C(4)-C(5)	-48.8(2)

Table 15: Hydrogen bonds for carboxylic acid **3** [Å und °].

D-H...A	d(D-H)	d(H...A)	d(D...A)	<(DHA)
O(2)-H(2)...O(3)#1	0.857(9)	1.823(9)	2.6793(13)	177.1(16)

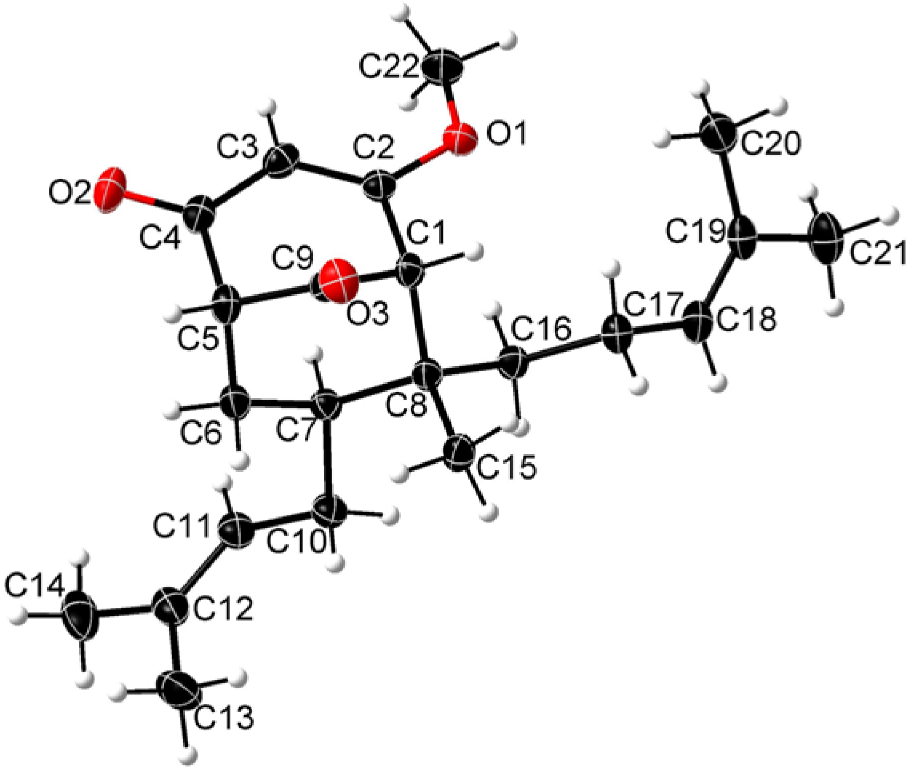

Molecular structure of the 1,3-diketone **16**. Displacement ellipsoids at a 50% probability level; hydrogen atoms are shown as grey spheres of arbitrary size.

Table 16: Crystal data and structure refinement for 1,3-diketone **16** (CCDC: 2362032).

Identification code	sh5174_a	
Empirical formula	C22 H32 O3	
Formula weight	344.47	
Temperature	143(2) K	
Wavelength	0.71073 Å	
Crystal system	Monoclinic	
Space group	$P2_1/c$	
Unit cell dimensions	a = 11.9491(4) Å	α = 90°.
	b = 7.3503(2) Å	β = 103.1200(10)°.
	c = 22.4943(7) Å	γ = 90°.
Volume	1924.09(10) $Å^3$	
Z	4	
Density (calculated)	1.189 Mg/m^3	
Absorption coefficient	0.077 mm^{-1}	

F(000)	752
Crystal size	0.080 x 0.080 x 0.020 mm^3
Theta range for data collection	2.246 to 27.119°.
Index ranges	-15<=h<=15, -9<=k<=9, -28<=l<=28
Reflections collected	25547
Independent reflections	4257 [R(int) = 0.0601]
Completeness to theta = 25.242°	99.9 %
Absorption correction	Semi-empirical from equivalents
Max. and min. transmission	0.7455 and 0.6871
Refinement method	Full-matrix least-squares on F^2
Data / restraints / parameters	4257 / 0 / 232
Goodness-of-fit on F^2	1.054
Final R indices [I>2sigma(I)]	R1 = 0.0450, wR2 = 0.0974
R indices (all data)	R1 = 0.0684, wR2 = 0.1109
Extinction coefficient	n/a
Largest diff. peak and hole	0.237 and -0.207 e.$Å^{-3}$

Table 17: Atomic coordinates (x 10^4) and equivalent isotropic displacement parameters ($Å^2$x 10^3) for 1,3-diketone **16**. U(eq) is defined as one third of the trace of the orthogonalized U^{ij} tensor.

	x	y	z	U(eq)
O(1)	9290(1)	5456(1)	4294(1)	24(1)
O(2)	7915(1)	10746(1)	5093(1)	31(1)
O(3)	7157(1)	5311(2)	5660(1)	29(1)
C(1)	7607(1)	5248(2)	4667(1)	20(1)
C(2)	8560(1)	6426(2)	4542(1)	21(1)
C(3)	8648(1)	8228(2)	4666(1)	24(1)
C(4)	7826(1)	9139(2)	4944(1)	24(1)
C(5)	6810(1)	8033(2)	5053(1)	23(1)
C(6)	5815(1)	8051(2)	4473(1)	22(1)
C(7)	6059(1)	7004(2)	3923(1)	20(1)
C(8)	6554(1)	5067(2)	4098(1)	19(1)
C(9)	7187(1)	6098(2)	5190(1)	22(1)
C(10)	4950(1)	7006(2)	3413(1)	26(1)
C(11)	4601(1)	8898(2)	3196(1)	26(1)
C(12)	3605(1)	9732(2)	3186(1)	30(1)
C(13)	2615(2)	8887(3)	3390(1)	42(1)
C(14)	3392(2)	11652(3)	2953(1)	43(1)
C(15)	5666(1)	3828(2)	4294(1)	24(1)
C(16)	6956(1)	4192(2)	3558(1)	21(1)
C(17)	7455(1)	2263(2)	3673(1)	24(1)
C(18)	7835(2)	1480(2)	3135(1)	26(1)
C(19)	8908(2)	1132(2)	3093(1)	26(1)
C(20)	9960(2)	1533(2)	3584(1)	33(1)
C(21)	9159(2)	255(2)	2532(1)	35(1)
C(22)	10136(1)	6452(2)	4059(1)	32(1)

Table 18: Bond lengths [Å] and angles [°] for 1,3-diketone **16**.

O(1)-C(2)	1.3415(17)
O(1)-C(22)	1.4422(18)
O(2)-C(4)	1.2257(18)
O(3)-C(9)	1.2124(18)
C(1)-C(2)	1.507(2)
C(1)-C(9)	1.516(2)
C(1)-C(8)	1.583(2)
C(2)-C(3)	1.352(2)
C(3)-C(4)	1.444(2)
C(4)-C(5)	1.526(2)
C(5)-C(9)	1.503(2)
C(5)-C(6)	1.553(2)
C(6)-C(7)	1.5395(19)
C(7)-C(10)	1.544(2)
C(7)-C(8)	1.5573(19)
C(8)-C(15)	1.537(2)
C(8)-C(16)	1.5431(19)
C(10)-C(11)	1.501(2)
C(11)-C(12)	1.334(2)
C(12)-C(13)	1.498(3)
C(12)-C(14)	1.506(2)
C(16)-C(17)	1.537(2)
C(17)-C(18)	1.501(2)
C(18)-C(19)	1.331(2)
C(19)-C(20)	1.502(2)
C(19)-C(21)	1.506(2)
C(2)-O(1)-C(22)	117.28(12)
C(2)-C(1)-C(9)	107.80(12)
C(2)-C(1)-C(8)	113.12(12)
C(9)-C(1)-C(8)	108.67(12)
O(1)-C(2)-C(3)	125.34(14)
O(1)-C(2)-C(1)	111.24(12)
C(3)-C(2)-C(1)	123.42(14)
C(2)-C(3)-C(4)	121.00(14)
O(2)-C(4)-C(3)	122.71(15)
O(2)-C(4)-C(5)	119.45(14)
C(3)-C(4)-C(5)	117.83(13)

C(9)-C(5)-C(4)	108.94(13)
C(9)-C(5)-C(6)	108.51(12)
C(4)-C(5)-C(6)	110.24(12)
C(7)-C(6)-C(5)	114.98(12)
C(6)-C(7)-C(10)	108.02(12)
C(6)-C(7)-C(8)	112.63(12)
C(10)-C(7)-C(8)	113.67(12)
C(15)-C(8)-C(16)	109.68(12)
C(15)-C(8)-C(7)	111.51(12)
C(16)-C(8)-C(7)	110.54(12)
C(15)-C(8)-C(1)	106.95(12)
C(16)-C(8)-C(1)	109.79(12)
C(7)-C(8)-C(1)	108.29(11)
O(3)-C(9)-C(5)	124.35(14)
O(3)-C(9)-C(1)	124.12(14)
C(5)-C(9)-C(1)	111.52(12)
C(11)-C(10)-C(7)	111.76(13)
C(12)-C(11)-C(10)	127.75(16)
C(11)-C(12)-C(13)	124.59(17)
C(11)-C(12)-C(14)	121.09(17)
C(13)-C(12)-C(14)	114.32(16)
C(17)-C(16)-C(8)	115.51(12)
C(18)-C(17)-C(16)	113.26(12)
C(19)-C(18)-C(17)	127.10(15)
C(18)-C(19)-C(20)	124.68(15)
C(18)-C(19)-C(21)	121.29(16)
C(20)-C(19)-C(21)	114.03(15)

Table 19: Anisotropic displacement parameters ($Å^2$x 10^3) for 1,3-diketone **16**. The anisotropic displacement factor exponent takes the form: $-2p^2[h^2 a^{*2}U^{11} + ... + 2 h k a^* b^* U^{12}]$.

	U^{11}	U^{22}	U^{33}	U^{23}	U^{13}	U^{12}
O(1)	21(1)	24(1)	28(1)	-1(1)	7(1)	0(1)
O(2)	42(1)	18(1)	31(1)	-3(1)	4(1)	-1(1)
O(3)	36(1)	29(1)	22(1)	6(1)	10(1)	4(1)
C(1)	23(1)	16(1)	19(1)	2(1)	4(1)	2(1)
C(2)	20(1)	22(1)	18(1)	0(1)	1(1)	2(1)
C(3)	26(1)	22(1)	22(1)	2(1)	2(1)	-3(1)
C(4)	31(1)	19(1)	18(1)	1(1)	-1(1)	2(1)
C(5)	29(1)	19(1)	20(1)	-2(1)	6(1)	2(1)
C(6)	24(1)	19(1)	24(1)	0(1)	5(1)	3(1)
C(7)	20(1)	19(1)	20(1)	0(1)	4(1)	2(1)
C(8)	21(1)	17(1)	20(1)	0(1)	4(1)	1(1)
C(9)	22(1)	22(1)	21(1)	0(1)	4(1)	-1(1)
C(10)	24(1)	26(1)	25(1)	-2(1)	1(1)	3(1)
C(11)	27(1)	28(1)	22(1)	0(1)	1(1)	3(1)
C(12)	30(1)	35(1)	21(1)	-7(1)	-3(1)	9(1)
C(13)	27(1)	55(1)	42(1)	-13(1)	6(1)	9(1)
C(14)	50(1)	38(1)	34(1)	-7(1)	-9(1)	20(1)
C(15)	27(1)	21(1)	28(1)	-2(1)	10(1)	-2(1)
C(16)	22(1)	20(1)	20(1)	-1(1)	5(1)	1(1)
C(17)	28(1)	19(1)	24(1)	-1(1)	8(1)	2(1)
C(18)	34(1)	21(1)	24(1)	-3(1)	6(1)	3(1)
C(19)	36(1)	16(1)	28(1)	1(1)	12(1)	4(1)
C(20)	31(1)	29(1)	39(1)	-1(1)	11(1)	3(1)
C(21)	48(1)	29(1)	35(1)	-3(1)	21(1)	6(1)
C(22)	25(1)	33(1)	39(1)	-1(1)	13(1)	-4(1)

Table 20: Hydrogen coordinates (x 10^4) and isotropic displacement parameters ($Å^2$x 10^3) for 1,3-diketone **16**.

	x	y	z	U(eq)
H(1)	7919	4008	4791	24
H(3)	9259	8904	4568	29
H(5)	6536	8557	5405	27
H(6A)	5122	7525	4578	27
H(6B)	5638	9331	4349	27
H(7)	6653	7704	3769	24
H(10A)	5074	6270	3065	31
H(10B)	4321	6435	3568	31
H(11)	5156	9584	3049	31
H(13A)	2416	9639	3711	63
H(13B)	2830	7665	3550	63
H(13C)	1952	8805	3043	63
H(14A)	2740	11667	2599	65
H(14B)	4079	12113	2834	65
H(14C)	3219	12424	3276	65
H(15A)	5108	3405	3931	37
H(15B)	5267	4510	4558	37
H(15C)	6058	2779	4518	37
H(16A)	6295	4152	3202	25
H(16B)	7547	4988	3449	25
H(17A)	8119	2292	4028	28
H(17B)	6865	1454	3778	28
H(18)	7246	1203	2786	32
H(20A)	10480	2316	3419	49
H(20B)	10351	392	3729	49
H(20C)	9734	2155	3924	49
H(21A)	9688	1026	2370	53
H(21B)	8441	108	2223	53
H(21C)	9511	-940	2640	53
H(22A)	10603	5599	3884	47
H(22B)	10632	7135	4391	47
H(22C)	9747	7300	3742	47

Table 21: Torsion angles [°] for 1,3-diketone **16**.

C(22)-O(1)-C(2)-C(3)	-9.6(2)
C(22)-O(1)-C(2)-C(1)	170.19(13)
C(9)-C(1)-C(2)-O(1)	153.19(12)
C(8)-C(1)-C(2)-O(1)	-86.63(14)
C(9)-C(1)-C(2)-C(3)	-26.98(19)
C(8)-C(1)-C(2)-C(3)	93.19(17)
O(1)-C(2)-C(3)-C(4)	-178.81(13)
C(1)-C(2)-C(3)-C(4)	1.4(2)
C(2)-C(3)-C(4)-O(2)	175.91(15)
C(2)-C(3)-C(4)-C(5)	-4.2(2)
O(2)-C(4)-C(5)-C(9)	-147.27(14)
C(3)-C(4)-C(5)-C(9)	32.82(18)
O(2)-C(4)-C(5)-C(6)	93.78(16)
C(3)-C(4)-C(5)-C(6)	-86.14(16)
C(9)-C(5)-C(6)-C(7)	-50.69(17)
C(4)-C(5)-C(6)-C(7)	68.54(16)
C(5)-C(6)-C(7)-C(10)	174.67(12)
C(5)-C(6)-C(7)-C(8)	48.29(17)
C(6)-C(7)-C(8)-C(15)	66.39(16)
C(10)-C(7)-C(8)-C(15)	-56.89(16)
C(6)-C(7)-C(8)-C(16)	-171.32(12)
C(10)-C(7)-C(8)-C(16)	65.40(16)
C(6)-C(7)-C(8)-C(1)	-51.01(15)
C(10)-C(7)-C(8)-C(1)	-174.30(12)
C(2)-C(1)-C(8)-C(15)	179.64(12)
C(9)-C(1)-C(8)-C(15)	-60.69(14)
C(2)-C(1)-C(8)-C(16)	60.70(15)
C(9)-C(1)-C(8)-C(16)	-179.62(11)
C(2)-C(1)-C(8)-C(7)	-60.07(15)
C(9)-C(1)-C(8)-C(7)	59.61(14)
C(4)-C(5)-C(9)-O(3)	120.20(16)
C(6)-C(5)-C(9)-O(3)	-119.77(16)
C(4)-C(5)-C(9)-C(1)	-59.99(16)
C(6)-C(5)-C(9)-C(1)	60.04(16)
C(2)-C(1)-C(9)-O(3)	-123.82(16)
C(8)-C(1)-C(9)-O(3)	113.24(16)
C(2)-C(1)-C(9)-C(5)	56.37(16)

C(8)-C(1)-C(9)-C(5)	-66.57(15)
C(6)-C(7)-C(10)-C(11)	62.59(16)
C(8)-C(7)-C(10)-C(11)	-171.64(13)
C(7)-C(10)-C(11)-C(12)	-122.89(17)
C(10)-C(11)-C(12)-C(13)	-0.7(3)
C(10)-C(11)-C(12)-C(14)	179.84(15)
C(15)-C(8)-C(16)-C(17)	-56.13(17)
C(7)-C(8)-C(16)-C(17)	-179.49(12)
C(1)-C(8)-C(16)-C(17)	61.10(16)
C(8)-C(16)-C(17)-C(18)	-179.81(13)
C(16)-C(17)-C(18)-C(19)	111.62(18)
C(17)-C(18)-C(19)-C(20)	-2.3(3)
C(17)-C(18)-C(19)-C(21)	176.91(15)
